# Exploring the Potential Biological Activities of Pyrazole-Based Schiff Bases as Anti-Diabetic, Anti-Alzheimer’s, Anti-Inflammatory, and Cytotoxic Agents: *In Vitro* Studies with Computational Predictions

**DOI:** 10.3390/ph17050655

**Published:** 2024-05-17

**Authors:** Ahmed M. Naglah, Abdulrahman A. Almehizia, Asma S. Al-Wasidi, Amirah Senaitan Alharbi, Mohammed H. Alqarni, Ashraf S. Hassan, Wael M. Aboulthana

**Affiliations:** 1 Drug Exploration and Development Chair (DEDC), Department of Pharmaceutical Chemistry, College of Pharmacy, King Saud University, P.O. Box 2457, Riyadh 11451, Saudi Arabia; mehizia@ksu.edu.sa; 2 Department of Chemistry, College of Science, Princess Nourah Bint Abdulrahman University, Riyadh 11671, Saudi Arabia; asalwasidi@pnu.edu.sa; 3 King Khalid Hospital, King Saud University Medical City, P.O. Box 7805, Riyadh 11472, Saudi Arabia; aalharbi1@ksu.edu.sa; 4 Department of Pharmacognosy, College of Pharmacy, Prince Sattam Bin Abdulaziz University, P.O. Box 173, Al-Kharj 11942, Saudi Arabia; m.alqarni@psau.edu.sa; 5 Organometallic and Organometalloid Chemistry Department, National Research Centre, Dokki 12622, Cairo, Egypt; 6 Biochemistry Department, Biotechnology Research Institute, National Research Centre, Dokki 12622, Cairo, Egypt; wmkamel83@hotmail.com

**Keywords:** Schiff bases, pyrazole molecule, bioactive agent, multi-target agents, enzymatic assays, computational prediction

## Abstract

In this innovative research, we aim to reveal pyrazole-based Schiff bases as new multi-target agents. In this context, we re-synthesized three sets of pyrazole-based Schiff bases, **5a**–**f**, **6a**–**f**, and **7a**–**f**, to evaluate their biological applications. The data from *in vitro* biological assays (including antioxidant and scavenging activities, anti-diabetes, anti-Alzheimer’s, and anti-inflammatory properties) of the pyrazole-based Schiff bases **5a**–**f**, **6a**–**f**, and **7a**–**f** showed that the six pyrazole-based Schiff bases **5a**, **5d**, **5e**, **5f**, **7a**, and **7f** possess the highest biological properties among the compounds evaluated. The cytotoxicity against lung (A549) and colon (Caco-2) human cancer types, as well as normal lung (WI-38) cell lines, was evaluated. The data from the cytotoxicity investigation demonstrated that the three Schiff bases **5d**, **5e**, and **7a** are active against lung (A549) cells, while the two Schiff bases **5e** and **7a** exhibited the highest cytotoxicity towards colon (Caco-2) cells. Additionally, the enzymatic activities against caspase-3 and Bcl-2 of the six pyrazole-based Schiff bases **5a**, **5d**, **5e**, **5f**, **7a**, and **7f** were evaluated. Furthermore, we assessed the *in silico* absorption, distribution, metabolism, and toxicity (ADMT) properties of the more potent pyrazole-based Schiff bases. After modifying the structures of the six pyrazole-based Schiff bases, we plan to further extend the studies in the future.

## 1. Introduction

Nowadays, various studies correlated to drug design focus on creating agents with a broad spectrum of physiological effects against diverse diseases. Various human diseases such as cancer, diabetes, Alzheimer’s disease, and inflammation have risks and relationships between them from varied causative factors and complications. Therefore, scientific cooperation is necessary to produce a molecule that possesses multiple activities. This strategy is called one drug–multiple targets or multi-target agents [1,2,3,4,5,6]. A literature survey revealed that many studies are based on this strategy. For example, the study by Loganathan and his team in 2024 referred to anthraquinone-connected coumarin derivatives that may act as multi-target agents [7]. Additionally, Almehizia and his colleagues’ research indicated that pyrazolo[1, 5-*a*]pyrimidine derivatives possess various biological activities against cancer, diabetes, Alzheimer’s disease, and inflammation. Therefore, they were suggested as multi-target agents [8].

**Figure 1:**
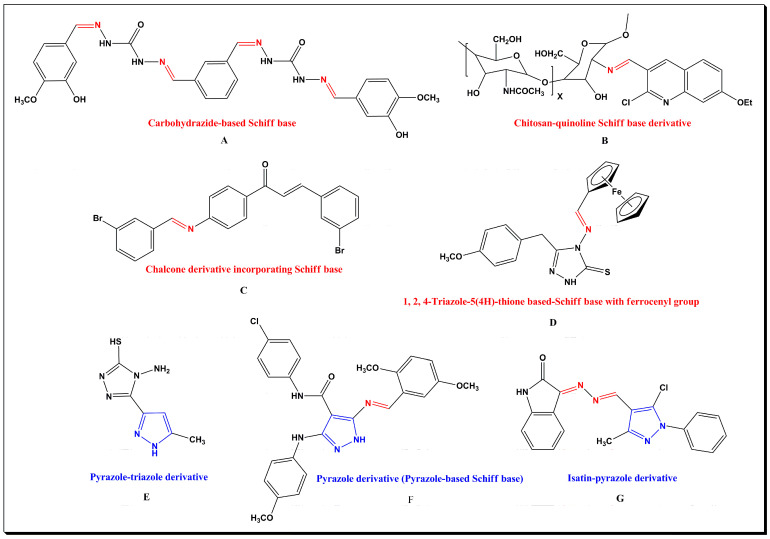
Bioactive derivatives of Schiff bases (**A**–**D**) and pyrazole scaffold (**E**–**G**).

Schiff bases are well known for their various biological effects [9], and numerous studies have established this [10,11,12,13]. In 2024, Çavuş et al. prepared carbohydrazide-based Schiff base **A**, which exhibits an antibacterial effect against *Bacillus cereus*, interacts with plasmid DNA, and has cytotoxic effects on HT-29 cell lines [14]. The study by Abdel-Baky et al. proved that chitosan–quinoline Schiff base derivative **B** possesses antibacterial and antioxidant effects and has high competence as an anti-diabetic agent through the inhibition of α-amylase and α-glucosidase enzymes [15]. The cooperation of Koçyiğit with his co-workers proved that a chalcone derivative incorporating Schiff base **C** acts as an acetylcholinesterase (AChE) inhibitor for treating Alzheimer’s disease [16]. In 2018, Hanif et al. prepared a 1, 2, 4-triazole-5(4*H*)-thione based-Schiff base with ferrocenyl group **D**, which acts as a potent anti-inflammatory and antioxidant agent [17] (Figure 1).

The pyrazole scaffold has received considerable attention because of its promising biological applications [18,19,20,21]. For examples, Mortada et al. (2024) synthesized a pyrazole-triazole derivative, **E**, that displayed powerful inhibition of α-glucosidase and α-amylase enzymes. Additionally, a pyrazole-triazole derivative showed an excellent antioxidant activity and radical scavenging abilities [22]. In 2023, Alkahtani et al. prepared a pyrazole derivative, **F**, (also an example of a pyrazole-based Schiff base) that acts as a multi-target agent against the α-amylase enzyme (an anti-diabetic agent), AChE enzyme (an anti-Alzheimer’s agent), and displays influential antioxidant properties [23]. From our previous work, the prepared isatin-pyrazole derivative **G** showed high antioxidant, anti-diabetes, anti-Alzheimer’s, and anti-arthritis properties [24] (Figure 1).

Based on the findings obtained from the previous studies mentioned above, such as the relationships between various human diseases, the biological effects of Schiff bases and the pyrazole scaffold, our research on multi-target agents, and the synthesis of biologically active heterocyclic compounds [25,26,27,28,29,30], we have been encouraged to re-synthesize three sets of pyrazole-based Schiff bases, **5a**–**f**, **6a**–**f**, and **7a**–**f**, from our previous works to evaluate their biological applications, including the antioxidant and scavenging activities, the anti-diabetes, anti-Alzheimer’s, anti-inflammatory, cytotoxicity, and enzymatic properties. Our goal is to find new candidates with multiple biological applications. Additionally, we assessed the *in silico* ADMT properties of the more potent pyrazole-based Schiff bases.

## 2. Results

### 2.1. Chemistry

**Scheme 1:**
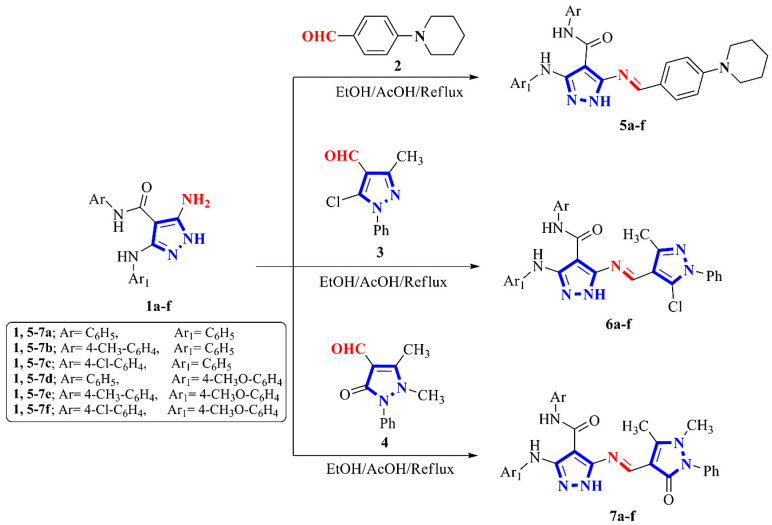
Synthesis of pyrazole-based Schiff bases **5a**–**f**, **6a**–**f**, and **7a**–**f.**

The various starting materials, 5-aminopyrazoles **1a**–**f** [31,32], 4-(piperidin-1-yl)benzaldehyde (**2**) [33], 5-chloro-3-methyl-1-phenyl-1*H*-pyrazole-4-carbaldehyde (**3**) [34], and 1,5-dimethyl-3-oxo-2-phenyl-2,3-dihydro-1*H*-pyrazole-4-carbaldehyde (**4**), were used as the basic materials for re-synthesizing the three sets of pyrazole-based Schiff bases. Here, we re-synthesized three sets of pyrazole-based Schiff bases (**5a**–**f**, **6a**–**f**, and **7a**–**f)** *via* the reaction of **1a**–**f** with **2**, **3**, and **4**, respectively, according to the literature procedures mentioned in our previous works [35,36] (Scheme 1).

### 2.2. Biological Evaluations

#### 2.2.1. The Antioxidant Activity

The antioxidant and scavenging activities of the pyrazole-based Schiff bases **5a**–**f**, **6a**–**f**, and **7a**–**f** were assessed at equal concentrations (Table 1). It was found that the compounds **5a**, **5d**, **5e**, **5f**, **7a**, and **7f** exhibited higher total antioxidant capacities (TACs) and iron-reducing power (IRP) than the other pyrazole-based Schiff bases. The compounds **5b** and **7e** showed the lowest TACs (15.26 ± 0.06 and 15.68 ± 0.06 mg gallic acid/g, respectively) and IRP (10.01 ± 0.02 and 10.29 ± 0.02 µg/mL, respectively).

Regarding the scavenging activity, it was noticed that the compounds **5a**, **5d**, **5e**, **5f**, **7a**, and **7f** had lower IC_50_ values for 1,1-diphenyl-2-picryl-hydrazyl (DPPH, 26.43 ± 0.01, 23.98 ± 0.01, 23.71 ± 0.01, 22.83 ± 0.01, 24.94 ± 0.01, and 21.37 ± 0.01 µM, respectively) and higher inhibition percentages against the 2, 2‘-azino-*bis*(3-ethylbenzothiazoline-6-sulfonic acid) (ABTS radical, 75.46 ± 0.05, 78.18 ± 0.05, 76.87 ± 0.05, 76.74 ± 0.05, 75.59 ± 0.05, and 78.05 ± 0.05%, respectively) compared to the other pyrazole-based Schiff bases. Furthermore, the highest IC_50_ values for DPPH were noticed with the compounds **5b** and **7e** (42.45 ± 0.02 and 51.98 ± 0.03 µM, respectively), and they showed the lowest inhibition percentages against the ABTS radical (22.03 ± 0.01 and 22.65 ± 0.01%, respectively).

#### 2.2.2. The Anti-Diabetic Activity

**Figure 2:**
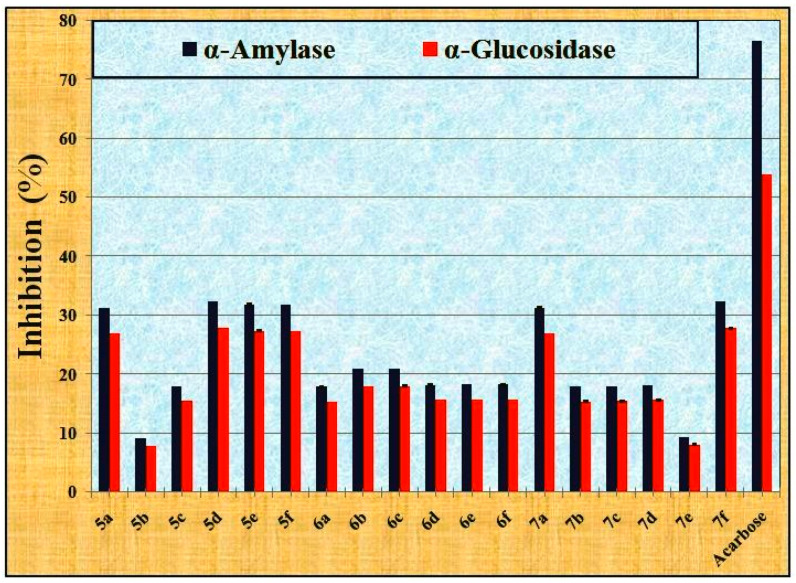
The anti-diabetic activities of pyrazole-based Schiff bases **5a**–**f**, **6a**–**f**, **7a**–**f**, and acarbose (anti-diabetic standard).

The anti-diabetic activities of the pyrazole-based Schiff bases **5a**–**f**, **6a**–**f**, and **7a**–**f** were assessed in the present study by determining their inhibitory effects against α-amylase and α-glucosidase (Figure 2, see the Supplementary Materials Table S1). It was observed that the pyrazole-based Schiff bases **5a**, **5d**, **5e**, **5f**, **7a**, and **7f** possessed a higher inhibitory effect on α-amylase (31.24 ± 0.05, 32.37 ± 0.05, 31.83 ± 0.05, 31.77 ± 0.05, 31.30 ± 0.05, 32.31 ± 0.05%, respectively) and α-glucosidase (26.87 ± 0.05, 27.84 ± 0.05, 27.37 ± 0.05, 27.33 ± 0.05, 26.91 ± 0.05, and 27.79 ± 0.05%, respectively) compared to the efficiency of acarbose against α-amylase (76.58 ± 0.01%) and α-glucosidase activities (53.94 ± 0.01%) at equal concentrations. Regarding the compounds **5b** and **7e**, it was found that they have the lowest inhibition percentages against α-amylase (9.12 ± 0.01 and 9.38 ± 0.02%, respectively) and α-glucosidase (7.85 ± 0.01 and 8.07 ± 0.02%, respectively).

#### 2.2.3. The Anti-Alzheimer’s Activity

**Figure 3:**
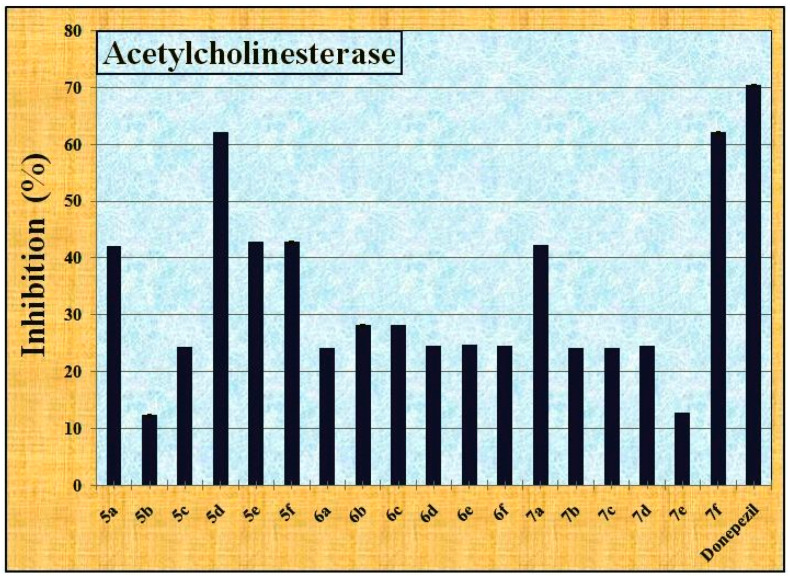
The anti-Alzheimer’s activities of pyrazole-based Schiff bases **5a**–**f**, **6a**–**f**, **7a**–**f**, and donepezil (anti-Alzheimer’s standard).

The anti-Alzheimer’s activities of the pyrazole-based Schiff bases **5a**–**f**, **6a**–**f**, and **7a**–**f** were assessed by determining their inhibitory effects against AChE (Figure 3, see the Supplementary Materials Table S2). It was found that the two pyrazole-based Schiff bases **7f** and **5d** possessed a higher inhibitory effect on AChE (62.11 ± 0.04 and 62.00 ± 0.04%, respectively) compared to the efficiency of donepezil (70.32 ± 0.04%) at the same concentration. On the contrary, the compounds **5b** and **7e** exhibited the lowest inhibition percentages against AChE (12.28 ± 0.01 and 12.62 ± 0.01%, respectively).

#### 2.2.4. The Anti-Inflammatory Activity

The anti-inflammatory activities of the pyrazole-based Schiff bases **5a**–**f**, **6a**–**f**, and **7a**–**f** were evaluated by determining their inhibitory effects against protein denaturation and a proteinase enzyme (Table 2, see the Supplementary Materials Figure S1). It was found that the pyrazole-based Schiff bases **5a**, **5d**, **5e**, **5f**, **7a**, and **7f** possessed a higher inhibitory effect on protein denaturation (26.83 ± 0.03, 27.79 ± 0.03, 27.33 ± 0.03, 27.28 ± 0.03, 26.87 ± 0.03, and 27.75 ± 0.03%, respectively) and the activity of the proteinase enzyme (23.33 ± 0.03, 24.17 ± 0.03, 23.76 ± 0.03, 23.72 ± 0.03, 23.37 ± 0.03, and 24.13 ± 0.03%, respectively) compared to the efficiency of diclofenac sodium against both protein denaturation and the proteinase enzyme (49.08 ± 0.01 and 46.11 ± 0.02%, respectively) at equal concentrations. On the contrary, the lowest inhibitory activities were observed with the compounds **5b** and **7e**, which inhibited both protein denaturation by 7.83 ± 0.01 and 8.05 ± 0.01%, respectively, and the activity of the proteinase enzyme by 6.81 ± 0.01 and 4.81 ± 0.01%, respectively.

#### 2.2.5. Cytotoxic Activity

Based on the other *in vitro* biological activities, it was found that **5a**, **5d**, **5e**, **5f**, **7a**, and **7f** exhibited the highest antioxidant, scavenging, anti-diabetic, anti-Alzheimer’s, and anti-inflammatory activities compared to the other pyrazole-based Schiff bases. Therefore, the cytotoxicities of these derivatives were evaluated against human lung (A549) and colon (Caco-2) cancer types as well as normal Caucasian fibroblast-like fetal lung (WI-38) cell lines (Table 3; see the Supplementary Materials Tables S3–S5).

It was found that the three pyrazole-based Schiff bases **5d**, **5e**, and **7a** were very active and exhibited the highest cytotoxic activities against the growth of the A549 cell line, resulting in the most significant decreases in cell viability. This was evident from their lower IC_50_ values (48.61 ± 0.14, 47.74 ± 0.20, and 49.40 ± 0.18 μM, respectively) compared to the other pyrazole-based Schiff bases. Regarding the Caco-2 cell line, the two pyrazole-based Schiff bases **5e** and **7a** exhibited the highest cytotoxic activities with the lowest IC_50_ values (40.99 ± 0.20 and 42.42 ± 0.18 μM, respectively). These pyrazole-based Schiff bases showed higher cytotoxic activities compared to doxorubicin, which was used as a reference drug (IC_50_ = 54.94 ± 0.16 μM).

The WI-38 cell line used to reveal the deleterious effects showed that compounds **5d** and **7a** demonstrated higher IC_50_ values (731.72 ± 10.46 and 736.26 ± 7.95 μM, respectively), followed by **5e** (IC_50_ = 648.12 ± 7.57 μM). This indicates the safety of these compounds for normal cells compared to the other pyrazole-based Schiff bases and doxorubicin, which was used as a reference drug (IC_50_ = 304.94 ± 4.72 μM).

The therapeutic indexes (TIs) of the pyrazole-based Schiff bases **5a**, **5d**, **5e**, **5f**, **7a**, and **7f** were determined to study their safety and efficacy (Table 3). The results demonstrated that all the pyrazole-based Schiff bases (**5a**, **5d**, **5e**, **5f**, **7a**, and **7f)** possessed a therapeutic index higher than doxorubicin in the case of Caco-2 cells. However, in the case of A549 cells, all Schiff bases possessed a therapeutic index higher than doxorubicin except for two Schiff bases: **5a** and **5f**.

#### 2.2.6. The Enzymatic Activity

The efficiencies of six pyrazole-based Schiff bases, **5a**, **5d**, **5e**, **5f**, **7a**, and **7f**, against caspase-3 and Bcl-2 were evaluated (Table 4).

It was observed that the two pyrazole-based Schiff bases **5d** and **7a** increased the activity of caspase-3 (300.73 ± 0.42 and 322.21 ± 0.45 pg/mL, respectively) while decreasing the Bcl-2 level (2.62 ± 0.01 and 2.81 ± 0.01 ng/mL, respectively) in the treated A549 cells compared to the untreated A549 cells. It showed approximately the same effect induced by doxorubicin on both the caspase-3 and Bcl-2 levels (330.80 ± 0.46 pg/mL and 2.55 ± 0.01 ng/mL, respectively). The pyrazole-based Schiff base **5a** showed the lowest anti-proliferative effect, as indicated by the lower caspase-3 level (184.74 ± 0.26 pg/mL) and the higher Bcl-2 level (6.55 ± 0.03 ng/mL).

Regarding the Caco-2 cell lines, it was observed that the two pyrazole-based Schiff bases **5e** and **7a** elevated the activity of caspase-3 (315.45 ± 0.46 and 363.98 ± 0.54 pg/mL, respectively) while decreasing the Bcl-2 level (2.13 ± 0.02 and 2.46 ± 0.02 ng/mL, respectively) in the treated cells compared to the untreated ones. It showed approximately the same effect induced by doxorubicin on both the caspase-3 and Bcl-2 levels (363.98 ± 0.54 pg/mL and 2.55 ± 0.01 ng/mL, respectively). The pyrazole-based Schiff base **5a** showed the lowest anti-proliferative effect, as indicated by the lower caspase-3 (208.68 ± 0.31 pg/mL) and the higher Bcl-2 levels (5.33 ± 0.05 ng/mL).

### 2.3. Computational Prediction (ADMT Properties)

Six derivatives of pyrazole-based Schiff bases (**5a**, **5d**, **5e**, **5f**, **7a**, and **7f**) were analyzed using the pkCSM website https://biosig.lab.uq.edu.au/pkcsm/prediction (accessed on 15 March 2024) to evaluate their properties and safety as potential drug candidates. The data presented in Table 5 showed the absorptions, distributions, metabolisms, and toxicities of these compounds. Also, the ideal values for all properties were presented.

It was observed that the intestinal absorption (human) range of the six derivatives of pyrazole-based Schiff bases (**5a**, **5d**, **5e**, **5f**, **7a**, and **7f**) was from 91.357% to 96.711%. The skin permeability value is log Kp = −2.735. The blood–brain permeation barrier (BBB permeability) ranged from −1.818 to −1.318. The central nervous system permeability (CNS permeability) ranged from −2.133 to −1.705. The results of the enzyme inhibition of the six Schiff bases (**5a**, **5d**, **5e**, **5f**, **7a**, and **7f**) show that they can be inhibitors or non-inhibitors. The two toxicity endpoints are AMES toxicity and skin sensitization.

## 3. Discussion

All the pyrazole-based Schiff bases (**5a**–**f**, **6a**–**f**, and **7a**–**f)** have been characterized utilizing spectral analyses (^1^H NMR and ^13^C NMR spectra) as mentioned in our previous works [35,36]. The ^1^H NMR spectra of the pyrazole-based Schiff bases **5a**–**f**, **6a**–**f**, and **7a**–**f** were characterized by a single signal in the range of δ from 8.64 to 8.93 ppm. This signal corresponds to an azomethine proton (–N=CH– proton) (See the Supplementary Materials).

The antioxidant activities of the pyrazole-based Schiff bases **5a**–**f**, **6a**–**f**, and **7a**–**f** were assessed at equal concentrations by quantifying their TACs and IRP. The compounds **5a**, **5d**, **5e**, **5f**, **7a**, and **7f** showed higher TACs and IRP, and this finding is consistent with Ali et al. [37], who demonstrated that the antioxidant activity increased in the cyclized heterocyclic compounds due to the presence of a pyrazoline moiety, which has potent antioxidant activity, and the low number of rotatable bonds makes them more favorable compared to the other compounds. In addition, the electron-donating groups (OCH_3_ and CH_3_) are more beneficial than unsubstituted or mono chloro-substituted phenyl rings, which might be attributed to the mesomeric effects [38]. The compounds **5b** and **7e** showed the lowest TACs and IRP, and this might be related to the replacement of the N atom by the O atom, which gives a lower antioxidant activity [39]. During the current study, the scavenging activity was assessed by calculating the IC_50_ values of DPPH and the inhibition percentages of ABTS radicals. The compounds with a higher antioxidant activity were found to have lower IC_50_ values of DPPH and a higher inhibition percentage against ABTS radicals [40]. It was found that the ABTS assay is more sensitive than the DPPH assay, because the DPPH radical is only involved in hydrogen (H^+^) transfer (DPPH to DPPH-H), while the ABTS radical is involved in the electron transfer pathway (ABTS to ABTS^+^) [41].

The compounds **5a**, **5d**, **5e**, **5f**, **7a**, and **7f** were found to have lower IC_50_ values for DPPH and higher inhibition percentages against the ABTS radical compared to the other pyrazole-based Schiff bases. This might be attributed to the affinity of these compounds to donate hydrogen free radicals as proposed by Matta et al. (in 2023) [42].

The highest IC_50_ values for DPPH were noticed with the compounds **5b** and **7e**, and they showed the lowest inhibition percentages against the ABTS radical. This indicates a lower scavenging activity, which may be due to the presence of only an aromatic group attached to the compound. This lack of significant inhibitory activity towards *in vitro* antioxidant and scavenging activities suggests that the presence of substituents is important for these activities [43].

Type 2 diabetes mellitus (T2DM) is the most common form of diabetes, leading to the impairment of various physiological processes in the body due to hyperglycemia or abnormal blood glucose levels induced by insulin resistance [44]. The development of new anti-diabetic drugs is related to the inhibition of hydrolase enzymes, showing that the incorporation of pyrazole is required in the design of new anti-hyperglycemic agents with higher activities than acarbose [45]. Therefore, the anti-diabetic activities of the pyrazole-based Schiff bases **5a**–**f**, **6a**–**f**, and **7a**–**f** were assessed in the present study by determining their inhibitory effects against α-amylase and α-glucosidase enzymes and comparing them to the efficiency of acarbose, which is used as a standard drug. In the present study, it was noticed that the pyrazole-based Schiff bases **5a**, **5d**, **5e**, **5f**, **7a**, and **7f** possessed a higher inhibitory effect on α-amylase and α-glucosidase compared to the efficiency of acarbose against α-amylase and α-glucosidase activities at equal concentrations. This may be attributed to the hydrogen bond interaction between the unsubstituted nitrogen atom of the pyrazole ring and the functional groups in the side chains of the amino acids at the active sites, which can also play a role [46]. Pogaku et al. added that pyrazole-based Schiff bases substituted with electron-withdrawing groups on the phenyl ring showed higher inhibitory activities against these hydrolase enzymes than compounds with electron-donating groups [47]. Moreover, these pyrazole-based Schiff bases showed their anti-diabetic activity through the formation of hydrophobic, van der Waals, and hydrogen bond interactions between the nitrogen atom of their pyrazole moiety and the oxygen atom of various amino acids in the active site of these enzymes [48]. Regarding the compounds **5b** and **7e**, it was found that they have the lowest inhibition percentages against α-amylase and α-glucosidase, and this might be related to their lower antioxidant and scavenging activities [49].

Alzheimer’s disease (AD) is a degenerative disease of the central nervous system characterized by mental deterioration, especially in the elderly [50]. According to “the cholinergic hypothesis”, which means inadequate cholinergic transmission in the synapse, AChE is the main enzyme responsible for acetylcholine (ACh) hydrolysis in the cholinergic synapses, constituting the basis of AD treatment [51]. Many studies have suggested that various therapeutic agents are capable of inhibiting the AChE enzyme and providing additional benefits for the treatment of AD, replacing the commercially available drugs, which only have symptomatic effects. Therefore, recent studies have focused on searching for more effective synthetic compounds to halt the progression of the disease [52].

The current study showed that the two pyrazole-based Schiff bases **7f** and **5d** possessed a higher inhibitory effect on AChE compared to the efficiency of donepezil at the same concentration, and this might be related to the presence of active substituents, which elicited a higher neuroprotective activity [53]. Coupling the phenyl group to the pyrazole scaffold through an imine linker resulted in the inhibitory activity, which varied with the substituent on the imine nitrogen due to its high affinity to bind to the active site of the AChE enzyme involved in the substrate inhibition characteristics of AChE. Furthermore, the phenyl group placed at this nitrogen was highly favorable for AChE inhibitory activity. Additionally, the addition of a hydroxyl group enhanced the activity [54]. The compounds **5b** and **7e** exhibited the lowest inhibition percentages against AChE, and this might be related to increasing the length of the alkyl substituent [55].

Arthritis is a chronic and progressive autoimmune disease in which bone and cartilage destruction occur due to chronic proliferative synovitis and synovial inflammation, resulting in significant joint damage and reduced functionality [56]. Protein denaturation is considered one of the predominant reasons for arthritic and inflammatory diseases [57]. Therefore, synthetic compounds such as pyrazole-based Schiff bases that can inhibit both protein denaturation and proteinase enzyme are a potential strategy for arthritic therapy [58,59]. The present study showed that the pyrazole-based Schiff bases **5a**, **5d**, **5e**, **5f**, **7a**, and **7f** possessed a higher inhibitory effect on both protein denaturation and the activity of the proteinase enzyme compared to the efficiency of diclofenac sodium at equal concentrations. This might refer to altering the tertiary as well as secondary structures of proteins, which consequently leads to impairments in the biological functions of most biological proteins. Moreover, the presence of a chemical entity possessing a substituent exerts the highest activity compared with all other compounds due to it binding effectively toward the active site of the proteins [60,61]. Joy et al. proposed that the anti-denaturation activity can be rationalized by the presence of electron-donating groups (–OH and –NH_2_) and nitrogen-rich fragments, which exhibit a superior anti-inflammatory potential compared to the other pyrazole-based Schiff bases molecules [62]. The lowest inhibitory activities were observed with compounds **5b** and **7e**, which inhibited both protein denaturation and the activity of the proteinase enzyme. This could possibly be due to the presence of substituents that affected the activities and were found to be the least active in this series [61].

Finally, based on the evaluations of the pyrazole-based Schiff bases **5a**–**f**, **6a**–**f**, and **7a**–**f**, including their antioxidant and scavenging activities, anti-diabetes, anti-Alzheimer’s, and anti-inflammatory properties, it can be deduced that the six pyrazole-based Schiff bases **5a**, **5d**, **5e**, **5f**, **7a**, and **7f** exhibit highly active properties in these evaluations. Therefore, further studies on the cytotoxic and enzymatic activities of these six pyrazole-based Schiff bases are warranted.

Carcinoma is the abnormal growth of normal cells that typically grow beyond their original boundaries, invade surrounding areas, spread to other organs, and result in metastasis, which is one of the main causes of cancer-related deaths [63]. The present study showed that the three pyrazole-based Schiff bases **5d**, **5e**, and **7a** were very active and exhibited the highest cytotoxic activities against the growth of the A549 cell line, resulting in the most significant decreases in cell viability. This finding is consistent with Czylkowska et al., who postulated that pyrazole derivatives are capable of effectively inhibiting the growth of the A549 cell line, possibly due to the introduction of electron-withdrawing substitutions that increase the lipophilicity of the molecule [64]. This enhancement in lipophilicity may improve the cell permeability and overall potency of these compounds [65]. Furthermore, these substituted pyrazole derivatives might be able to arrest the cell cycle at the G1/S phase in treated cells, displaying an accumulation of cells in the G0 phase and an increase in the percentage of cells in both the early and late apoptotic stages [66]. Therefore, these synthetic compounds may hold promise for the treatment of lung cancer. In regard to the Caco-2 cell line, the two pyrazole-based Schiff bases **5e** and **7a** exhibited the highest cytotoxic activities with the lowest IC_50_ values, and this may be attributed to the ability of the pyrazoline derivatives, which exhibit cell cycle-arrest properties, to induce cell cycle arrest in the G2/M phase and apoptosis [67]. Additionally, the pyrazole-based Schiff bases may have the ability to bind to DNA through intercalation, similar to doxorubicin [68].

Regarding the WI-38 cell line, the compounds **5d** and **7a** showed higher IC_50_ values followed by **5e**, indicating the safety of these compounds for normal cells compared to the other pyrazole-based Schiff bases and doxorubicin.

Cysteine proteases, specifically caspases, play a vital role in programmed cell death by coordinating the cascade for degrading cellular components [69]. Caspase-3, a member of the cysteine–aspartate-specific protease family, is a ubiquitous protein in mammalian cells that contributes to apoptosis [70]. It activates the apoptosis pathway in response to a variety of stimuli, including chemotherapeutic compounds [71]. The B-cell lymphoma 2 (Bcl-2) belongs to the Bcl-2 protein family, which exhibits pro- and anti-apoptotic activities and is held in a delicate balance in healthy cells. It can cause cells to irreversibly head toward cell death or, conversely, allow cells to permanently escape apoptosis and become a malignant clone [72].

The present study showed that the two pyrazole-based Schiff bases **5d** and **7a** increased the activity of caspase-3 while decreasing the Bcl-2 level in the treated A549 cells compared to the untreated A549 cells. The pyrazole-based Schiff base **5a** showed the lowest anti-proliferative effect, as indicated by the lower caspase-3 level and the higher Bcl-2 level. Furthermore, the two pyrazole-based Schiff bases **5e** and **7a** elevated the activity of caspase-3 while decreasing the Bcl-2 level in the treated Caco-2 cells compared to the untreated ones. The pyrazole-based Schiff base **5a** showed the lowest anti-proliferative effect, as indicated by the lower caspase-3 and the higher Bcl-2 levels. The overall enzymatic assay showed that the pyrazole-based Schiff base **7a** exhibited the highest anti-proliferative effect against both A549 and Caco-2 cell lines, while the pyrazole-based Schiff base **5a** showed the lowest effect. The pyrazole-based Schiff bases, especially **7a**, showed an anti-proliferative effect on both studied cell lines by stimulating the apoptotic pathway through the upregulation of caspase-3 and downregulation of Bcl-2 [73].

The pyrazole-based Schiff bases (**5a**, **5d**, **5e**, **5f**, **7a**, and **7f**) were analyzed using the pkCSM website [74,75], and the data presented in Table 5 demonstrate the following:

- The absorption results of the pyrazole-based Schiff bases (**5a**, **5d**, **5e**, **5f**, **7a**, and **7f**) indicate high values (well-absorbed molecules) ranging from 91.357% to 96.711% for intestinal absorption. Skin permeability (Kp) refers to skin absorption and the rate of drug candidates penetrating the skin. The skin permeability of the Schiff bases indicates low values less than −2.5, therefore, showing good skin permeability (log Kp = −2.735) and the ability to penetrate through the outermost layer of the epidermal skin.
- The distribution results conclude that (i) the blood–brain permeation barrier (BBB permeability) predicted results indicate that these Schiff bases exhibit a poor distribution, with values lower than −1. The blood–brain barrier (BBB) regulates the permeability of drugs to the brain. A poor distribution refers to impaired drug delivery into the brain. Therefore, the medicinal efficacy of the drugs decreases. (ii) The central nervous system permeability (CNS permeability) indicates that the four Schiff bases (**5a**, **5d**, **5e**, and **5f**) show high penetration, but two Schiff bases (**7a** and **7f**) show moderate penetration.
- Drug metabolism is one of the essential factors in drug disposition. The five enzymes play a crucial role in the metabolic processes of drugs in the liver. The results indicated that the six Schiff bases (**5a**, **5d**, **5e**, **5f**, **7a**, and **7f**) are non-inhibitors of the CYP2D6 enzyme. Also, three Schiff bases (**5e**, **5f**, and **7f**) are non-inhibitors of the CYP1A2 enzyme. Therefore, the Schiff bases are well-metabolized molecules in the liver, can be eliminated from the body, and have no potential adverse effects.
- The prediction of the toxicity of pyrazole-based Schiff bases (**5a**, **5d**, **5e**, **5f**, **7a**, and **7f**) suggests that all Schiff bases are non-mutagenic except **7f**. Additionally, none of the Schiff bases (**5a**, **5d**, **5e**, **5f**, **7a**, and **7f**) induce skin sensitization. Therefore, these compounds are considered safe.

Almost all Schiff bases possess promising ADMT properties (well absorbed, possess good skin permeability, and well metabolized in the liver), which may be due to the presence of the pyrazole moiety in their structures. The pyrazole motif has promising biological applications [18,19,20,21] and is effective in various disease treatments.

After the *in silico* ADMT properties study, in the future, we will extend the study to include molecular docking and simulation for caspase-3 and Bcl-2 enzymes.

## 4. Materials and Methods

### 4.1. Chemistry

The three sets of pyrazole-based Schiff bases, **5a**–**f**, **6a**–**f**, and **7a**–**f**, were prepared according to the literature procedures mentioned in our previous works. All the pyrazole-based Schiff bases (**5a**–**f**, **6a**–**f**, and **7a**–**f)** have been characterized using spectral analyses. These spectral data were mentioned in our previous works [35,36] (see the Supplementary Materials).

### 4.2. Biological Evaluations

All *in vitro* biological activities were assessed in the tested compounds at equal concentrations (1000 µg/mL). All assays were carried out in triplicate.

#### 4.2.1. The Antioxidant Activity

The antioxidant and scavenging activities of the pyrazole-based Schiff bases **5a**–**f**, **6a**–**f**, and **7a**–**f** were assessed at equal concentrations according to the reported techniques in the literature [76,77,78,79]

#### 4.2.2. The Anti-Diabetic Activity

The anti-diabetic activities of the pyrazole-based Schiff bases **5a**–**f**, **6a**–**f**, and **7a**–**f** were assessed in the present study by determining their inhibitory effects against α-amylase and α-glucosidase according to the reported techniques in the literature [80,81]

#### 4.2.3. The Anti-Alzheimer’s Activity

The anti-Alzheimer’s activities of the pyrazole-based Schiff bases **5a**–**f**, **6a**–**f**, and **7a**–**f** were assessed by determining their inhibitory effects against acetylcholinesterase (AChE) according to the reported techniques in the literature [82].

#### 4.2.4. The Anti-Inflammatory Activity

The anti-inflammatory activities of the pyrazole-based Schiff bases **5a**–**f**, **6a**–**f**, and **7a**–**f** were evaluated by determining their inhibitory effects against both protein denaturation and proteinase enzyme according to the reported techniques in the literature [83,84,85].

#### 4.2.5. Cytotoxic Activity

The cytotoxicity of six pyrazole-based Schiff bases, **5a**, **5d**, **5e**, **5f**, **7a**, and **7f**, against human lung (A549) and colon (Caco-2) cancer types, as well as normal lung (WI-38) cell lines, was evaluated according to the reported techniques in the literature [86]. The therapeutic indexes (TIs) of the compounds were calculated according to the following equation:

Therapeutic index (TI) = IC_50_ on the normal cells/IC_50_ on the cancer cells

#### 4.2.6. The Enzymatic Activity

The enzymatic activities of six pyrazole-based Schiff bases, **5a**, **5d**, **5e**, **5f**, **7a**, and **7f**, against caspase-3 and Bcl-2 were evaluated according to the reported techniques in the literature [87,88].

#### 4.2.7. Statistical Analysis

The data were calculated from three replicates and presented as the mean ± SE.

The detailed methods for the *in vitro* biological activities have been added to the Supplementary Materials.

## 5. Conclusions

In summary, we re-synthesized three sets of pyrazole-based Schiff bases, **5a**–**f**, **6a**–**f**, and **7a**–**f**, to evaluate their biological applications. The results of the biological evaluations of the pyrazole-based Schiff bases **5a**–**f**, **6a**–**f**, and **7a**–**f** demonstrated that the six pyrazole-based Schiff bases, **5a**, **5d**, **5e**, **5f**, **7a**, and **7f**, possessed the highest biological properties among the compounds evaluated and acted as multi-target agents against diabetes, Alzheimer’s, and inflammatory diseases. As a result, the biological evaluation was extended, namely the cytotoxic and enzymatic activities of the six pyrazole-based Schiff bases **5a**, **5d**, **5e**, **5f**, **7a**, and **7f**. The cytotoxicity study showed that the three Schiff bases **5d**, **5e**, and **7a** were very active against lung (A549) cells, with IC_50_ values of 48.61 ± 0.14, 47.74 ± 0.20, and 49.40 ± 0.18 μM, respectively. Concerning the colon (Caco-2) cells, the two Schiff bases **5e** and **7a** exhibited the highest cytotoxicity, with IC_50_ values of 40.99 ± 0.20 and 42.42 ± 0.18 μM, respectively. The safety and efficacy study showed that all pyrazole-based Schiff bases (**5a**, **5d**, **5e**, **5f**, **7a**, and **7f)** possessed a therapeutic index higher than doxorubicin. The enzymatic activity study indicated that some Schiff bases affected the levels of caspase-3 and Bcl-2 in both A549 and Caco-2 cells. Additionally, we assessed the *in silico* ADMT properties of the pyrazole-based Schiff bases **5a**, **5d**, **5e**, **5f**, **7a**, and **7f**. The results demonstrate that Schiff bases are well absorbed, have good skin permeability, poor distribution through the BBB permeability, high or moderate penetration through CNS permeability, are non-inhibitors of the CYP2D6 enzyme, are non-mutagenic except for **7f**, and do not induce skin sensitization. Currently, in our laboratories, with teams of colleagues, we are conducting further biochemistry, medicinal chemistry, molecular docking, and simulation studies on the six pyrazole-based Schiff bases **5a**, **5d**, **5e**, **5f**, **7a**, and **7f** after modifying their structures. In the future, we will report the results.

## Figures and Tables

**Table 1:** Antioxidant and scavenging activities of pyrazole-based Schiff bases **5a**–**f**, **6a**–**f**, and **7a**–**f**.

Pyrazole-Based Schiff Bases	Antioxidant Activity		Scavenging Activity	
	Total Antioxidant Capacity (TAC, mg Gallic Acid/g)	Iron-Reducing Power (IRP, µg/mL)	DPPH (IC_50_ µM)	ABTS (%)
**5a** *	52.25 ± 0.19	34.27 ± 0.07	26.43 ± 0.01	75.46 ± 0.05
**5b**	15.26 ± 0.06	10.01 ± 0.02	59.80 ± 0.03	22.03 ± 0.01
**5c**	30.01 ± 0.11	19.68 ± 0.04	42.86 ± 0.02	43.34 ± 0.03
**5d** *	54.13 ± 0.20	35.51 ± 0.07	23.98 ± 0.01	78.18 ± 0.05
**5e** *	53.23 ± 0.20	34.91 ± 0.07	23.71 ± 0.01	76.87 ± 0.05
**5f** *	53.14 ± 0.20	34.85 ± 0.07	22.83 ± 0.01	76.74 ± 0.05
**6a**	29.86 ± 0.11	19.58 ± 0.04	43.35 ± 0.02	43.12 ± 0.03
**6b**	34.93 ± 0.13	22.91 ± 0.04	36.02 ± 0.02	50.45 ± 0.03
**6c**	34.99 ± 0.13	22.95 ± 0.05	34.58 ± 0.02	50.54 ± 0.03
**6d**	30.42 ± 0.11	19.95 ± 0.04	40.11 ± 0.02	43.93 ± 0.03
**6e**	30.52 ± 0.11	20.02 ± 0.04	38.94 ± 0.02	44.08 ± 0.03
**6f**	30.47 ± 0.11	19.98 ± 0.04	37.60 ± 0.02	44.00 ± 0.03
**7a** *	52.34 ± 0.19	34.33 ± 0.07	24.94 ± 0.01	75.59 ± 0.05
**7b**	29.91 ± 0.11	19.62 ± 0.04	42.45 ± 0.02	43.19 ± 0.03
**7c**	29.96 ± 0.11	19.65 ± 0.04	40.72 ± 0.02	43.27 ± 0.03
**7d**	30.36 ± 0.11	19.92 ± 0.04	40.53 ± 0.02	43.85 ± 0.03
**7e**	15.68 ± 0.06	10.29 ± 0.02	51.98 ± 0.03	22.65 ± 0.01
**7f** *	54.04 ± 0.20	35.45 ± 0.07	21.37 ± 0.01	78.05 ± 0.05
**STD**	**Ascorbic Acid**		**Ascorbic Acid**	
	79.10 ± 0.11	71.85 ± 0.05	29.30 ± 0.01	41.25 ± 0.01

Data calculated from three replicates and presented as the mean ± SE. * Indicates the most potent pyrazole-based Schiff bases.

**Table 2:** The anti-inflammatory activities of pyrazole-based Schiff bases **5a**–**f**, **6a**–**f**, and **7a**–**f.**

Pyrazole-Based Schiff Bases	Anti-Inflammatory	
	Inhibition (%)	
	Protein Denaturation	Proteinase
**5a** *	26.83 ± 0.03	23.33 ± 0.03
**5b**	7.83 ± 0.01	6.81 ± 0.01
**5c**	15.41 ± 0.02	13.40 ± 0.02
**5d** *	27.79 ± 0.03	24.17 ± 0.03
**5e** *	27.33 ± 0.03	23.76 ± 0.03
**5f** *	27.28 ± 0.03	23.72 ± 0.03
**6a**	15.33 ± 0.02	13.33 ± 0.02
**6b**	17.94 ± 0.02	15.60 ± 0.02
**6c**	17.97 ± 0.02	15.62 ± 0.02
**6d**	15.62 ± 0.02	13.58 ± 0.02
**6e**	15.67 ± 0.02	13.63 ± 0.02
**6f**	15.64 ± 0.02	13.60 ± 0.02
**7a** *	26.87 ± 0.03	23.37 ± 0.03
**7b**	15.36 ± 0.02	13.35 ± 0.02
**7c**	15.38 ± 0.02	13.38 ± 0.02
**7d**	15.59 ± 0.02	13.56 ± 0.02
**7e**	8.05 ± 0.01	4.81 ± 0.01
**7f** *	27.75 ± 0.03	24.13 ± 0.03
**STD**	**Diclofenac Sodium**	
	49.08 ± 0.01	46.11 ± 0.02

Data calculated from three replicates and presented as the mean ± SE. * Indicates the most potent pyrazole-based Schiff bases.

**Table 3:** The cytotoxic activities (IC_50_ μM) and therapeutic indexes (TIs) of pyrazole-based Schiff bases **5a**, **5d**, **5e**, **5f**, **7a**, **7f**, and doxorubicin against lung (A549), colon (Caco-2), and normal lung (WI-38) cell lines.

Pyrazole-Based Schiff Bases	Lung (A549)	Colon (Caco-2)	Normal Lung (WI-38)	Lung (A549)	Colon (Caco-2)
	(IC_50_ μM)			Therapeutic Index (TI)	
**5a**	68.84 ± 0.14	60.29 ± 0.14	441.69 ± 8.80	6.41	7.32
**5d**	48.61 ± 0.14	62.33 ± 0.14	731.72 ± 10.46	14.83	11.73
**5e**	47.74 ± 0.20	40.99 ± 0.20	648.12 ± 7.57	13.57	15.81
**5f**	60.45 ± 0.12	61.98 ± 0.12	493.07 ± 9.88	8.15	7.95
**7a**	49.40 ± 0.18	42.42 ± 0.18	736.26 ± 7.95	14.90	17.35
**7f**	55.74 ± 0.24	49.01 ± 0.24	542.51 ± 8.52	9.73	11.06
**Doxorubicin**	36.45 ± 0.16	54.94 ± 0.16	304.94 ± 4.72	8.36	5.55

Data calculated from three replicates and presented as the mean ± SE.

**Table 4:** The enzymatic assay values after the treatments of lung (A549) and colon (Caco-2) cells with pyrazole-based Schiff bases **5a**, **5d**, **5e**, **5f**, **7a**, **7f**, and doxorubicin.

Pyrazole-Based Schiff Bases	Lung (A549)		Colon (Caco-2)	
	Caspase-3 (pg/mL)	Bcl-2 (ng/mL)	Caspase-3 (pg/mL)	Bcl-2 (ng/mL)
**DMSO**	85.92 ± 0.12	9.83 ± 0.05	97.06 ± 0.14	7.99 ± 0.08
**5a**	184.74 ± 0.26	6.55 ± 0.03	208.68 ± 0.31	5.33 ± 0.05
**5d**	300.73 ± 0.42	2.81 ± 0.01	242.65 ± 0.36	3.20 ± 0.03
**5e**	214.81 ± 0.30	3.93 ± 0.02	315.45 ± 0.46	2.46 ± 0.02
**5f**	236.29 ± 0.33	3.57 ± 0.02	266.92 ± 0.39	2.91 ± 0.03
**7a**	322.21 ± 0.45	2.62 ± 0.01	363.98 ± 0.54	2.13 ± 0.02
**7f**	244.88 ± 0.34	3.45 ± 0.02	276.62 ± 0.41	2.80 ± 0.03
**Doxorubicin**	330.80 ± 0.46	2.55 ± 0.01	373.68 ± 0.55	2.08 ± 0.02

Data calculated from three replicates and presented as the mean ± SE.

**Table 5:** ADMT properties of pyrazole-based Schiff bases (**5a**, **5d**, **5e**, **5f**, **7a**, and **7f**) using the pkCSM website.

Properties	5a	5d	5e	5f	7a	7f	The Ideal Values
**Absorption**							
**Intestinal absorption** **(human)**	91.357	95.510	96.003	96.711	93.723	93.474	Less than 30% is poorly absorbed
**Skin permeability** **(log Kp)**	−2.735	−2.735	−2.735	−2.735	−2.735	−2.735	>−2.5 is low
**Distribution**							
**BBB permeability**	−1.318	−1.530	−1.550	−1.704	−1.433	−1.818	Poorly is <−1, high is >0.3
**CNS permeability**	−1.705	−1.886	−1.817	−1.776	−2.061	−2.133	Penetrate is >−2, unable is <−3
**Metabolism**							
**CYP1A2 inhibitor**	Yes	Yes	No	No	Yes	No	No
**CYP2C19 inhibitor**	Yes	Yes	Yes	Yes	Yes	Yes	No
**CYP2C9 inhibitor**	Yes	Yes	Yes	Yes	Yes	Yes	No
**CYP2D6 inhibitor**	No	No	No	No	No	No	No
**CYP3A4 inhibitor**	Yes	Yes	Yes	Yes	Yes	Yes	No
**Toxicity**							
**AMES toxicity**	No	No	No	No	No	Yes	No
**Skin sensitization**	No	No	No	No	No	No	No

## Data Availability

Data are contained within the article.

## Supplementary Materials

The following supporting information can be downloaded at: https://www.mdpi.com/article/10.3390/ph17050655/s1, Table S1: The anti-diabetic activity of pyrazole-based Schiff bases **5a**–**f**, **6a**–**f**, and **7a**–**f**; Table S2: The anti-Alzheimer activity of pyrazole-based Schiff bases **5a**–**f**, **6a**–**f**, and **7a**–**f**; Figure S1: The anti-inflammatory activity of pyrazole-based Schiff bases **5a**–**f**, **6a**–**f**, **7a**–**f**, and diclofenac sodium as a standard drug; Table S3: Cytotoxic activity of the six pyrazole-based Schiff bases **5a**, **5d**, **5e**, **5f**, **7a**, and **7f** against human lung cancer (A549) cell line compared to Doxorubicin as a standard drug; Table S4: Cytotoxic activity of the six pyrazole-based Schiff bases **5a**, **5d**, **5e**, **5f**, **7a**, and **7f** against human colon cancer (Caco-2) cell line compared to Doxorubicin as a standard drug; Table S5: Cytotoxic activity of the six pyrazole-based Schiff bases **5a**, **5d**, **5e**, **5f**, **7a**, and **7f** against normal lung (WI-38) cell line compared to Doxorubicin as a standard drug.
