# Supplementary material for: Exploring the Potential Biological Activities of Pyrazole-Based Schiff Bases as Anti-Diabetic, Anti-Alzheimer’s, Anti-Inflammatory, and Cytotoxic Agents: In Vitro Studies with Computational Predictions
Source: Pharmaceuticals (Basel). 2024 May 17;17(5):655. doi: 10.3390/ph17050655 (PMC11125359; doi:10.3390/ph17050655)
Supplement: Supplementary file 1 [file pharmaceuticals-17-00655-s001.zip › pharmaceuticals-3001165-supplementary.pdf]

Supplemental File

For

Exploring the Potential Biological Activities of Pyrazole-Based Schiff Bases as Anti-Diabetic, Anti-Alzheimer's, Anti-Inflammatory, and Cytotoxic Agents: *In Vitro* Studies with Computational Predictions

Ahmed M. Naglah [1,*], Abdulrahman A. Almehizia [1], Asma S. Al-Wasidi [2], Amirah Senaitan Alharbi [3], Mohammed H. Alqarni [4], Ashraf S. Hassan [5,*] and Wael M. Aboulthana [6]

[1] Drug Exploration and Development Chair (DEDC), Department of Pharmaceutical Chemistry, College of Pharmacy, King Saud University, P.O. Box 2457, Riyadh 11451, Saudi Arabia; anaglah@ksu.edu.sa (A.M.N.), mehizia@ksu.edu.sa (A.A.A.)
[2] Department of Chemistry, College of Science, Princess Nourah Bint Abdulrahman University, Riyadh 11671, Saudi Arabia; asalwasidi@pnu.edu.sa (A.S.A.)
[3] King Khalid Hospital, King Saud University Medical City, P.O. Box 7805, Riyadh 11472, Saudi Arabia; Aalharbi1@ksu.edu.sa (A.S.A.)
[4] Department of Pharmacognosy, College of Pharmacy, Prince Sattam Bin Abdulaziz University, P.O. Box 173, Al-Kharj 11942, Saudi Arabia; m.alqarni@psau.edu.sa (M.H.A.)
[5] Organometallic and Organometalloid Chemistry Department, National Research Centre, Dokki 12622, Cairo, Egypt; as.el-salmoon@nrc.sci.eg (A.S.H.)
[6] Biochemistry Department, Biotechnology Research Institute, National Research Centre, Dokki 12622, Cairo, Egypt; wmkamel83@hotmail.com (W.M.A.)
* Correspondence: anaglah@ksu.edu.sa (A.M.N.); as.el-salmoon@nrc.sci.eg (A.S.H.)

Table S1. The anti-diabetic activity of pyrazole-based Schiff bases **5a-f, 6a-f,** and **7a-f.**

**Pyrazole-based Schiff bases**	**Anti-diabetic Inhibition (%)**	
	**α-Amylase**	**α-Glucosidase**
**5a***	31.24 ± 0.05	26.87 ± 0.05
**5b**	9.12 ± 0.01	7.85 ± 0.01
**5c**	17.94 ± 0.03	15.43 ± 0.03
**5d***	32.37 ± 0.05	27.84 ± 0.05
**5e***	31.83 ± 0.05	27.37 ± 0.05
**5f***	31.77 ± 0.05	27.33 ± 0.05
**6a**	17.85 ± 0.03	15.35 ± 0.03
**6b**	20.89 ± 0.03	17.96 ± 0.03
**6c**	20.92 ± 0.03	17.99 ± 0.03
**6d**	18.19 ± 0.03	15.64 ± 0.03
**6e**	18.25 ± 0.03	15.69 ± 0.03
**6f**	18.22 ± 0.03	15.67 ± 0.03
**7a***	31.30 ± 0.05	26.91 ± 0.05
**7b**	17.88 ± 0.03	15.38 ± 0.03
**7c**	17.91 ± 0.03	15.41 ± 0.03
**7d**	18.16 ± 0.03	15.61 ± 0.03
**7e**	9.38 ± 0.02	8.07 ± 0.02
**7f***	32.31 ± 0.05	27.79 ± 0.05
**STD**	**Acarbose**	
	76.58 ± 0.01	53.94 ± 0.01

Data calculated from three replicates and presented as mean ± SE.
*Indicated the most potent pyrazole-based Schiff bases

Table S2. The anti-Alzheimer activity of pyrazole-based Schiff bases **5a-f, 6a-f,** and **7a-f.**

Pyrazole-based Schiff bases	Anti-Alzheimer
	Inhibition (%)
	Acetylcholinesterase
**5a***	42.05 ± 0.03
**5b**	12.28 ± 0.01
**5c**	24.15 ± 0.01
**5d***	62.11 ± 0.04
**5e***	42.83 ± 0.03
**5f***	42.76 ± 0.03
**6a**	24.03 ± 0.01
**6b**	28.11 ± 0.02
**6c**	28.16 ± 0.02
**6d**	24.48 ± 0.01
**6e**	24.56 ± 0.01
**6f**	24.52 ± 0.01
**7a***	42.12 ± 0.03
**7b**	24.07 ± 0.01
**7c**	24.11 ± 0.01
**7d**	24.44 ± 0.01
**7e**	12.62 ± 0.01
**7f***	62.00 ± 0.04
**STD**	**Donepezil**
	70.32 ± 0.04

Data calculated from three replicates and presented as mean ± SE.
*Indicated the most potent pyrazole-based Schiff bases

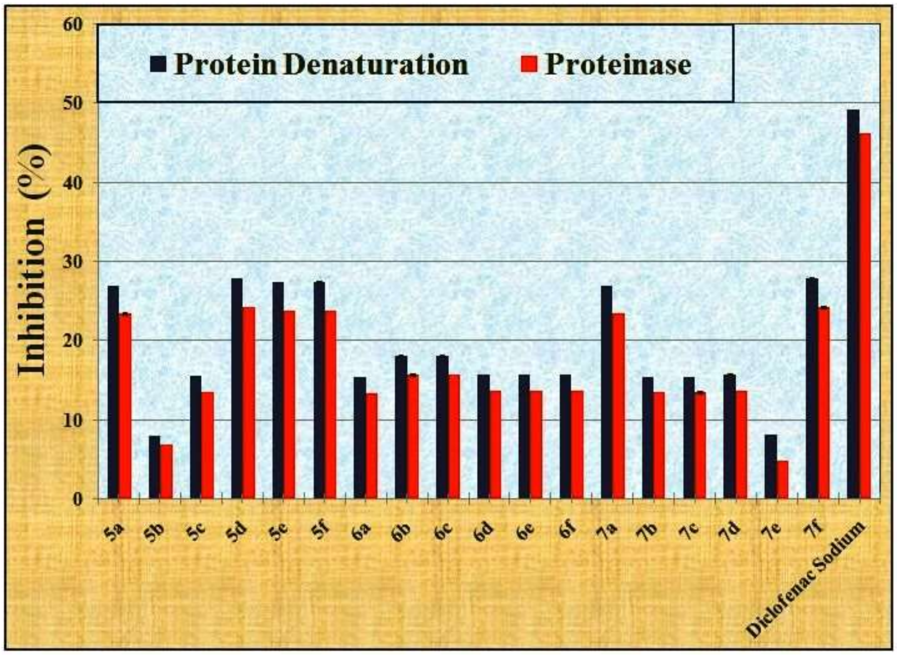

Figure S1. The anti-inflammatory activity of pyrazole-based Schiff bases **5a-f, 6a-f, 7a-f,** and diclofenac sodium as a standard drug

Table S3: Cytotoxic activity of the six pyrazole-based Schiff bases **5a**, **5d**, **5e**, **5f**, **7a**, and **7f** against human lung cancer (A549) cell line compared to Doxorubicin as a standard drug.

**5a**						
**Conc.**	**0.00**	**13.45**	**26.91**	**53.81**	**107.63**	**215.26**
Mean OD	0.34	0.29	0.26	0.18	0.11	0.07
Viability %	100.00	97.46	87.31	60.91	46.48	20.20
**IC_{50} (μM)**	**68.84 ± 0.14**					
**5d**						
**Conc.**	**0.00**	**12.64**	**25.27**	**50.55**	**101.09**	**202.19**
Mean OD	0.40	0.34	0.29	0.20	0.14	0.02
Viability %	100.00	90.46	79.41	48.01	36.58	18.10
**IC_{50} (μM)**	**48.61 ± 0.14**					
**5e**						
**Conc.**	**0.00**	**12.29**	**24.58**	**49.15**	**98.31**	**196.61**
Mean OD	0.35	0.29	0.24	0.15	0.09	0.08
Viability %	100.00	90.96	79.91	48.51	37.08	18.60
**IC_{50} (μM)**	**47.74 ± 0.20**					
**5f**						
**Conc.**	**0.00**	**11.81**	**23.63**	**47.26**	**94.51**	**189.03**
Mean OD	0.31	0.26	0.23	0.15	0.08	0.05
Viability %	100.00	97.46	87.31	60.91	46.48	20.20
**IC_{50} (μM)**	**60.45 ± 0.12**					
**7a**						
**Conc.**	**0.00**	**12.72**	**25.43**	**50.86**	**101.72**	**203.44**
Mean OD	0.46	0.40	0.35	0.26	0.20	0.08
Viability %	100.00	90.96	79.91	48.51	37.08	18.60
**IC_{50} (μM)**	**49.40 ± 0.18**					
**7f**						
**Conc.**	**0.00**	**11.24**	**22.48**	**44.96**	**89.93**	**179.85**
Mean OD	0.33	0.28	0.25	0.17	0.10	0.06
Viability %	100.00	96.21	86.06	59.66	45.23	18.95
**IC_{50} (μM)**	**55.74 ± 0.24**					
**DOX**						
**Conc.**	**0.00**	**11.50**	**23.00**	**46.00**	**91.99**	**183.99**
Mean OD	0.26	0.21	0.16	0.12	0.09	0.75
Viability %	100.00	80.86	69.31	36.91	18.92	11.20
**IC_{50} (μM)**	**36.45 ± 0.16**					

Table S4: Cytotoxic activity of the six pyrazole-based Schiff bases **5a**, **5d**, **5e**, **5f**, **7a**, and **7f** against human colon cancer (Caco-2) cell line compared to Doxorubicin as a standard drug.

**5a**						
**Conc.**	**0.00**	**13.45**	**26.91**	**53.81**	**107.63**	**215.26**
Mean OD	0.30	0.23	0.18	0.11	0.08	0.08
Viability %	100.00	82.93	74.41	55.38	33.22	25.84
**IC_{50} (µM)**	**60.29 ± 0.14**					
**5d**						
**Conc.**	**0.00**	**12.64**	**25.27**	**50.55**	**101.09**	**202.19**
Mean OD	0.40	0.28	0.21	0.12	0.09	0.07
Viability %	100.00	70.71	51.15	40.54	26.77	23.43
**IC_{50} (µM)**	**62.33 ± 0.14**					
**5e**						
**Conc.**	**0.00**	**12.29**	**24.58**	**49.15**	**98.31**	**196.61**
Mean OD	0.33	0.20	0.14	0.04	0.01	0.05
Viability %	100.00	70.21	50.65	40.04	26.27	11.43
**IC_{50} (µM)**	**40.99 ± 0.20**					
**5f**						
**Conc.**	**0.00**	**11.81**	**23.63**	**47.26**	**94.51**	**189.03**
Mean OD	0.29	0.22	0.17	0.10	0.07	0.07
Viability %	100.00	89.43	80.91	61.88	39.72	32.34
**IC_{50} (µM)**	**61.98 ± 0.12**					
**7a**						
**Conc.**	**0.00**	**12.72**	**25.43**	**50.86**	**101.72**	**203.44**
Mean OD	0.39	0.26	0.20	0.10	0.07	0.05
Viability %	100.00	70.21	50.65	40.04	26.27	22.93
**IC_{50} (µM)**	**42.42 ± 0.18**					
**7f**						
**Conc.**	**0.00**	**11.24**	**22.48**	**44.96**	**89.93**	**179.85**
Mean OD	0.29	0.22	0.17	0.10	0.07	0.07
Viability %	100.00	81.68	73.16	54.13	31.97	24.59
**IC_{50} (µM)**	**49.01 ± 0.24**					
**DOX**						
**Conc.**	**0.00**	**11.50**	**23.00**	**46.00**	**91.99**	**183.99**
Mean OD	0.36	0.31	0.22	0.14	0.16	0.03
Viability %	100.00	72.32	50.77	41.86	23.02	20.05
**IC_{50} (µM)**	**54.94 ± 0.16**					

Table S5: Cytotoxic activity of the six pyrazole-based Schiff bases **5a**, **5d**, **5e**, **5f**, **7a**, and **7f** against normal lung (WI-38) cell line compared to Doxorubicin as a standard drug.

**5a**						
**Conc.**	**0.00**	**13.45**	**26.91**	**53.81**	**107.63**	**215.26**
Mean OD	0.41	0.38	0.36	0.29	0.20	0.16
Viability %	100.00	81.40	75.47	69.54	54.19	38.28
**IC_{50} (μM)**	**441.69 ± 8.80**					
**5d**						
**Conc.**	**0.00**	**12.64**	**25.27**	**50.55**	**101.09**	**202.19**
Mean OD	0.29	0.27	0.26	0.25	0.21	0.17
Viability %	100.00	98.66	90.70	82.73	76.75	62.69
**IC_{50} (μM)**	**731.72 ± 10.46**					
**5e**						
**Conc.**	**0.00**	**12.29**	**24.58**	**49.15**	**98.31**	**196.61**
Mean OD	0.35	0.32	0.30	0.23	0.14	0.16
Viability %	100.00	92.90	86.97	81.04	65.69	38.28
**IC_{50} (μM)**	**648.12 ± 7.57**					
**5f**						
**Conc.**	**0.00**	**11.81**	**23.63**	**47.26**	**94.51**	**189.03**
MeanOD	0.40	0.37	0.35	0.28	0.19	0.15
Viability %	100.00	87.90	81.97	76.04	60.69	44.78
**IC_{50} (μM)**	**493.07 ± 9.88**					
**7a**						
**Conc.**	**0.00**	**12.72**	**25.43**	**50.86**	**101.72**	**203.44**
Mean OD	0.35	0.33	0.32	0.31	0.27	0.23
Viability %	100.00	98.66	90.70	82.73	76.75	62.69
**IC_{50} (μM)**	**736.26 ± 7.95**					
**7f**						
**Conc.**	**0.00**	**11.24**	**22.48**	**44.96**	**89.93**	**179.85**
Mean OD	0.34	0.32	0.31	0.30	0.26	0.22
Viability %	100.00	95.21	87.25	79.28	73.30	59.24
**IC_{50} (μM)**	**542.51 ± 8.52**					
**DOX**						
**Conc.**	**0.00**	**11.50**	**23.00**	**46.00**	**91.99**	**183.99**
Mean OD	0.36	0.34	0.31	0.28	0.19	0.18
Viability %	100.00	77.45	69.12	62.29	52.69	38.49
**IC_{50} (μM)**	**304.94 ± 4.72**					

Pyrazole-based Schiff bases 5a-f, 6a-f, and 7a-f

General method for synthesis of pyrazole-based Schiff bases 5a-f, 6a-f, and 7a-f

A mixture of 5-aminopyrazoles **1a–f** (0.01mol) and the appropriate hetero-aromatic aldehydes (0.01mol) [namely; 4-(piperidin-1-yl) benzaldehyde (**2**), 5-chloro-3-methyl-1-phenyl-1*H*-pyrazole-4-carbaldehyde (**3**), and 4-antipyrinecarboxaldehyde (**4**)] with a catalytic amount of glacial acetic acid (1ml) in absolute ethanol (25ml) was refluxed for one hour and then left to cool. The solid product was filtered off, dried and finally recrystallized from ethanol to afford the corresponding pyrazole-based Schiff bases **5a-f, 6a-f,** and **7a-f**.

***5-(4-(Piperidin-1-yl)benzylideneamino)-N-phenyl-3-(phenylamino)-1H-pyrazole-4-carboxamide* (5a)**

Yield: 71 %. ^{1}H NMR (DMSO-d_6, 500 MHz, δ ppm): 1.53-1.59 (m, 6H, 3CH_2, piperidine moiety), 3.43 (t, 4H, 2CH_2, piperidine moiety), 6.87 (s, 1H, ArH), 7.09 (t, 3H, ArH), 7.29 (t, 2H, ArH), 7.38 (t, 2H, ArH), 7.60 (s, 2H, ArH), 7.68 (d, 2H, J = 7.7 Hz, ArH), 7.84 (d, 2H, J = 8.6 Hz, ArH), 8.76 (s, 1H, -CH=N-), 8.96 (s, 1H, NH), 10.14 (s, 1H, NH), 12.73 (s, 1H, NH). ^{13}CNMR (DMSO-d_6, 125 MHz, δ ppm): 23.94, 24.95, 47.62 (5C, piperidine moiety), 92.66, 113.71, 116.27, 118.92, 122.17, 123.21, 128.99, 129.13, 131.45, 138.59, 141.67, 147.73, 150.64, 152.71, 154.01, 162.59 (22C), 163.07 (C, C=O). MS (*m/z*, %): 464.35 (M^+, 12.27), 264.21 (100).

***5-(4-(Piperidin-1-yl)benzylideneamino)-3-(phenylamino)-N-(4-methylphenyl)-1H-pyrazole-4-carboxamide* (5b)**

Yield: 77 %. ^{1}H NMR (DMSO-d_6, 500 MHz, δ ppm): 1.52-1.57 (m, 6H, 3CH_2, piperidine moiety), 2.27 (s, 3H, CH_3), 3.40 (t, 4H, 2CH_2, piperidine moiety), 6.87 (t, 1H, ArH), 7.05 (d, 2H, J = 8.8 Hz, ArH), 7.17 (d, 2H, J = 8.2 Hz, ArH), 7.28 (t, 2H, ArH), 7.57 (d, 4H, J = 8.3 Hz, ArH), 7.81 (d, 2H, J = 8.6 Hz, ArH), 8.75 (s, 1H, -CH=N-), 8.99 (s, 1H, NH), 10.10 (s, 1H, NH), 12.70 (s, 1H, NH). ^{13}C NMR (DMSO-d_6, 125 MHz, δ ppm): 20.42 (C, CH_3), 23.92, 24.93, 47.60 (5C, piperidine moiety), 92.75, 113.68, 116.37, 118.88, 122.74, 128.98, 129.49, 131.40, 132.13, 136.10, 137.79, 141.53, 150.62, 153.97, 157.92, 160.33 (22C), 162.91 (C, C=O). MS (*m/z*, %): 478.39 (M^+, 14.66), 77.02 (100).

***N-(4-Chlorophenyl)-3-(phenylamino)-5-((4-(piperidin-1-yl)benzylidene)amino)-1H-pyrazole-4-carboxamide* (5c)**

Yield: 68 %. ^{1}H NMR (DMSO-d_6, 500 MHz, δ ppm): 1.53-1.58 (m, 6H, 3CH_2, piperidine moiety), 3.73 (t, 4H, 2CH_2, piperidine moiety), 6.87 (s, 1H, ArH), 7.05 (d, 2H, J = 6.9 Hz, ArH), 7.28 (s, 2H, ArH), 7.40 (d, 2H, J = 7.3 Hz, ArH), 7.58 (s, 2H, ArH), 7.68 (d, 2H, J = 7.5 Hz, ArH), 7.81 (d, 2H, J = 6.9 Hz, ArH), 8.72 (s, 1H, -CH=N-), 8.88 (s, 1H, NH), 10.23 (s, 1H, NH), 12.72 (s, 1H, NH). ^{13}CNMR (DMSO-d_6, 125 MHz, δ ppm): 23.93, 24.95, 47.32 (5C, piperidine moiety), 92.72, 113.68, 116.36, 117.23, 120.49, 122.69, 126.72, 127.30, 128.95, 131.51, 137.53, 140.07, 151.34, 152.68, 154.06, 158.72 (22C), 163.03 (C, C=O).

***3-(p-Methoxyphenylamino)-N-phenyl-5-(4-(piperidin-1-yl)benzylideneamino)-1H-pyrazole-4-carboxamide* (5d)**

Yield: 87 %. ^{1}HNMR (300 MHz)δ ppm: 1.60 (s, 6H, piperidine moiety), 3.43 (s, 4H, piperidine moiety), 3.72 (s, 3H, OCH_3), 6.89 (d, 2H, J = 8.1 Hz, ArH), 7.08 (t, 3H, ArH), 7.35-7.48 (m, 4H, ArH), 7.68 (d, 2H, J = 8.2 Hz, ArH), 7.85 (d, 2H, J = 8.3 Hz, ArH), 8.71 (s, 1H, -CH=N-), 8.77, 10.14, 12.56 (3s, 3H, 3NH, exchangeable

with D_2O). ^{13}C-NMR (76 MHz) δ ppm: 23.95, 24.93, 47.65 (5C, piperidine moiety), 55.20 (-OCH_3), 92.57, 113.73, 114.30, 117.38, 118.81, 123.10, 125.43, 129.11, 131.36, 135.13, 138.68, 151.93, 153.89, 155.91, 160.09 (22C), 163.06 (C=O).

***3-(p-Methoxyphenylamino)-5-(4-(piperidin-1-yl)benzylideneamino)-N-p-tolyl-1H-pyrazole-4-carboxamide* (5e)**

Yield: 82 %. ^{1}H-NMR (300 MHz) δ ppm: 1.62 (s, 6H, piperidine moiety), 2.28 (s, 3H, CH_3), 3.46 (s, 4H, piperidine moiety), 3.72 (s, 3H, OCH_3), 6.88 (d, 2H, J = 8.6 Hz, ArH), 7.11 (d, 2H, J = 8.9 Hz, ArH), 7.19 (d, 2H, J = 8.1 Hz, ArH), 7.47-7.59 (m, 4H, ArH), 7.85 (d, 2H, J = 8.9 Hz, ArH), 8.71 (s, 1H, -CH=N-), 8.77, 10.06 (2s, 2H, 2NH, exchangeable with D_2O), 12.56 (s, 1H, NH). Anal. Calcd. (%) for $C_{30}H_{32}N_6O_2$ (508.61): C, 70.84; H, 6.34; N, 16.52. Found: C, 70.90; H, 6.25; N, 16.43.

***N-(p-Chlorophenyl)-3-(p-methoxyphenylamino)-5-(4-(piperidin-1-yl)benzylideneamino)-1H-pyrazole-4-carboxamide* (5f)**

Yield: 81 %. ^{1}H-NMR (300 MHz) δ ppm: 1.62 (s, 6H, piperidine moiety), 3.45 (s, 4H, piperidine moiety), 3.72 (s, 3H, OCH_3), 6.89 (d, 2H, J = 9.0 Hz, ArH), 7.11 (d, 2H, J = 9.2 Hz, ArH), 7.42 (d, 2H, J = 8.9 Hz, ArH), 7.46 (d, 2H, J = 9.3 Hz, ArH), 7.70 (d, 2H, J = 8.9 Hz, ArH), 7.85 (d, 2H, J = 8.9 Hz, ArH), 8.64 (s, 1H, -CH=N-), 8.80, 10.24 (2s, 2H, 2NH, exchangeable with D_2O), 12.30 (s, 1H, NH).

***5-((5-Chloro-3-methyl-1-phenyl-1H-pyrazol-4-yl)methyleneamino)-N-phenyl-3-(phenylamino)-1H-pyrazole-4-carboxamide* (6a)**

Yield: 78 %. ^{1}H NMR (DMSO-d_6, 500 MHz, δ ppm): 2.57 (s, 3H, CH_3), 6.88 (t, 1H, ArH), 7.09 (t, 1H, ArH), 7.28 (t, 2H, ArH), 7.35 (t, 2H, ArH), 7.53-7.64 (m, 9H, ArH), 8.93 (s, 1H, -CH=N-), 9.01 (s, 1H, NH), 9.57 (s, 1H, NH), 12.52 (s, 1H, NH). ^{13}CNMR (DMSO-d_6, 125 MHz, δ ppm): 13.99 (C, CH_3), 93.13, 114.48, 116.58, 120.51, 123.66, 125.22, 128.86, 129.00, 129.21, 129.37, 130.47, 130.51, 136.83, 137.90, 141.02, 144.16, 150.66, 154.49, 158.05 (25C), 162.83 (C, C=O).

***5-((5-Chloro-3-methyl-1-phenyl-1H-pyrazol-4-yl)methyleneamino)-3-(phenylamino)-N-(4-methylphenyl)-1H-pyrazole-4-carboxamide* (6b)**

Yield: 80 %. ^{1}H NMR (DMSO-d_6, 500 MHz, δ ppm): 2.25 (s, 3H, CH_3), 2.54 (s, 3H, CH_3), 6.84 (s, 1H, ArH), 7.13 (d, 2H, J = 7.9 Hz, ArH), 7.27 (s, 2H, ArH), 7.45 (d, 2H, J = 8.0 Hz, ArH), 7.53-7.63 (m, 7H, ArH), 8.87 (s, 1H, -CH=N-), 9.03 (s, 1H, NH), 9.48 (s, 1H, NH), 12.94 (s, 1H, NH). ^{13}C NMR (DMSO-d_6, 125 MHz, δ ppm): 14.01, 20.42 (2C, 2CH_3), 93.52, 106.68, 114.48, 116.12, 120.59, 125.18, 128.96, 129.24, 129.35, 130.38, 132.67, 135.33, 136.82, 138.14, 142.20, 150.68, 154.88, 155.77, 161.53 (25C), 162.74 (C, C=O). MS (m/z, %): 511.28 (M+2, 10.50), 509.36 (M^+, 23.50), 76.95 (100).

***5-(((5-Chloro-3-methyl-1-phenyl-1H-pyrazol-4-yl)methylene)amino)-N-(4-chlorophenyl)-3-(phenylamino)-1H-pyrazole-4-carboxamide* (6c)**

Yield: 73 %. ^{1}H NMR (DMSO-d_6, 500 MHz, δ ppm): 2.54 (s, 3H, CH_3), 6.85 (s, 1H, ArH), 7.26 (s, 2H, ArH), 7.38 (d, 2H, J = 8.7 Hz, ArH), 7.53-7.63 (m, 9H, ArH), 8.87 (s, 1H, -CH=N-), 8.93 (s, 1H, NH), 9.56 (s, 1H, NH), 12.98 (s, 1H, NH). ^{13}C NMR (DMSO-d_6, 125 MHz, δ ppm): 14.02 (C, CH_3), 97.47, 114.50, 116.30, 122.08, 125.24, 127.34, 128.78, 129.27, 129.43, 130.50, 136.85, 136.91, 137.98, 140.35, 149.08, 152.20,

153.12, 160.06, 161.04 (25C), 162.86 (C, C=O). MS (*m/z*, %): 534.40 (M+4, 0.36), 533.40 (M+3, 1.41), 532.40 (M+2, 2.78), 531.40 (M+1, 8.72), 530.40 (M^+, 4.18), 529.40 (M-1, 11.50), 77.10 (100).

5-((5-Chloro-3-methyl-1-phenyl-1H-pyrazol-4-yl)methyleneamino)-3-(p-methoxyphenylamino)-N-phenyl-1H-pyrazole-4-carboxamide (**6d**).

Yield: 79 %. ^{1}H-NMR (300 MHz) δ ppm: 2.59 (s, 3H, CH_3), 3.73 (s, 3H, OCH_3), 6.90 (d, 2H, *J* = 7.5 Hz, ArH), 7.09 (t, 1H, ArH), 7.35 (t, 2H, ArH), 7.53-7.67 (m, 9H, ArH), 8.76 (s, 1H, -CH=N-), 8.91, 9.53, 12.87 (3s, 3H, 3NH, exchangeable with D_2O). MS (*m/z*, %): 525 (M^+, 23.62).

5-((5-Chloro-3-methyl-1-phenyl-1H-pyrazol-4-yl)methyleneamino)-3-(p-methoxyphenylamino)-N-p-tolyl-1H-pyrazole-4-carboxamide (**6e**).

Yield: 77 %. ^{1}H-NMR (300 MHz) δ ppm: 2.27 (s, 3H, CH_3), 2.58 (s, 3H, CH_3),3.72 (s, 3H, OCH_3), 6.89 (t, 1H, ArH), 7.16 (d, 2H, *J* = 8.3 Hz, ArH), 7.46 (d, 2H, *J* = 8.4 Hz, ArH), 7.52-7.67 (m, 8H, ArH), 8.78 (s, 1H, -CH=N-), 8.90, 9.46, 12.86 (3s, 3H, 3NH, exchangeable with D_2O). Anal. Calcd. (%) for $C_{29}H_{26}ClN_7O_2$ (540.02): C, 64.50; H, 4.85; N, 18.16. Found: C, 64.45; H, 4.90; N, 18.10.

5-((5-Chloro-3-methyl-1-phenyl-1H-pyrazol-4-yl)methyleneamino)-N-(p-chlorophenyl)-3-(p-methoxyphenylamino)-1H-pyrazole-4-carboxamide **(6f).**

Yield: 70%. ^{1}H-NMR (300 MHz) δ ppm: 2.58 (s, 3H, CH_3), 3.73 (s, 3H, OCH_3), 6.75 (t, 1H, ArH), 6.90 (d, 2H, *J* = 8.8 Hz, ArH), 7.34 (d, 2H, *J* = 9.0 Hz, ArH), 7.41 (t, 2H, ArH), 7.63-7.67 (m, 4H, ArH), 7.75 (d, 2H, *J* = 8.9 Hz, ArH), 8.69 (s, 1H, -CH=N-), 9.19, 10.26, 12.73 (3s, 3H, 3NH, exchangeable with D_2O). MS (*m/z*, %): 560 (M^+, 9.06). Anal. Calcd. (%) for $C_{28}H_{23}Cl_2N_7O_2$ (560.43): C, 60.01; H, 4.14; N, 17.49. Found: C, 59.95; H, 4.20; N, 17.44.

5-((2,3-Dimethyl-5-oxo-1-phenyl-2,5-dihydro-1H-pyrazol-4-yl)methyleneamino)-N-phenyl-3-(phenylamino)-1H-pyrazole-4-carboxamide (**7a**)

Yield: 82 %. ^{1}H NMR (DMSO-d_6, 500 MHz, δ ppm): 2.61 (s, 3H, CH_3), 3.36 (s, 3H, NCH_3), 6.86 (t, 1H, ArH), 7.02 (t, 1H, ArH), 7.26-7.29 (m, 4H, ArH), 7.42 (d, 2H, *J* = 7.3 Hz, ArH), 7.49 (t, 1H, ArH), 7.58 (t, 4H, ArH), 7.90 (d, 2H, *J* = 7.9 Hz, ArH), 8.79 (s, 1H, -CH=N-), 9.03 (s, 1H, NH), 10.50 (s, 1H, NH), 12.47 (s, 1H, NH). ^{13}C NMR (DMSO-d_6, 125 MHz, δ ppm): 10.97 (C, CH_3), 33.97 (C, NCH_3), 92.53, 101.97, 116.30, 119.67, 122.91, 127.25, 128.65, 128.68, 128.97, 129.01, 129.48, 133.64, 139.08, 141.41, 148.54, 154.04, 154.41, 154.57 (24C), 161.70, 163.46 (2C, 2C=O). MS (*m/z*, %): 491.33 (M^+, 11.78), 76.99 (100).

5-((2,3-Dimethyl-5-oxo-1-phenyl-2,5-dihydro-1H-pyrazol-4-yl)methyleneamino)-3-(phenylamino)-N-(4-methylphenyl)-1H-pyrazole-4-carboxamide **(7b)**

Yield: 84 %. ^{1}H NMR (DMSO-d_6, 500 MHz, δ ppm): 2.24 (s, 3H, CH_3), 2.60 (s, 3H, CH_3), 3.38 (s, 3H, NCH_3), 6.85 (t, 1H, ArH), 7.07 (d, 2H, *J* = 8.2 Hz, ArH), 7.28 (t, 2H, ArH), 7.41 (d, 2H, *J* = 7.6 Hz, ArH), 7.49 (t, 1H, ArH), 7.58 (t, 4H, ArH), 7.79 (d, 2H, *J* = 8.2 Hz, ArH), 8.79 (s, 1H, -CH=N-), 9.05 (s, 1H, NH), 10.43 (s, 1H, NH), 12.54 (s, 1H, NH). ^{13}C NMR (DMSO-d_6, 125 MHz, δ ppm): 10.97, 20.36 (2C, 2CH_3), 33.92 (C, NCH_3), 92.58, 101.97, 116.25, 116.37, 119.64, 127.15, 128.56, 128.93, 129.04, 129.43, 129.57, 131.77, 133.64, 136.53, 141.43, 152.17, 154.01, 154.47 (24C), 161.73, 163.26 (2C, 2C=O). MS (*m/z*, %): 505.36 (M^+, 15.49), 77.03 (100).

***N-(4-Chlorophenyl)-5-(((1,5-dimethyl-3-oxo-2-phenyl-2,3-dihydro-1H-pyrazol-4-yl)methylene)amino)-3-(phenylamino)-1H-pyrazole-4-carboxamide* (7c)**

Yield: 79 %. ^{1}H NMR (DMSO-d_6, 500 MHz, δ ppm): 2.57 (s, 3H, CH_3), 3.36 (s, 3H, NCH_3), 6.86 (t, 1H, ArH), 7.26-7.30 (m, 4H, ArH), 7.42 (d, 2H, J = 7.7 Hz, ArH), 7.49 (t, 1H, ArH), 7.58 (t, 4H, ArH), 7.97 (d, 2H, J = 8.6 Hz, ArH), 8.79 (s, 1H, -CH=N-), 8.95 (s, 1H, NH), 10.70 (s, 1H, NH), 12.63 (s, 1H, NH). ^{13}C NMR (DMSO-d_6, 125 MHz, δ ppm): 10.80 (C, CH_3), 34.01 (C, NCH_3), 92.51, 102.08, 116.33, 121.19, 126.43, 127.16, 128.47, 128.97, 129.48, 133.59, 134.06, 135.82, 136.60, 137.29, 138.13, 151.96, 153.93, 154.21 (24C), 161.46, 163.49 (2C, 2C=O). MS (*m/z*, %): 527 (M+2, 4.02), 525.17 (M^+, 10.05), 127.01 (100).

***5-((1,5-Dimethyl-3-oxo-2-phenyl-2,3-dihydro-1H-pyrazol-4-yl)methyleneamino)-3-(p-methoxyphenylamino)-N-phenyl-1H-pyrazole-4-carboxamide* (7d)**

Yield: 82 %. ^{1}H-NMR (400 MHz) δ ppm: 2.64 (s, 3H, CH_3), 3.39 (s, 3H, NCH_3),3.72 (s, 3H, OCH_3), 6.88 (d, 2H, J = 9.0 Hz, ArH), 7.01 (t, 1H, ArH), 7.27 (t, 2H, ArH), 7.42-7.52 (m, 5H, ArH), 7.60 (t, 2H, ArH), 7.88 (d, 2H, J = 7.6 Hz, ArH), 8.77 (s, 1H, -CH=N-), 8.79, 10.43, 12.44 (3s, 3H, 3NH, exchangeable with D_2O). ^{13}C-NMR (101 MHz) δ ppm: 11.53 (CH_3), 33.91 (NCH_3), 55.29 (OCH_3), 102.01, 105.94, 114.24, 121.47, 123.52, 125.27, 128.39, 129.35, 133.71, 133.84, 135.41, 144.64, 148.13, 153.60, 154.32, 156.81 (24C), 162.36 (C=O, amide), 166.71 (C=O, antipyrine). MS (*m/z*, %): 521 (M^+, 28.63).

***5-((1,5-Dimethyl-3-oxo-2-phenyl-2,3-dihydro-1H-pyrazol-4-yl)methyleneamino)-3-(p-methoxyphenylamino)-N-p-tolyl-1H-pyrazole-4-carboxamide* (7e)**

Yield: 87 %. ^{1}H-NMR (400 MHz) δ ppm: 2.24 (s, 3H, CH_3), 2.62 (s, 3H, CH_3), 3.38 (s, 3H, NCH_3),3.71 (s, 3H, OCH_3), 6.88 (d, 2H, J = 8.7 Hz, ArH), 7.07 (d, 2H, J = 8.0 Hz, ArH), 7.42 (d, 2H, J = 7.6 Hz, ArH), 7.47-7.61 (m, 5H, ArH), 7.76 (d, 2H, J = 8.1 Hz, ArH), 8.79 (s, 2H, -CH=N- & NH exchangeable with D_2O), 10.38, 12.23 (2s, 2H, 2NH, exchangeable with D_2O). ^{13}C-NMR (101 MHz) δ ppm: 11.56 (CH_3), 21.29 (CH_3), 33.87 (NCH_3), 55.31 (OCH_3), 102.04, 105.96, 114.19, 121.52, 123.49, 125.25, 128.37, 129.31, 133.73, 133.80, 135.39, 144.61, 148.11, 153.59, 154.34, 156.82 (24C), 162.34 (C=O, amide), 166.69 (C=O, antipyrine). MS (*m/z*, %): 535 (M^+, 35.07).

***N-(4-Chlorophenyl)-5-((1,5-dimethyl-3-oxo-2-phenyl-2,3-dihydro-1H-pyrazol-4-yl)methyleneamino)-3-(p-methoxyphenylamino)-1H-pyrazole-4-carboxamide* (7f)**

Yield: 82 %. ^{1}H-NMR (400 MHz) δ ppm: 2.61 (s, 3H, CH_3), 3.39 (s, 3H, N-CH_3),3.72 (s, 3H, OCH_3), 6.88 (d, 2H, J = 9.1 Hz, ArH), 7.30 (d, 2H, J = 8.9 Hz, ArH), 7.42-7.52 (m, 5H, ArH), 7.60 (t, 2H, ArH), 7.96 (d, 2H, J = 8.9 Hz, ArH), 8.71 (s, 1H, -CH=N-), 8.81, 10.66, 12.59 (3s, 3H, 3NH, exchangeable with D_2O). ^{13}C-NMR (101 MHz) δ ppm: 11.52 (CH_3), 33.86 (N-CH_3), 55.59 (OCH_3), 102.08, 105.92, 114.20, 121.58, 123.51, 125.26, 128.41, 129.28, 133.71, 133.78, 135.43, 144.60, 148.14, 153.61, 154.36, 156.88 (24C), 162.36 (C=O, amide), 166.72 (C=O, antipyrine). MS (*m/z*, %): 556 (M^+, 14.46)..

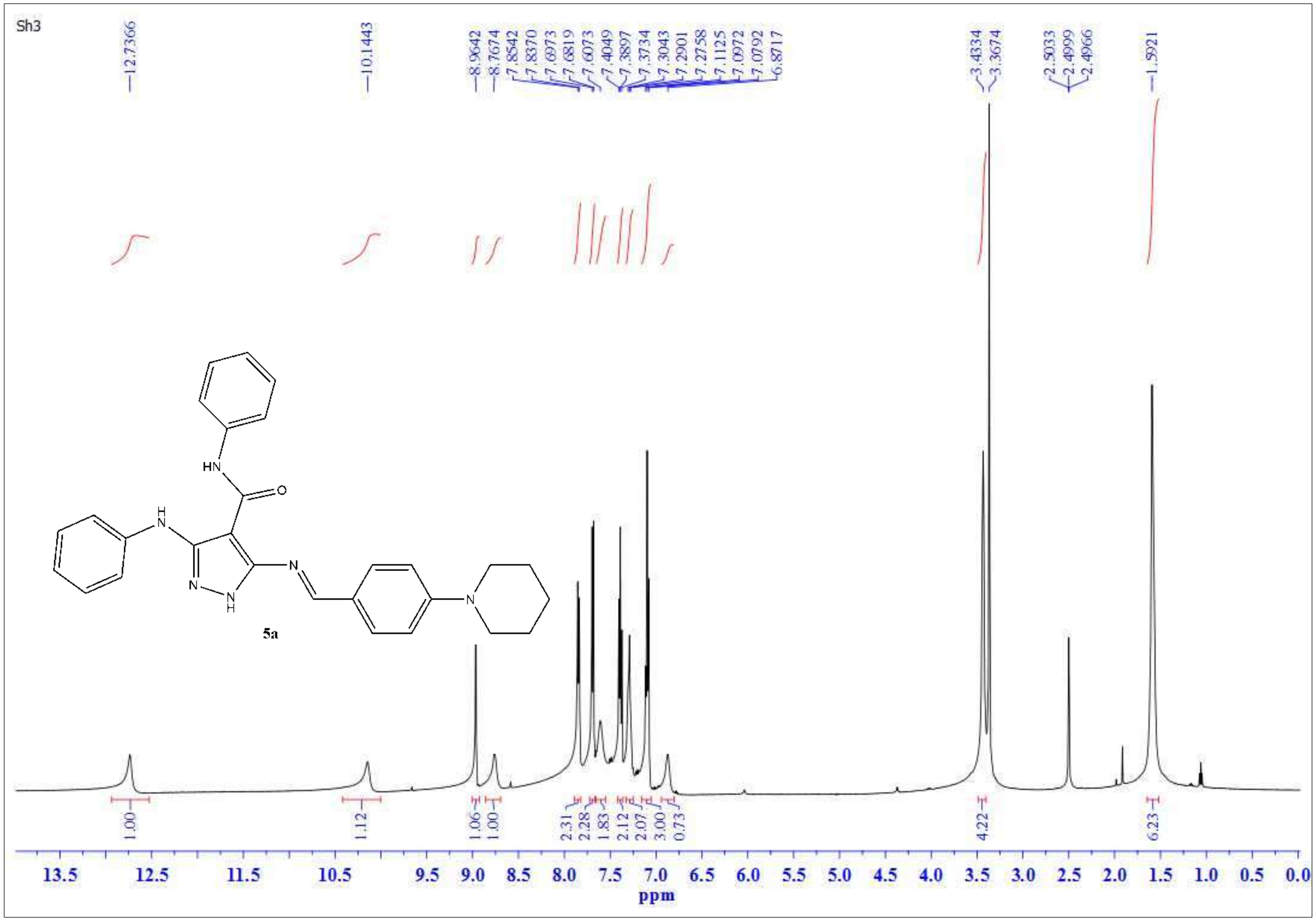

The ^{1}H NMR spectrum of Schiff base **5a**

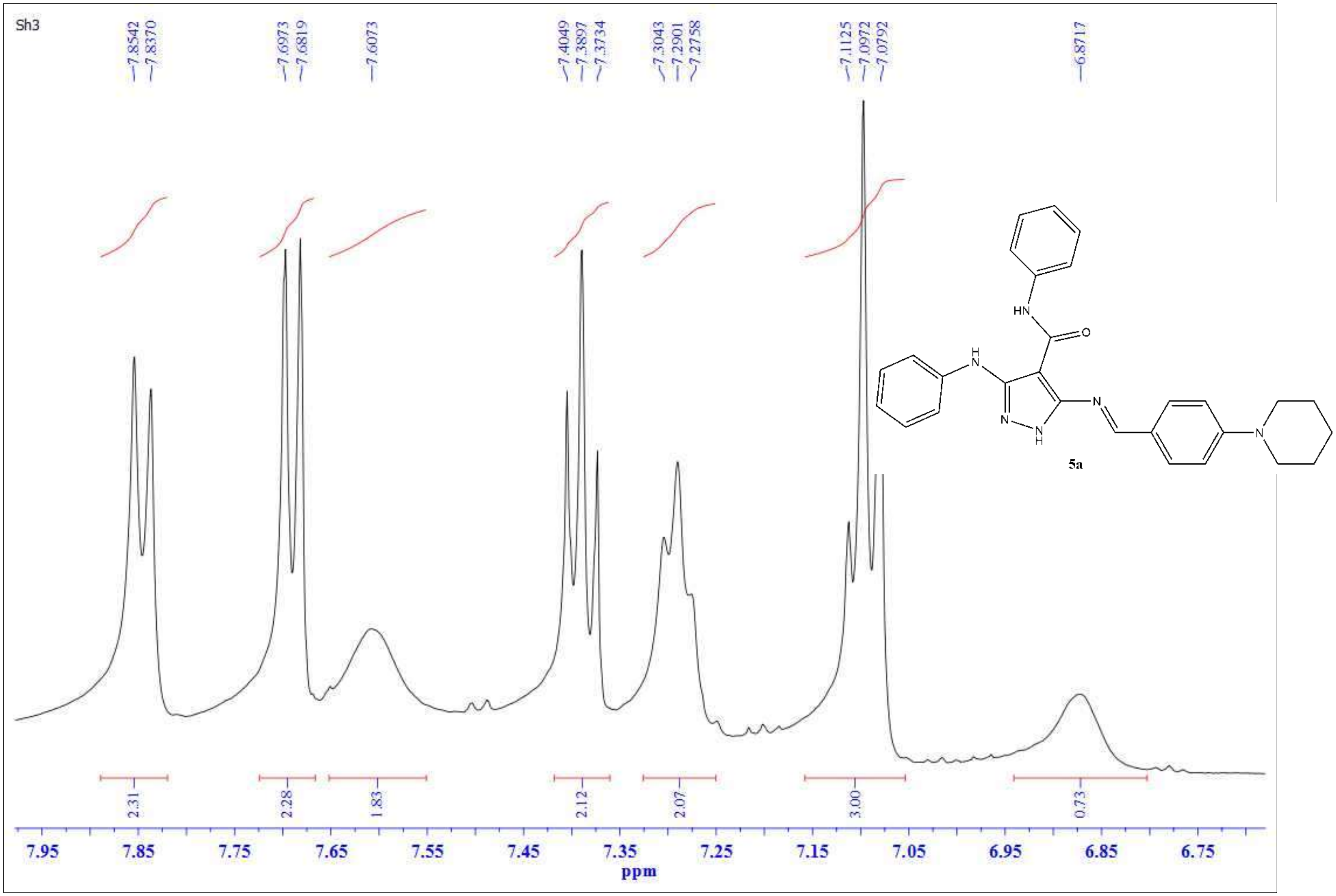

The ^{1}H NMR aromatic region spectrum of Schiff base **5a**

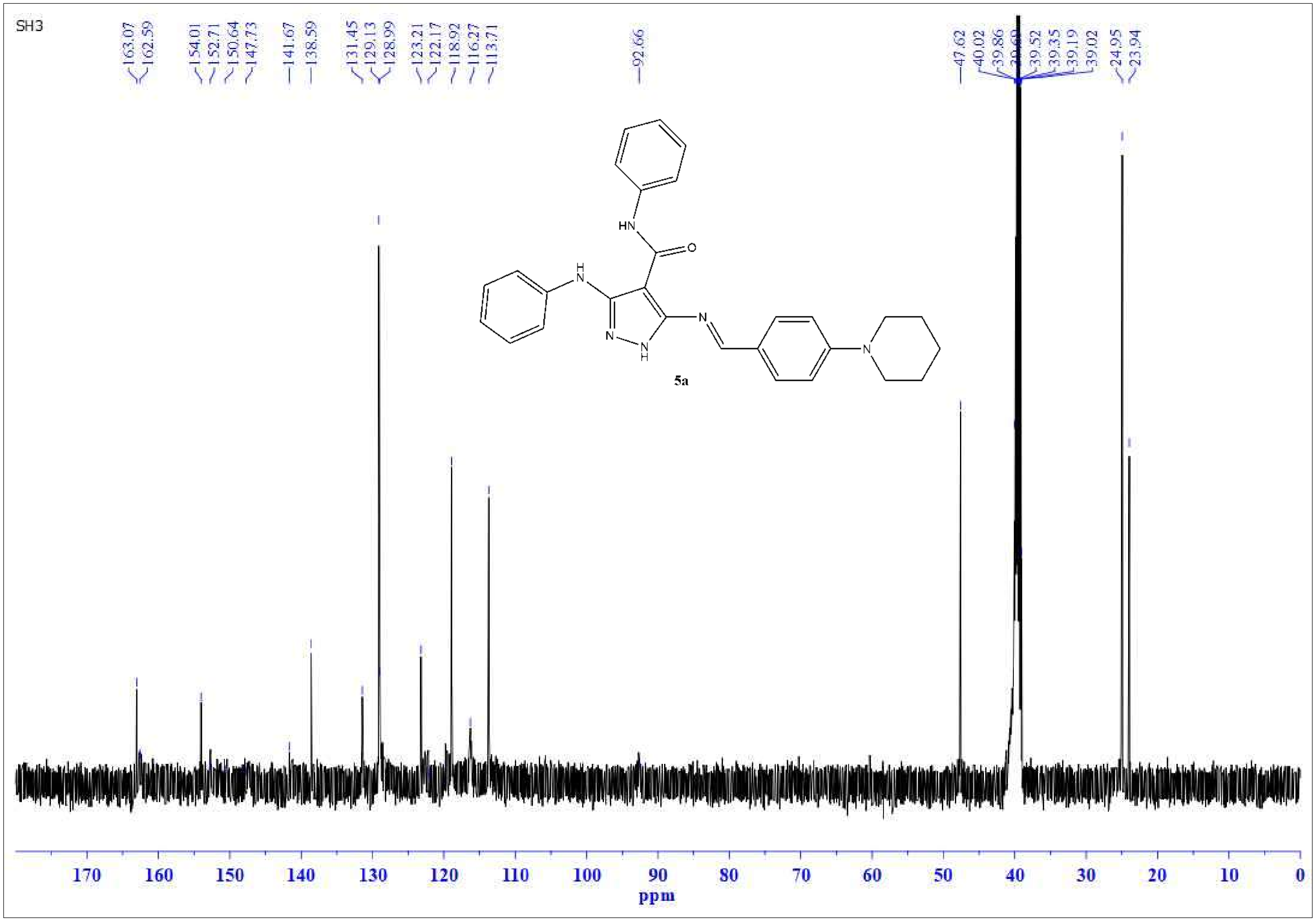

The ^{13}C NMR spectrum of Schiff base **5a**

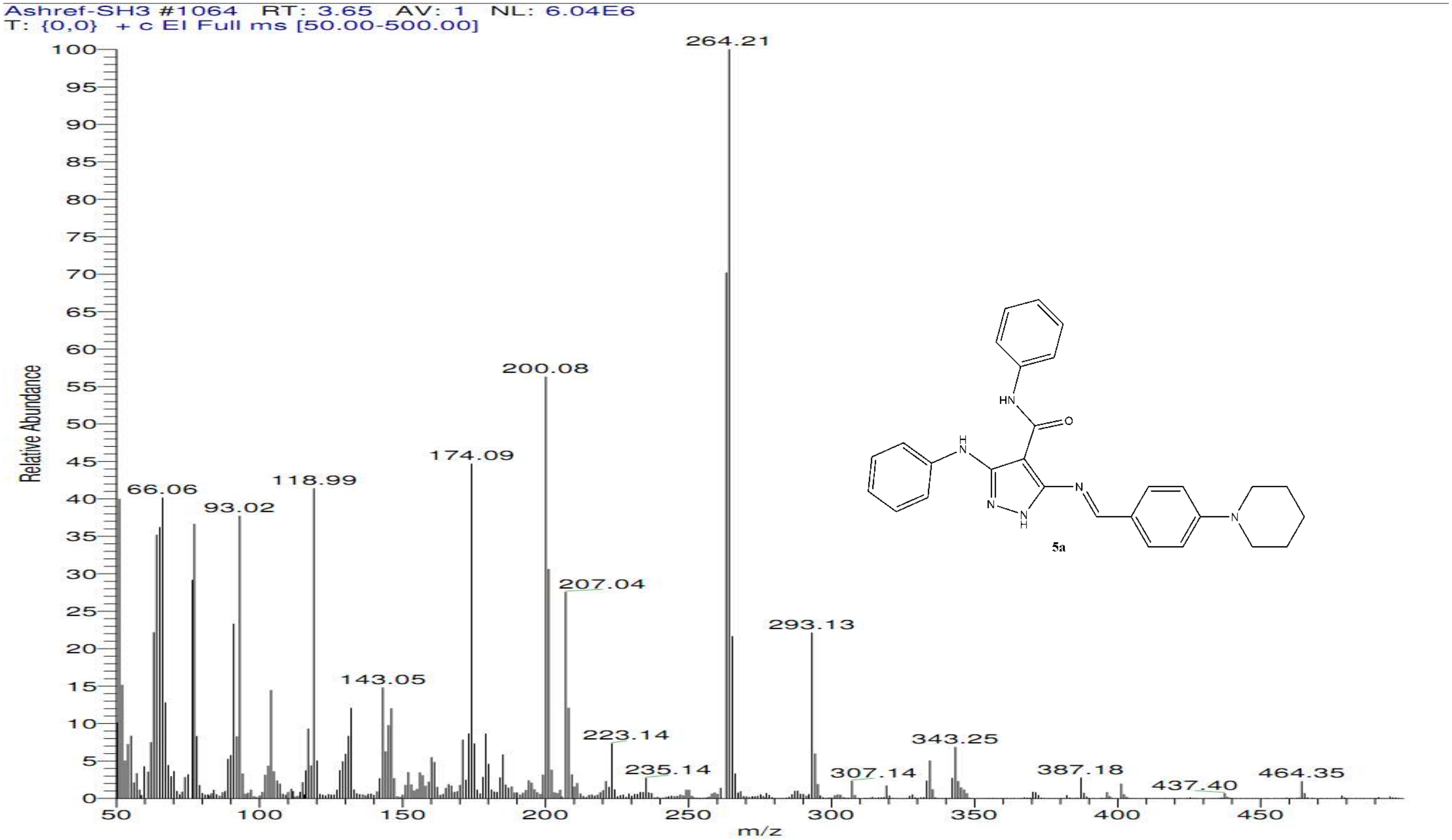

Mass spectrum of Schiff base **5a**

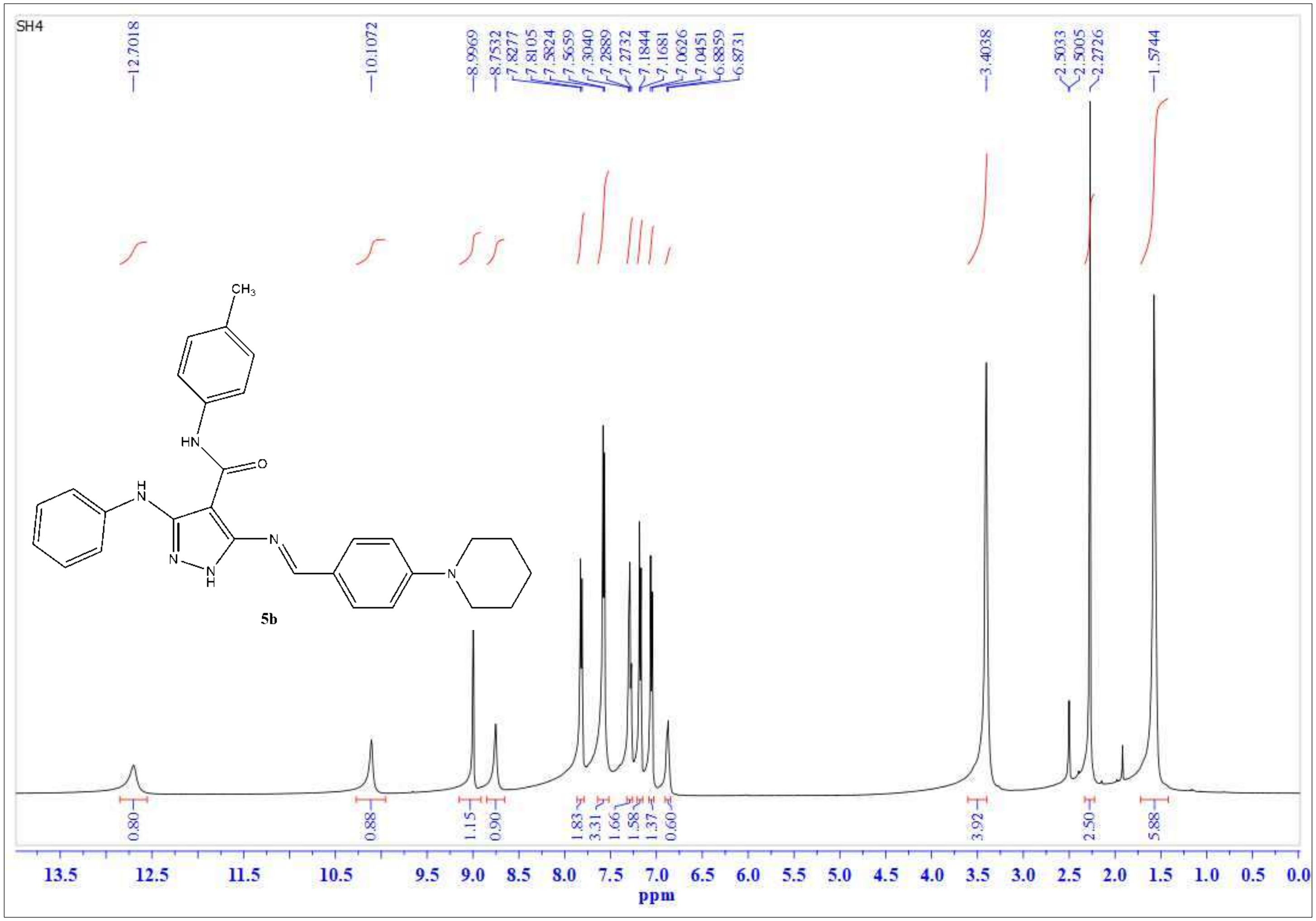

The ^{1}H NMR spectrum of Schiff base **5b**

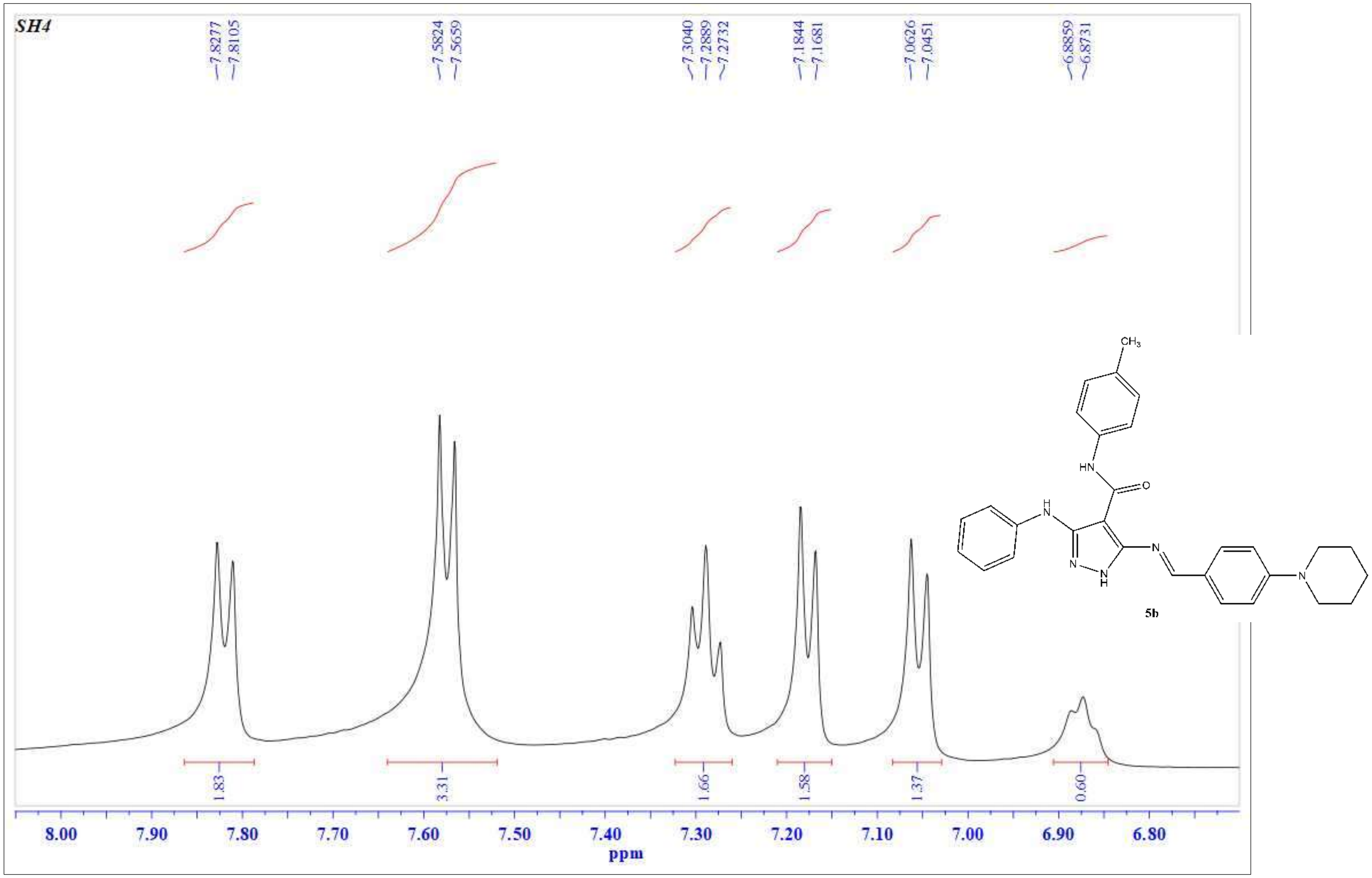

The ^{1}H NMR aromatic region spectrum of Schiff base **5b**

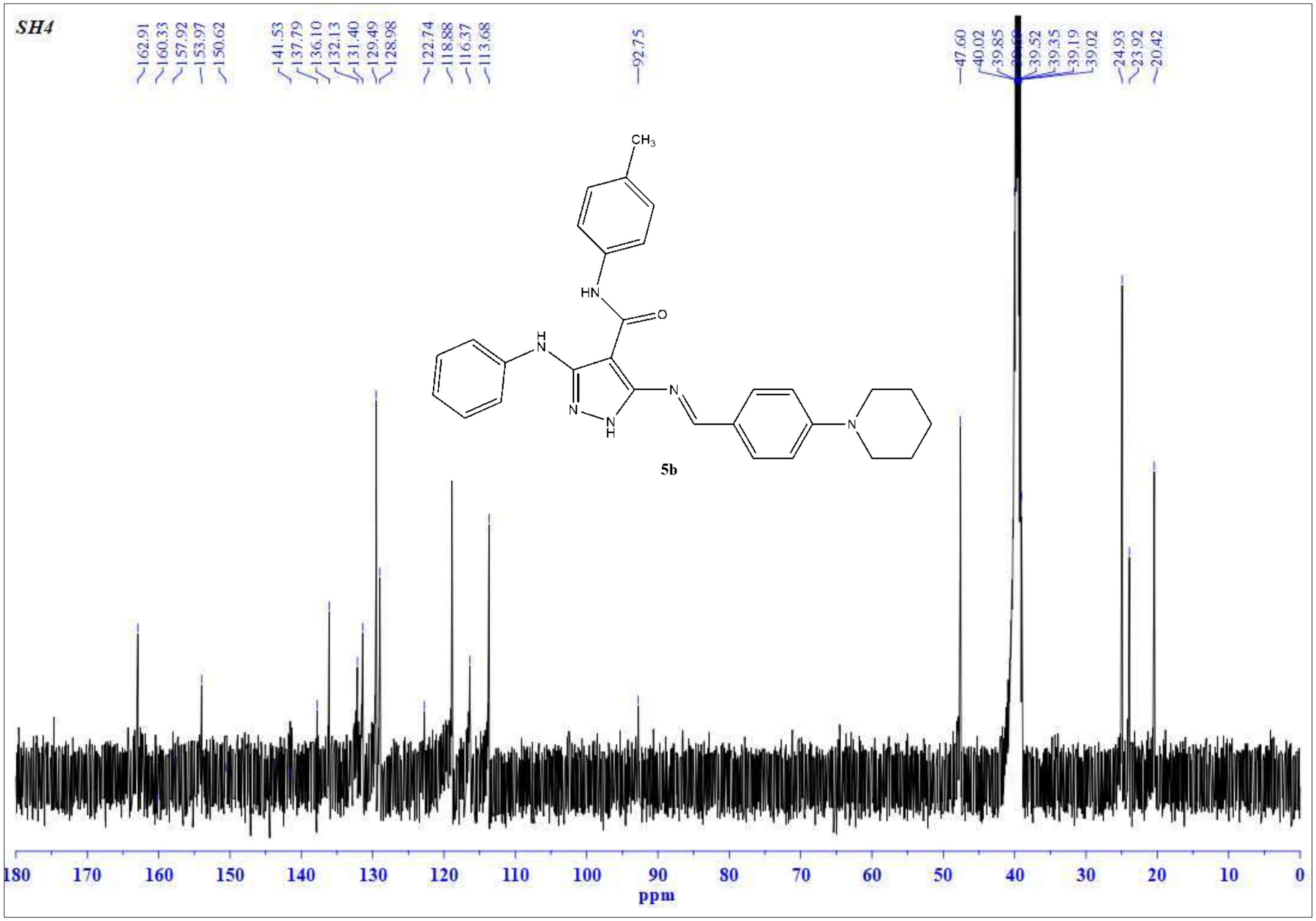

The ^{13}C NMR spectrum of Schiff base **5b**

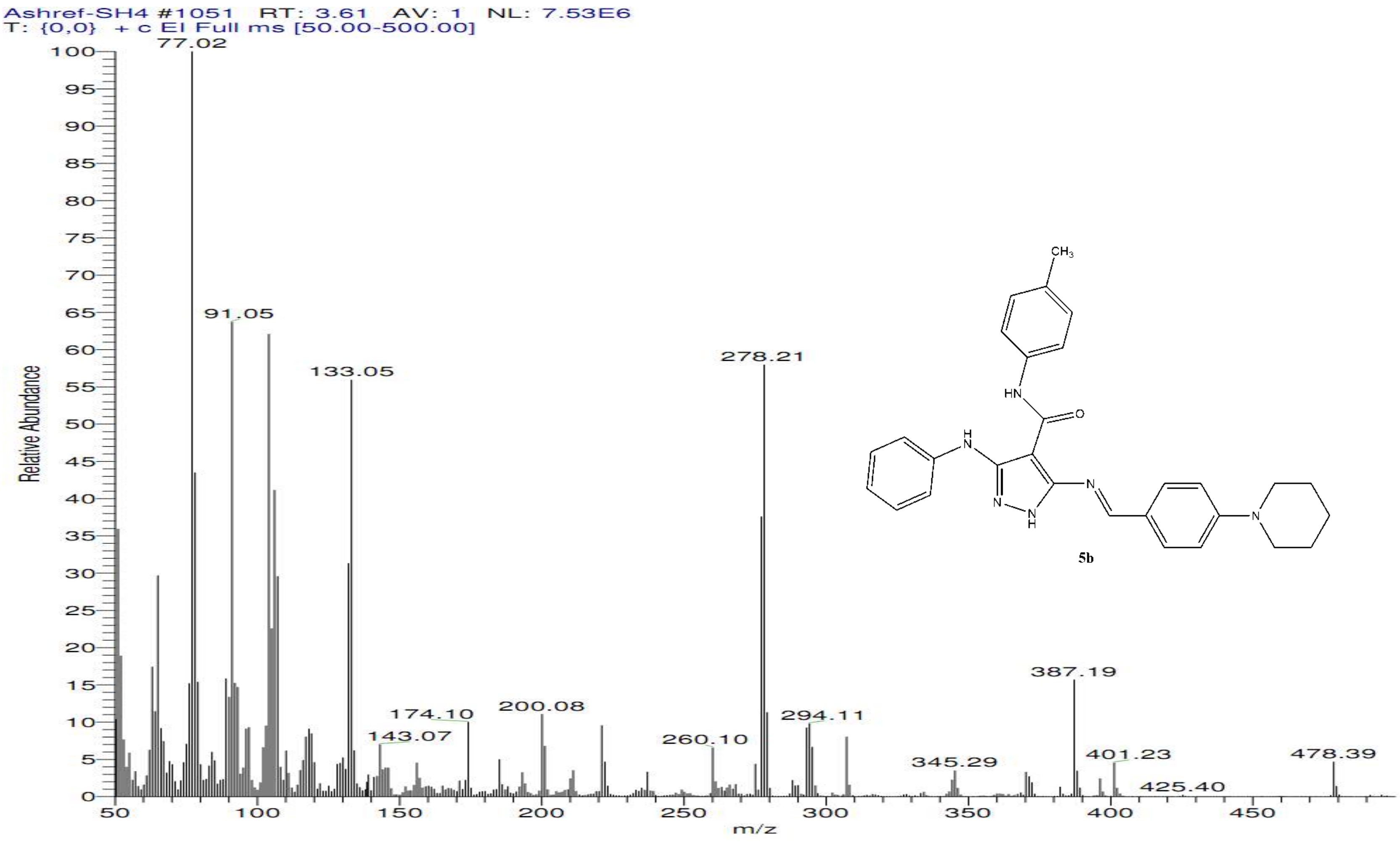

Mass spectrum of Schiff base **5b**

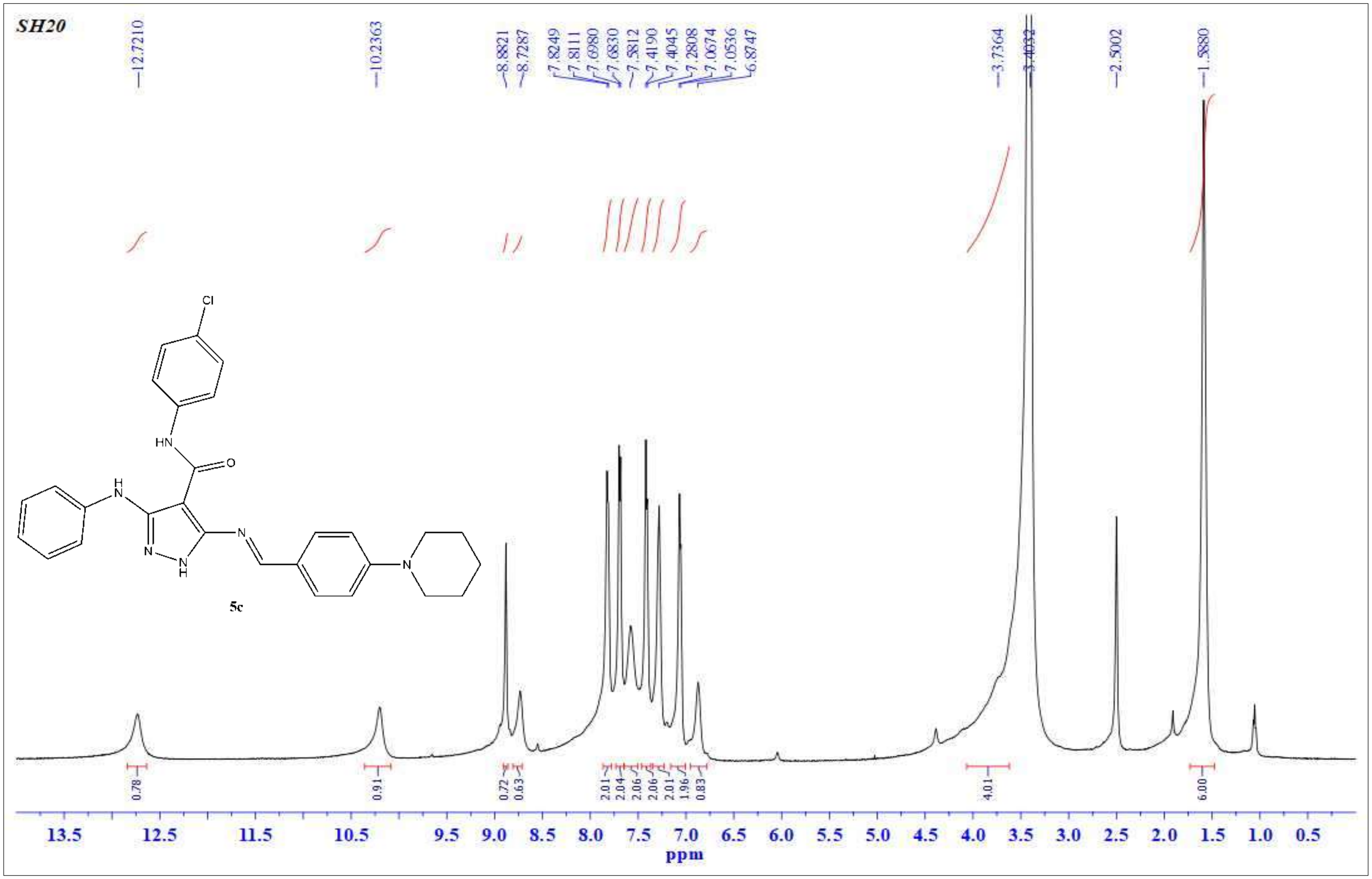

The ^{1}H NMR spectrum of Schiff base **5c**

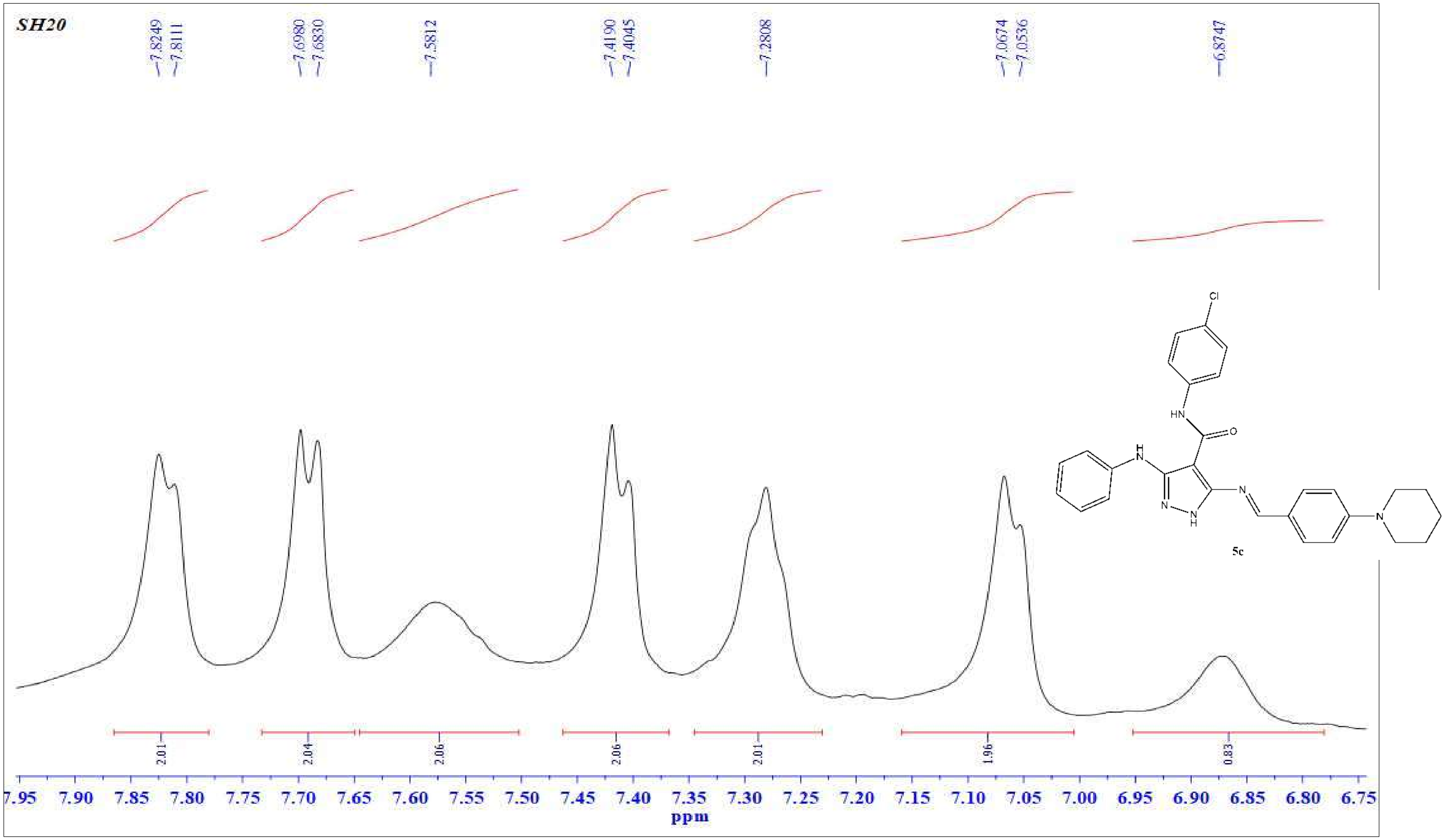

The ^{1}H NMR aromatic region spectrum of Schiff base **5c**

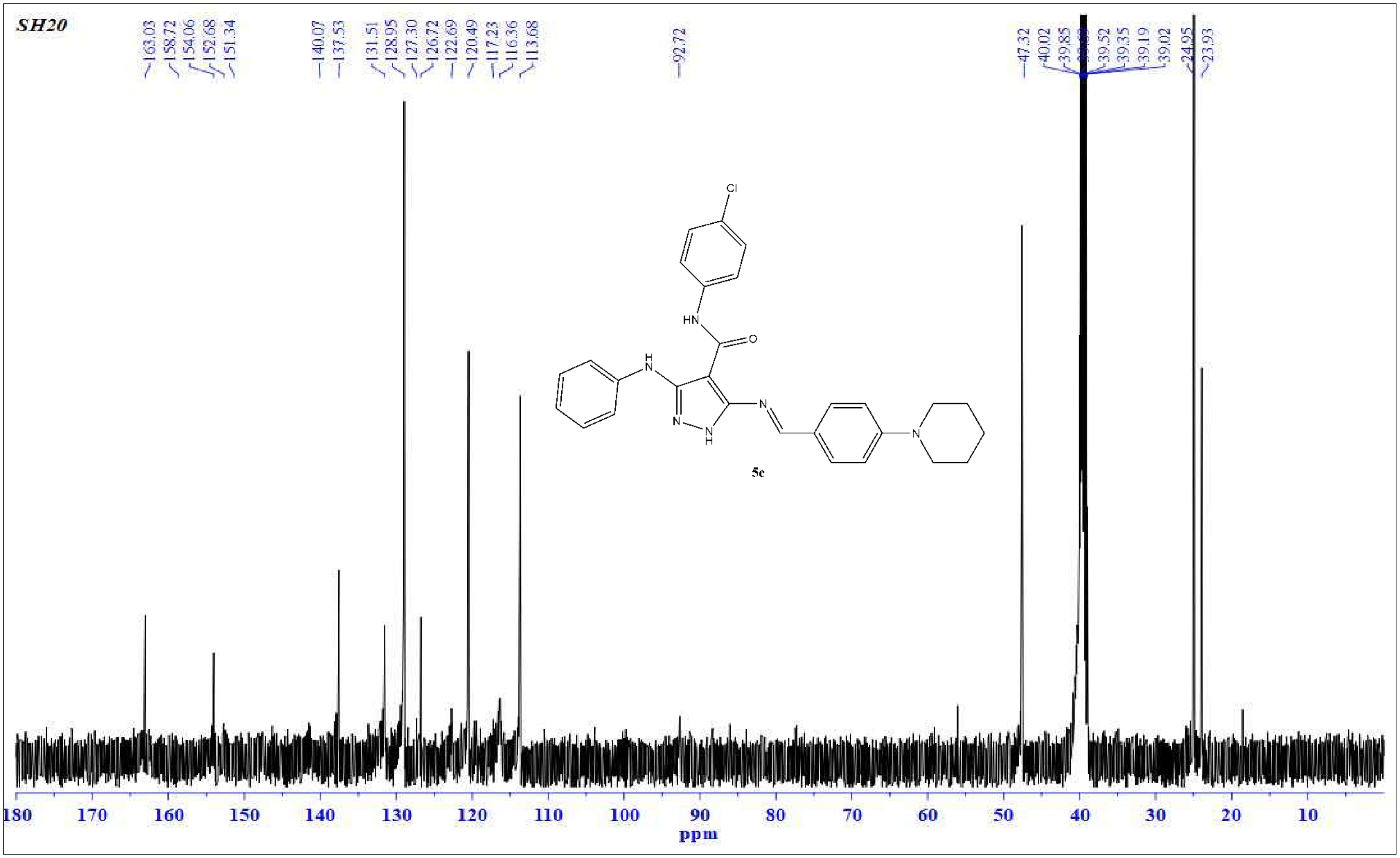

The ^{13}C NMR spectrum of Schiff base **5c**

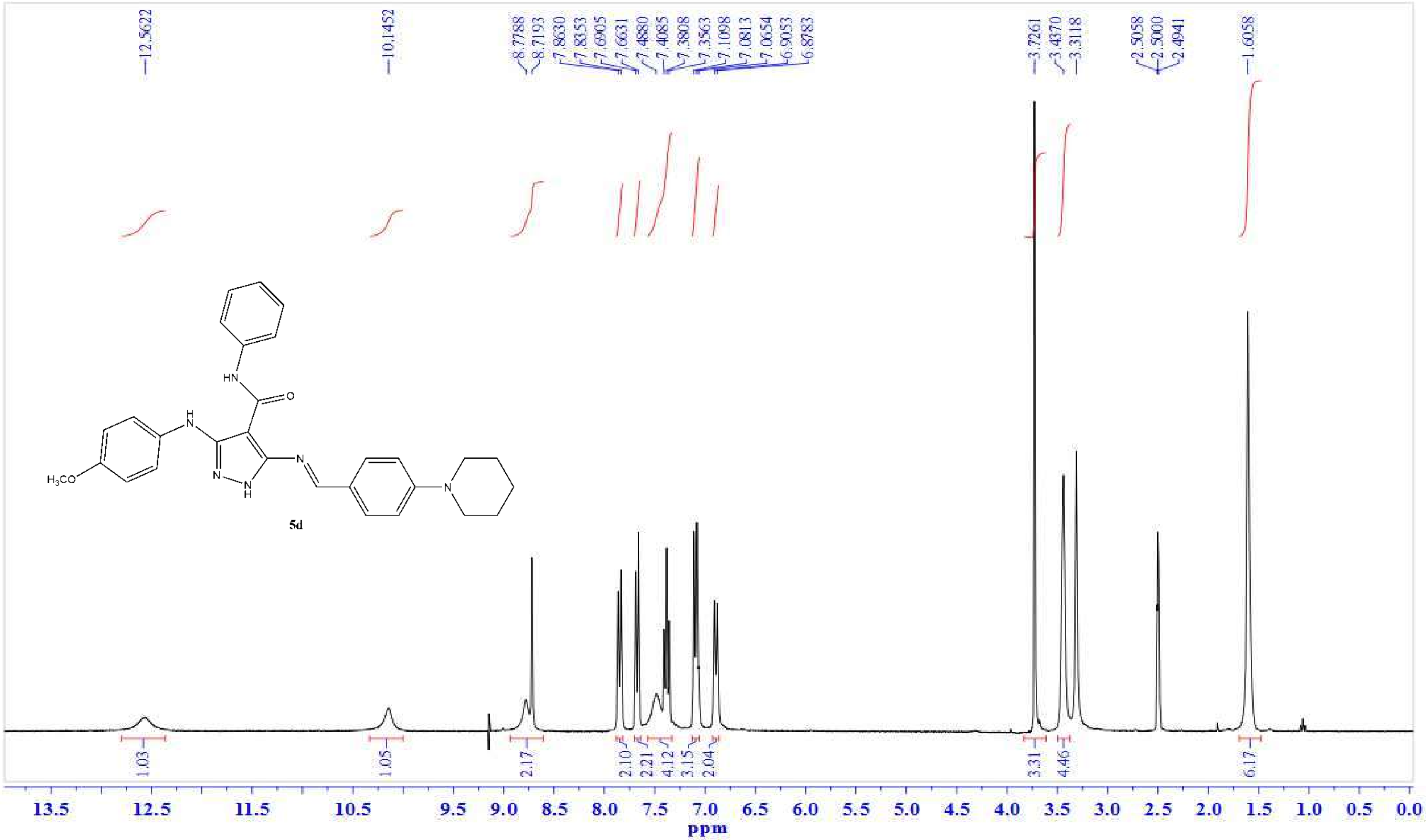

The ^{1}H NMR spectrum of Schiff base **5d**

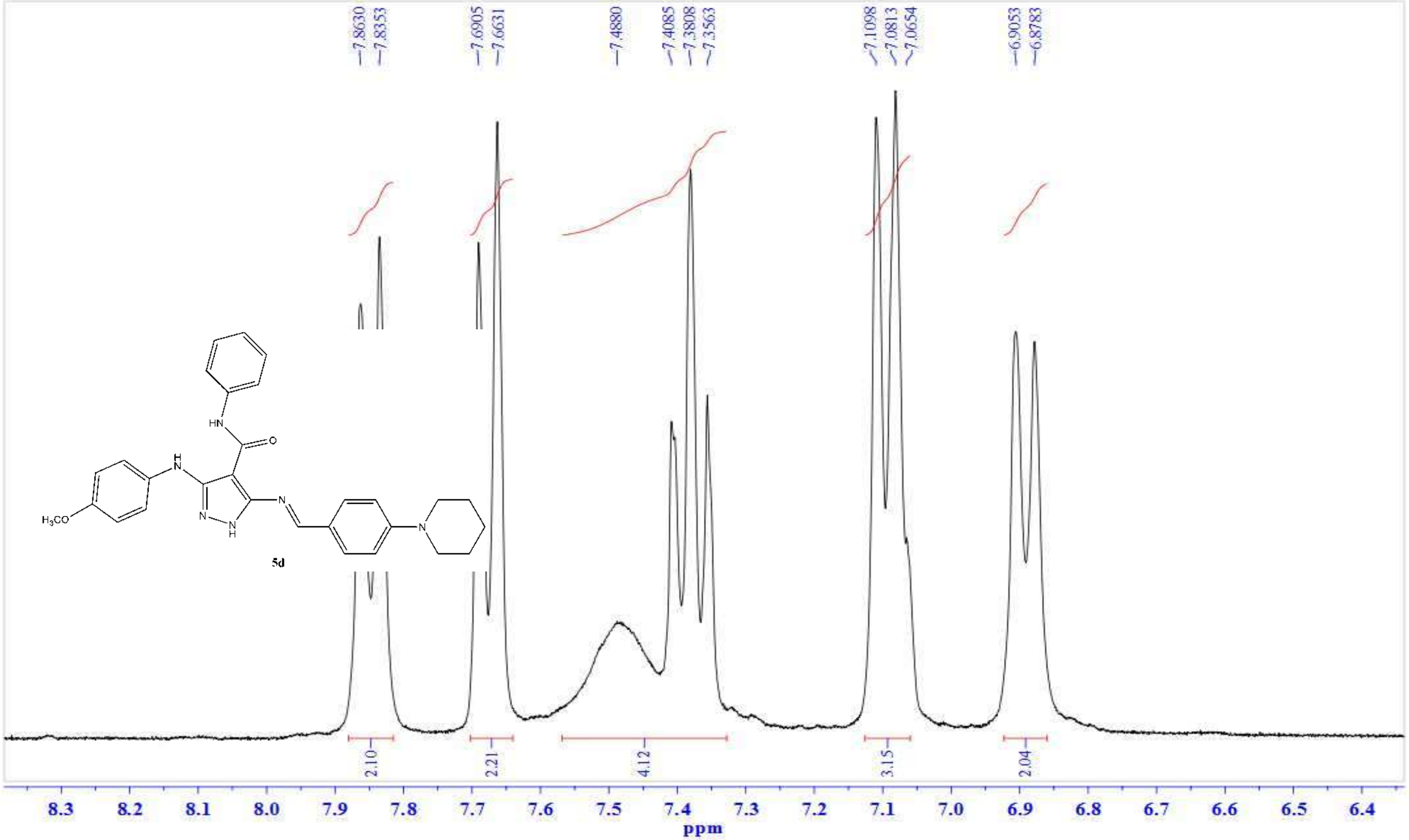

The ^{1}H NMR aromatic region spectrum of Schiff base **5d**

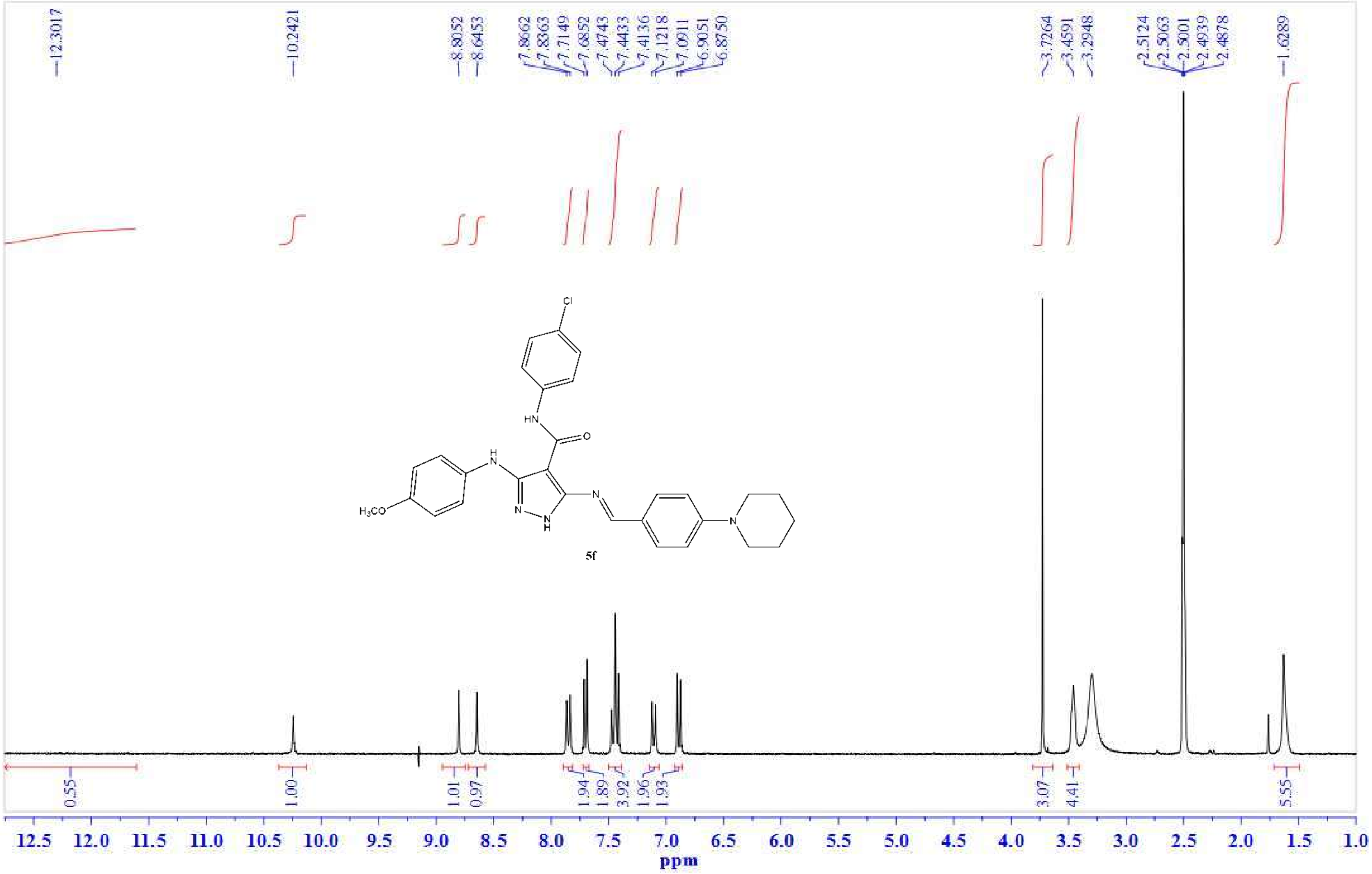

The ^{1}H NMR spectrum of Schiff base **5f**

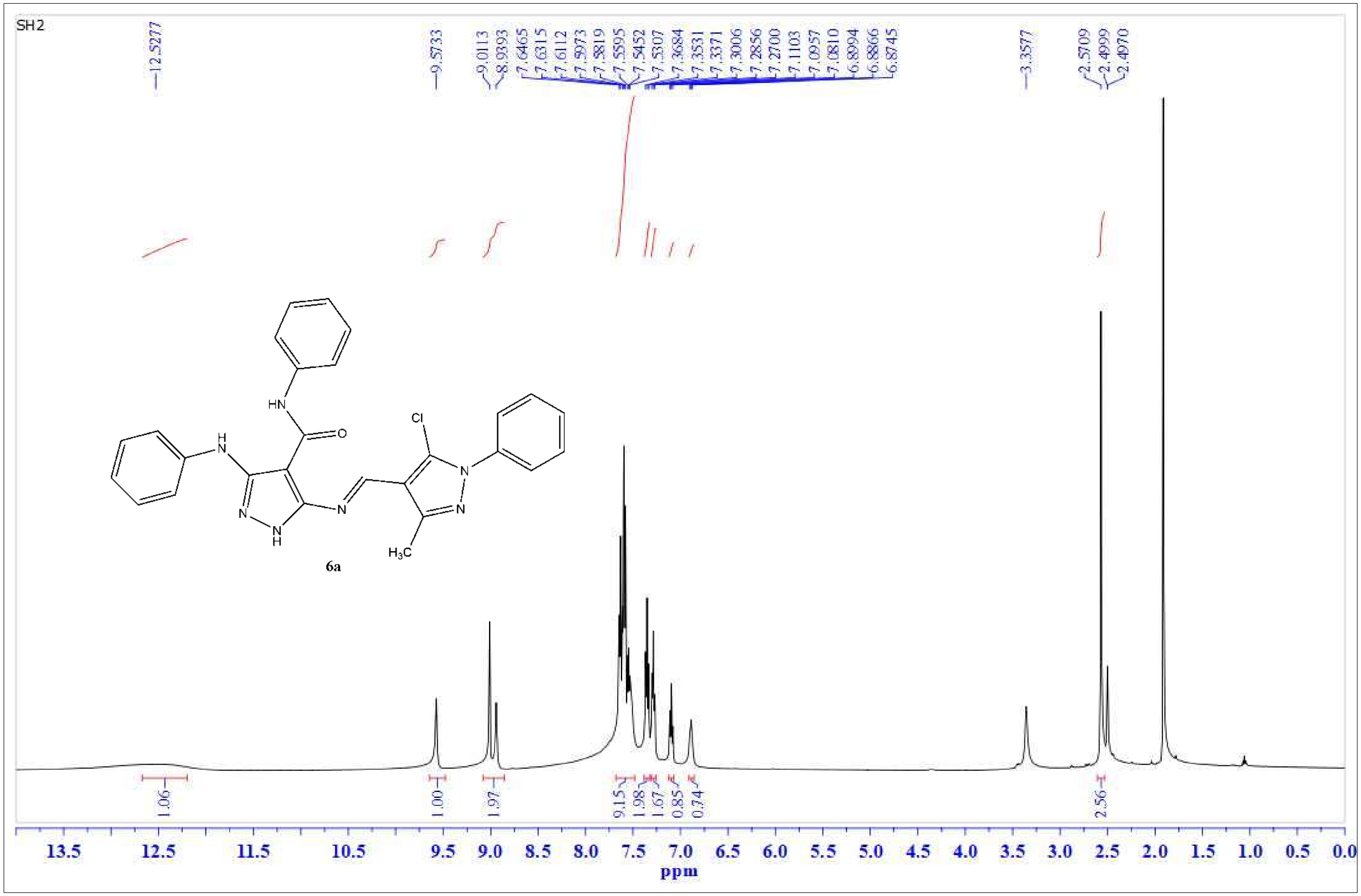

The ^{1}H NMR spectrum of Schiff base **6a**

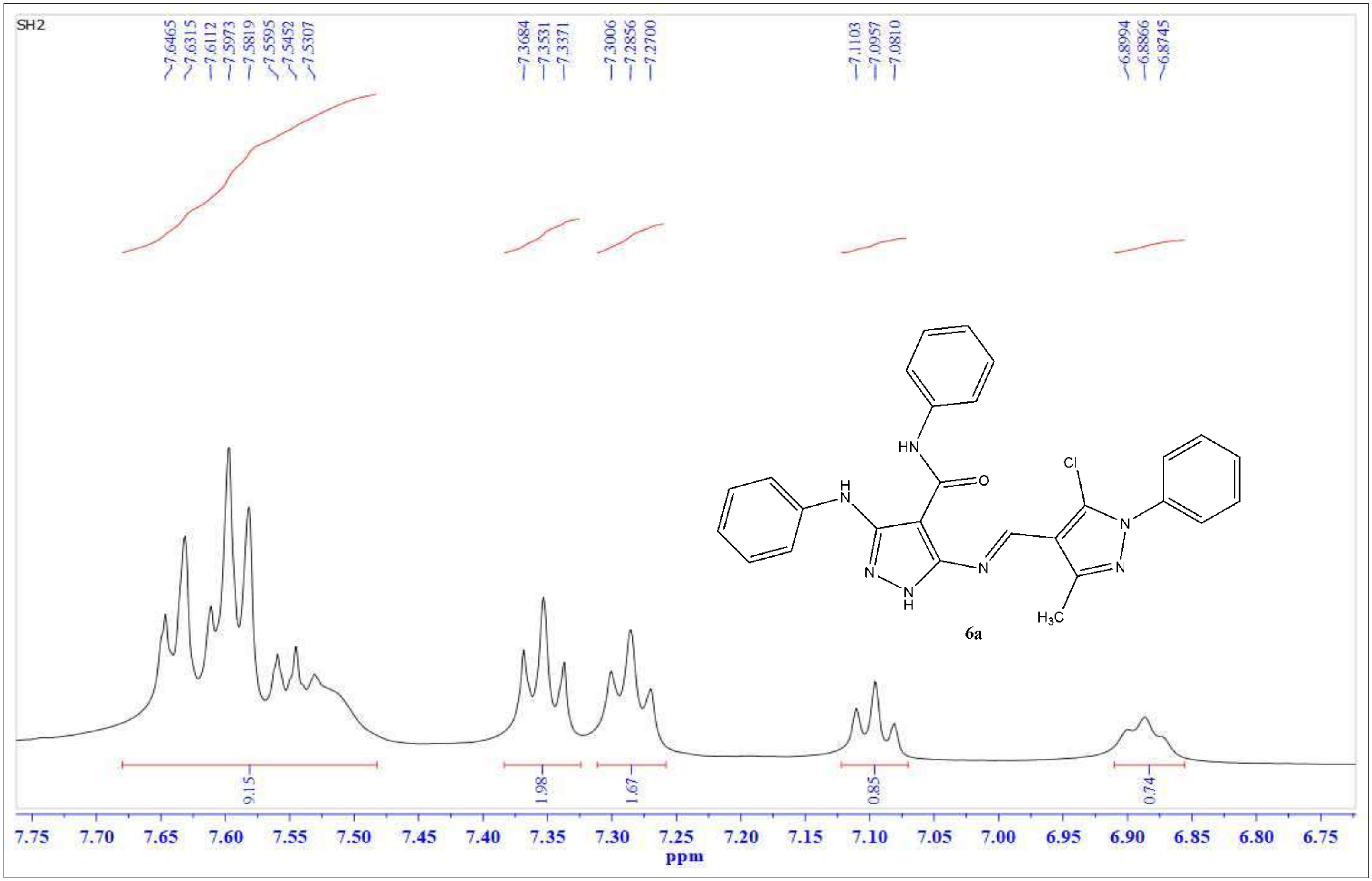

The ^{1}H NMR aromatic region spectrum of Schiff base **6a**

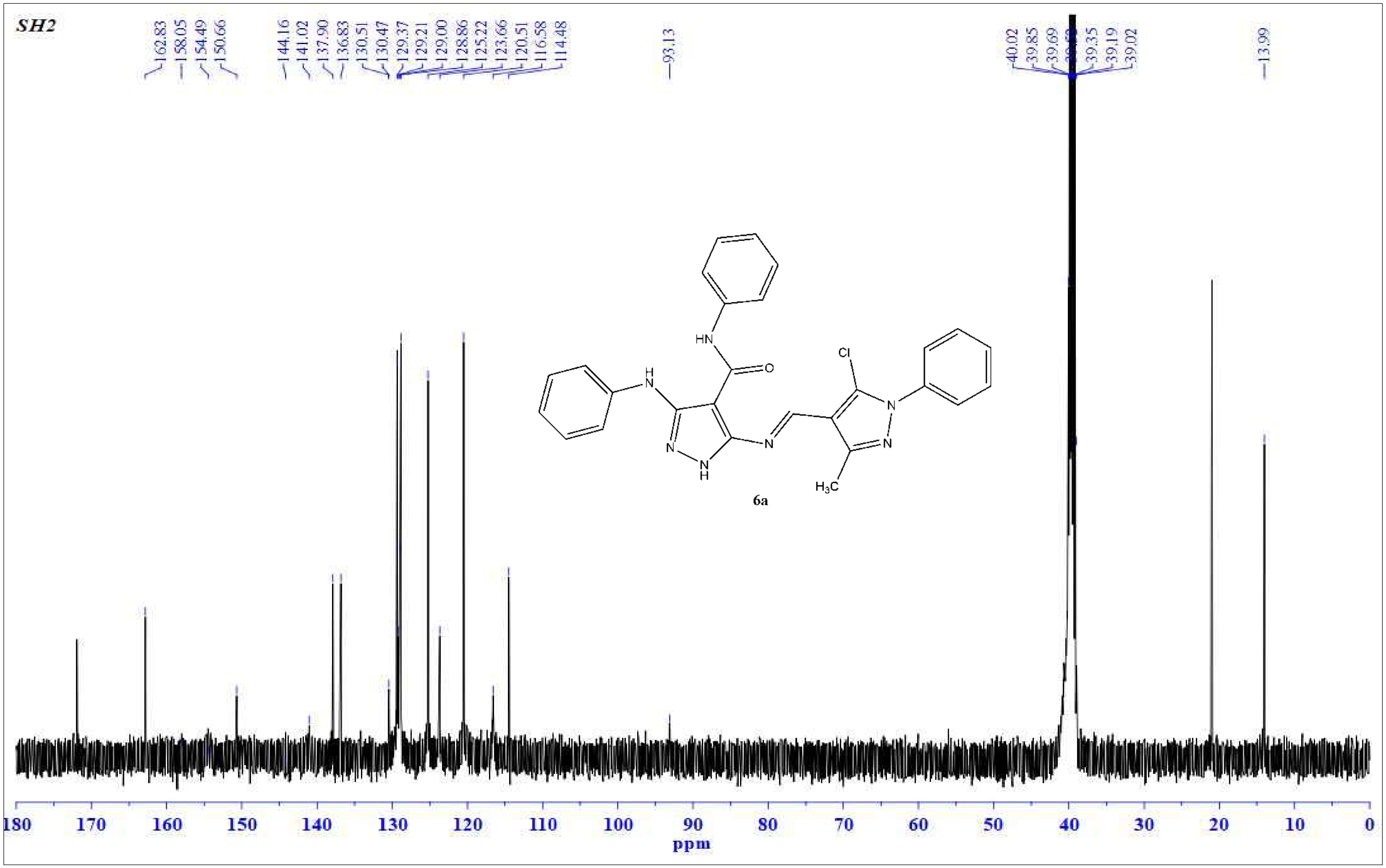

The ^{13}C NMR spectrum of Schiff base **6a**

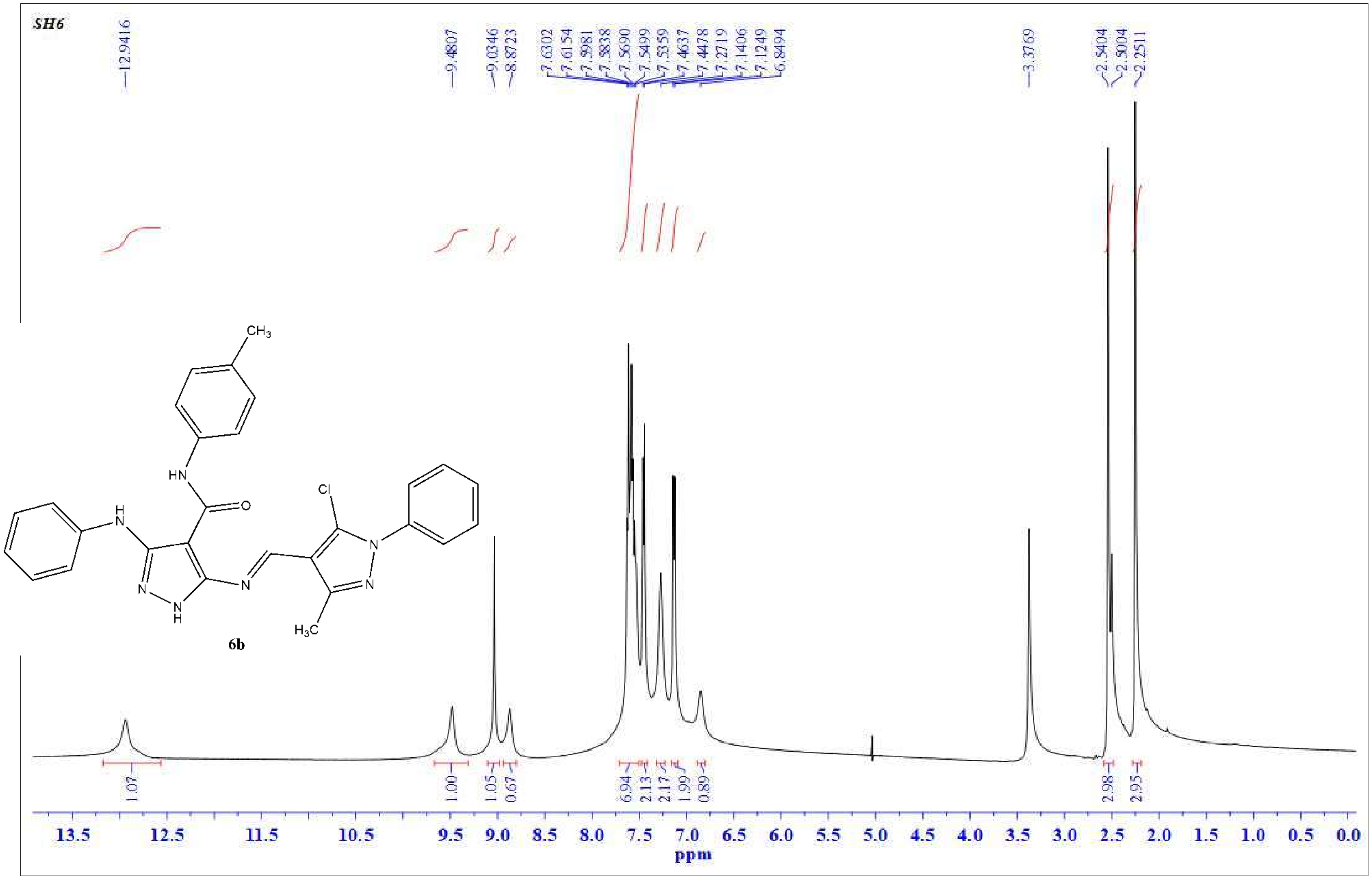

The ^{1}H NMR spectrum of Schiff base **6b**

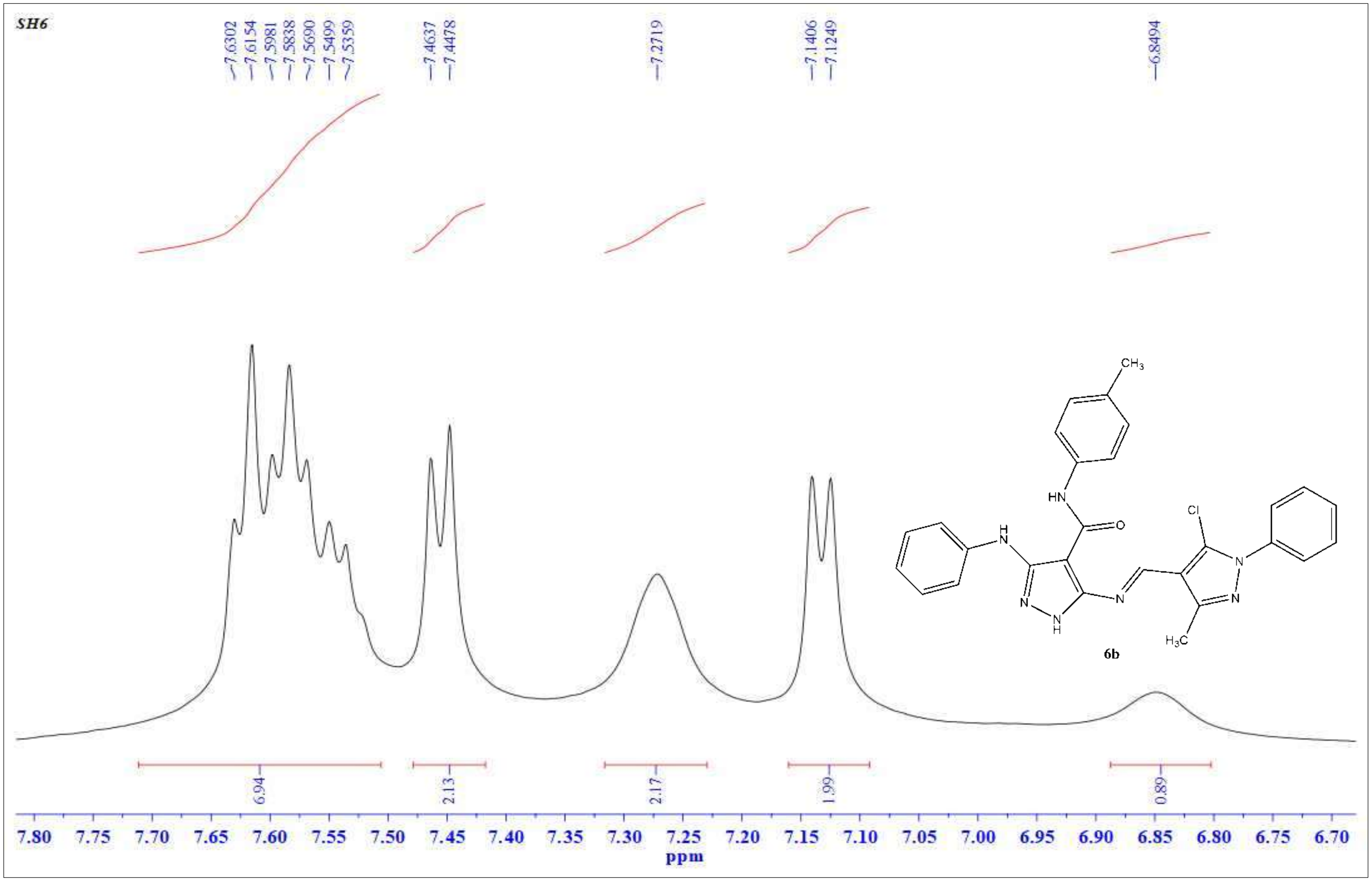

The ^{1}H NMR aromatic region spectrum of Schiff base **6b**

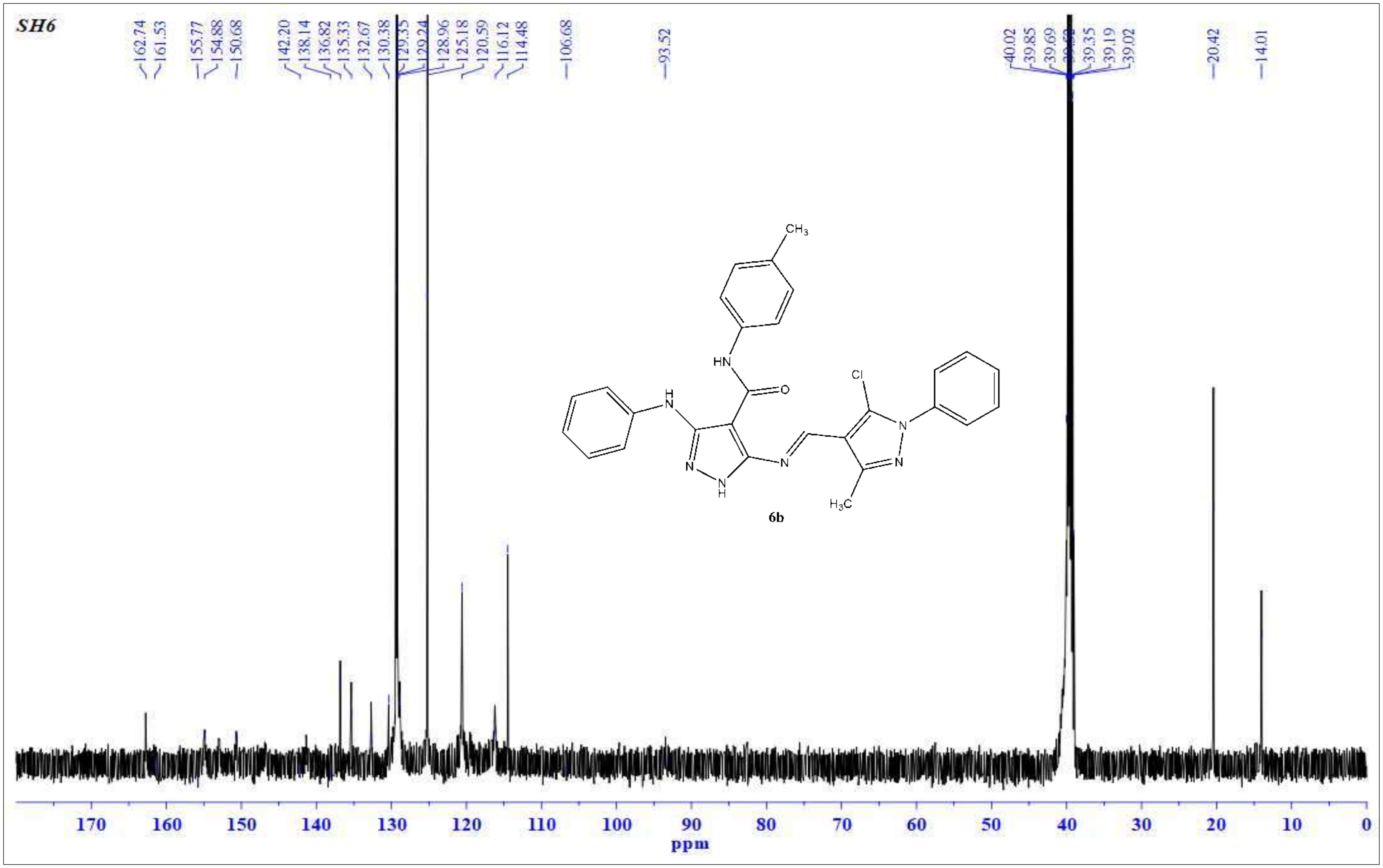

The ^{13}C NMR spectrum of Schiff base **6b**

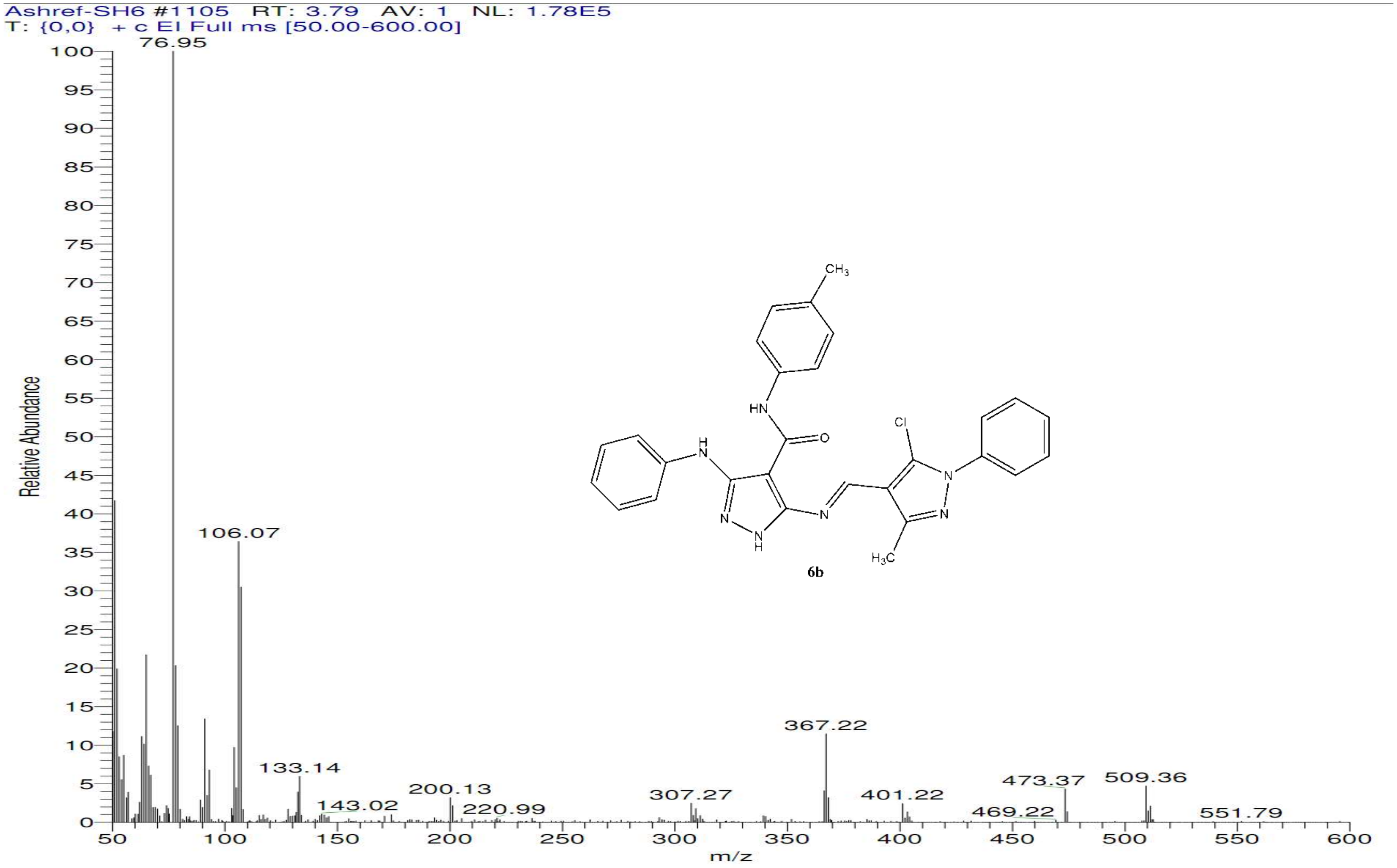

Mass spectrum of Schiff base **6b**

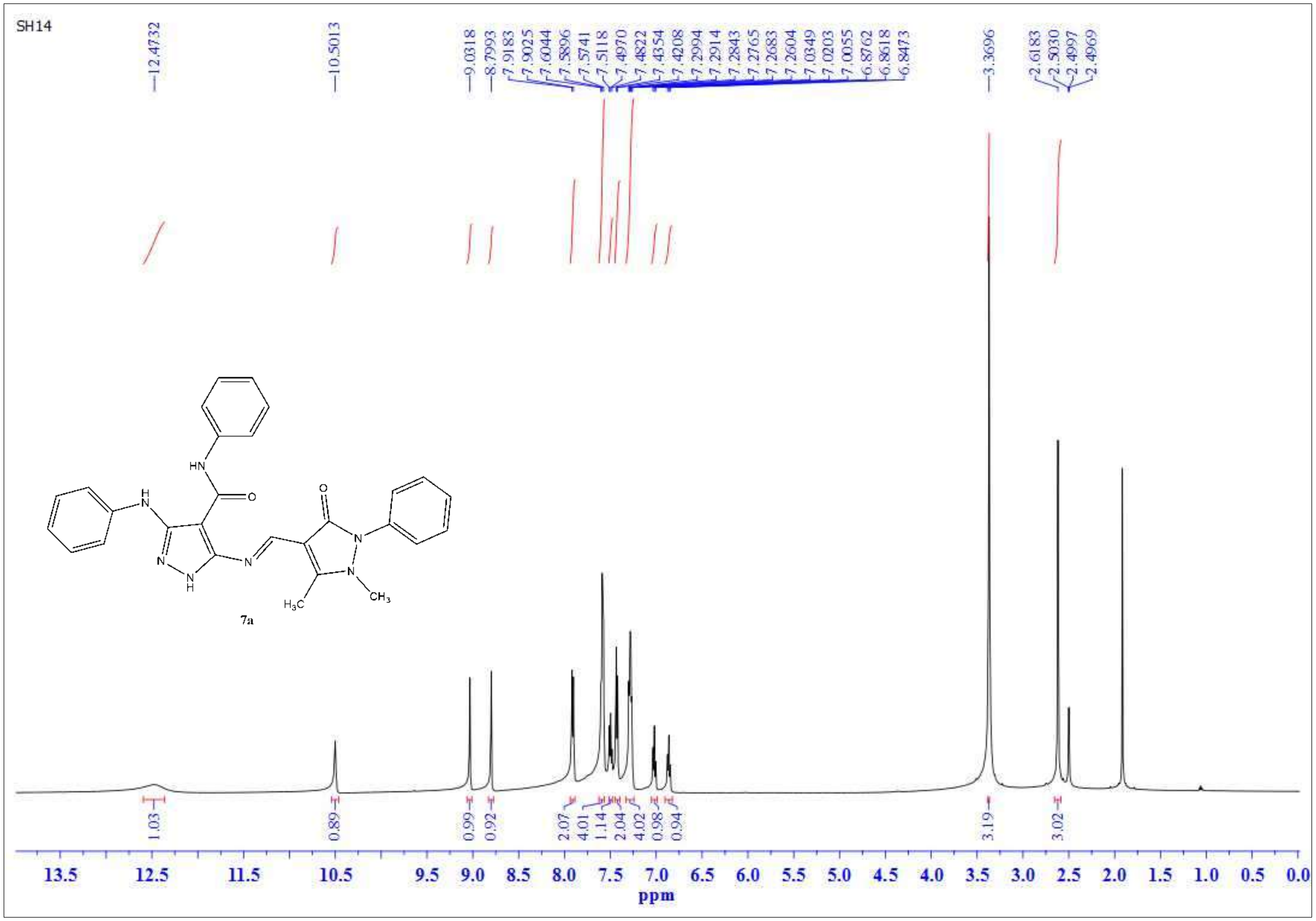

The ^{1}H NMR spectrum of Schiff base **7a**

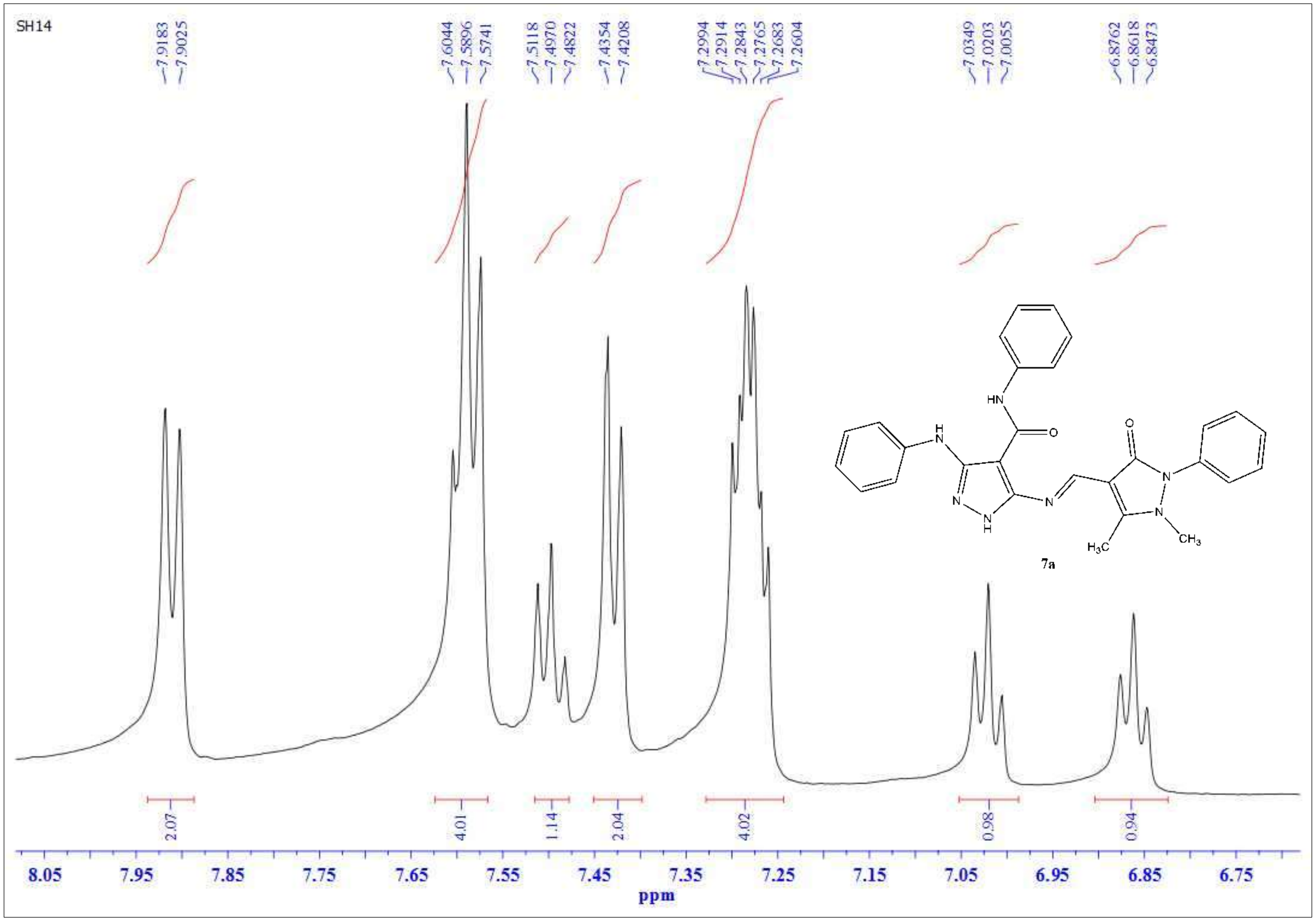

The ^{1}H NMR aromatic region spectrum of Schiff base **7a**

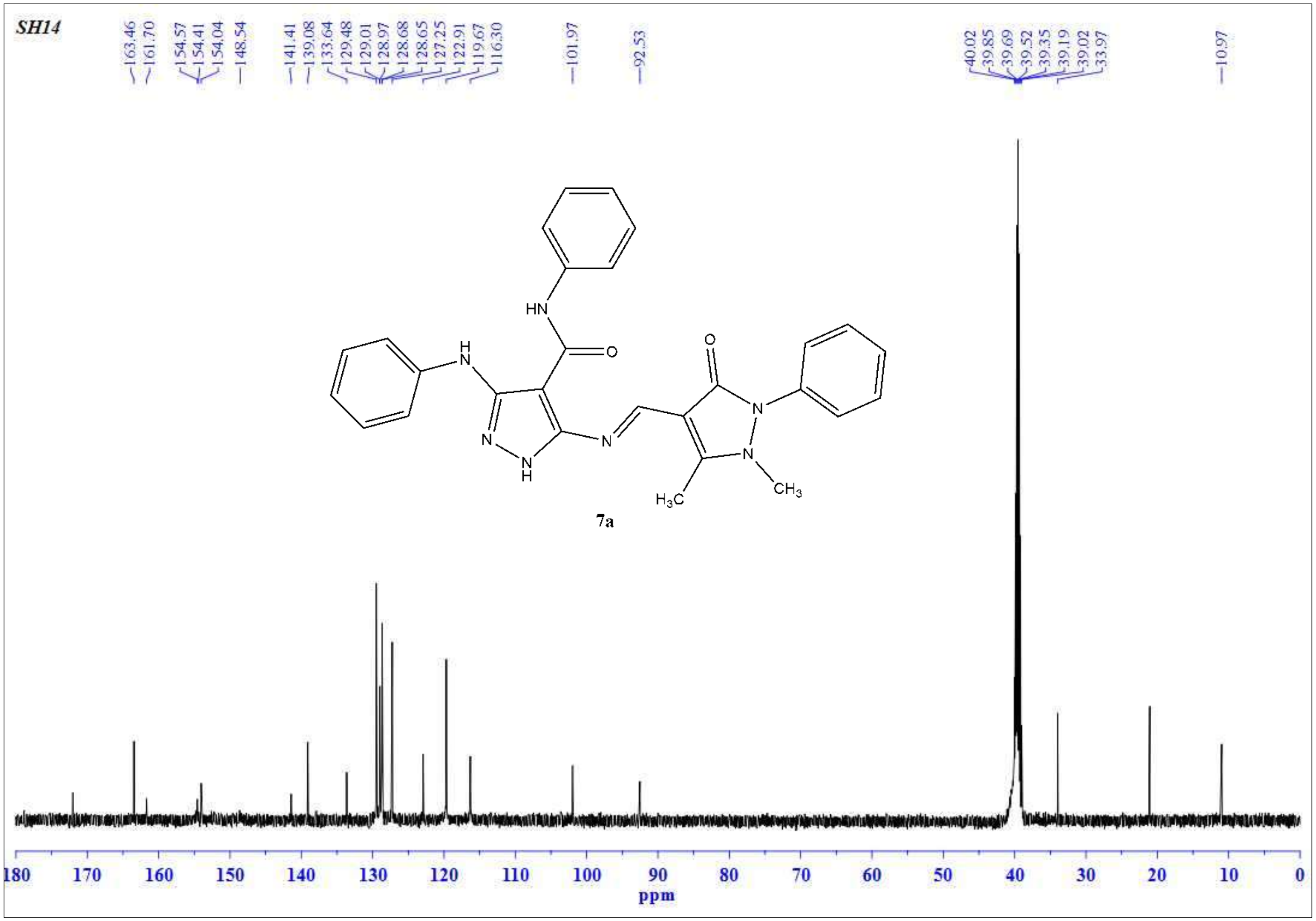

The ^{13}C NMR spectrum of Schiff base **7a**

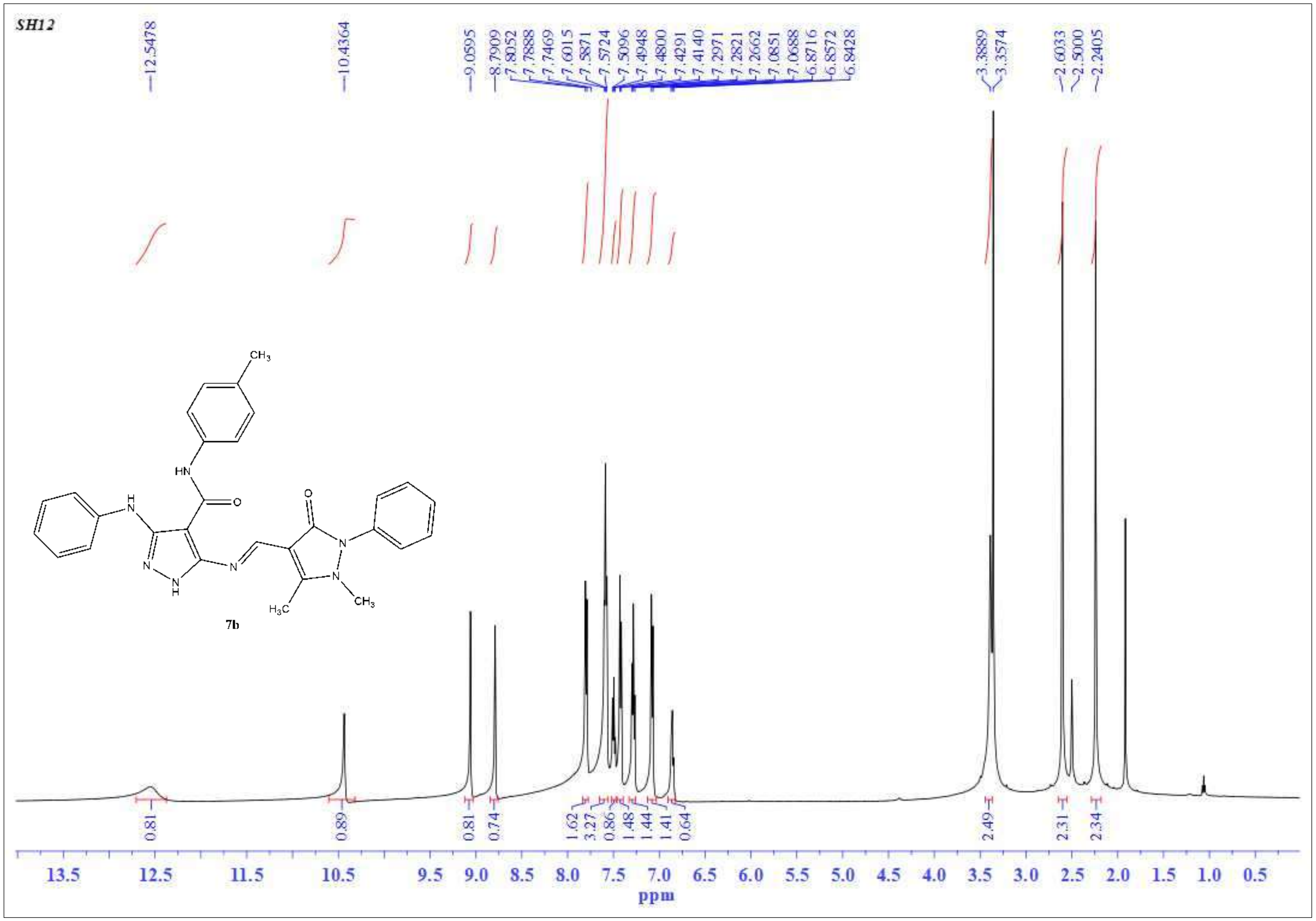

The ^{1}H NMR spectrum of Schiff base **7b**

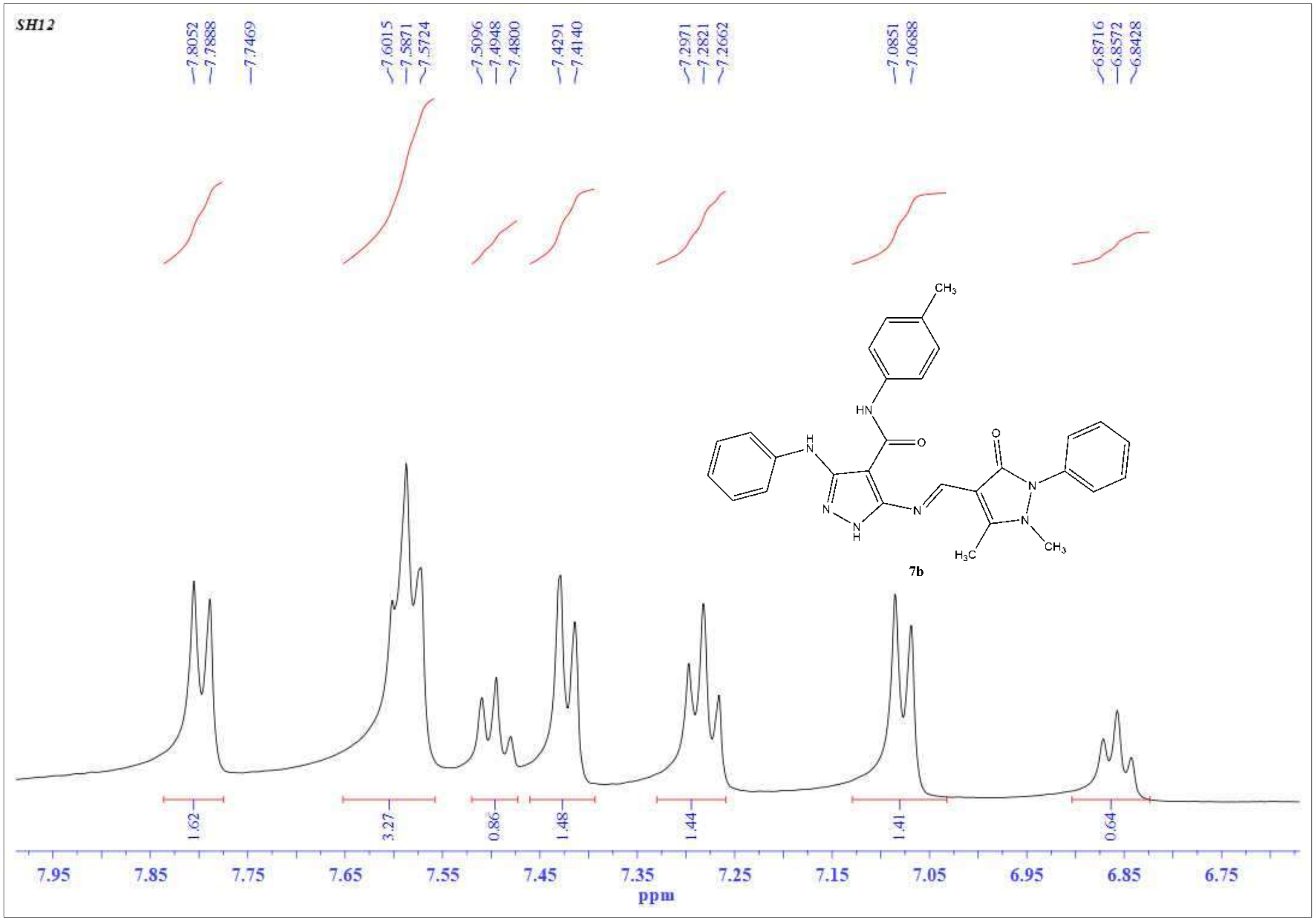

The ^{1}H NMR aromatic region spectrum of Schiff base **7b**

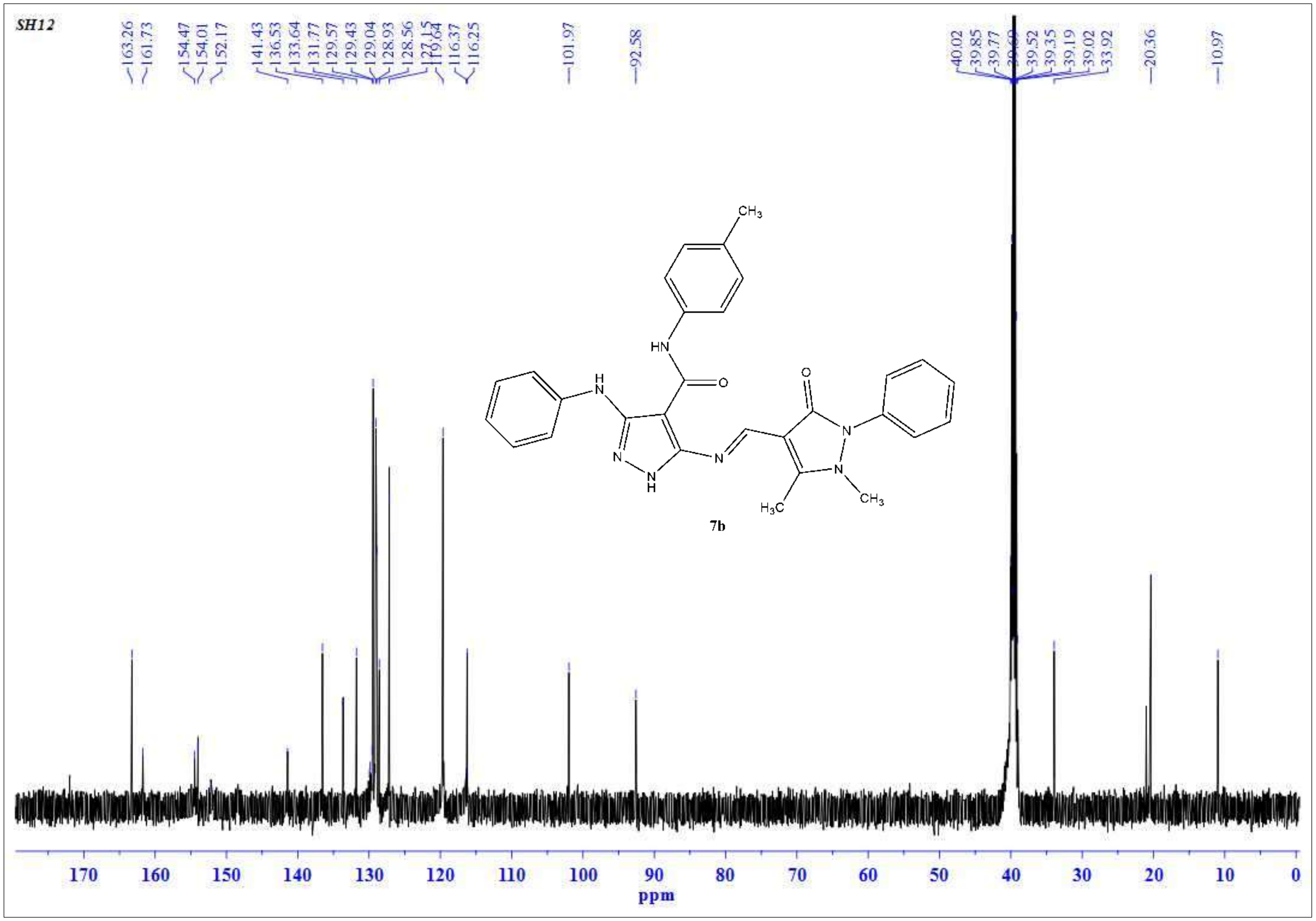

The ^{13}C NMR spectrum of Schiff base **7b**

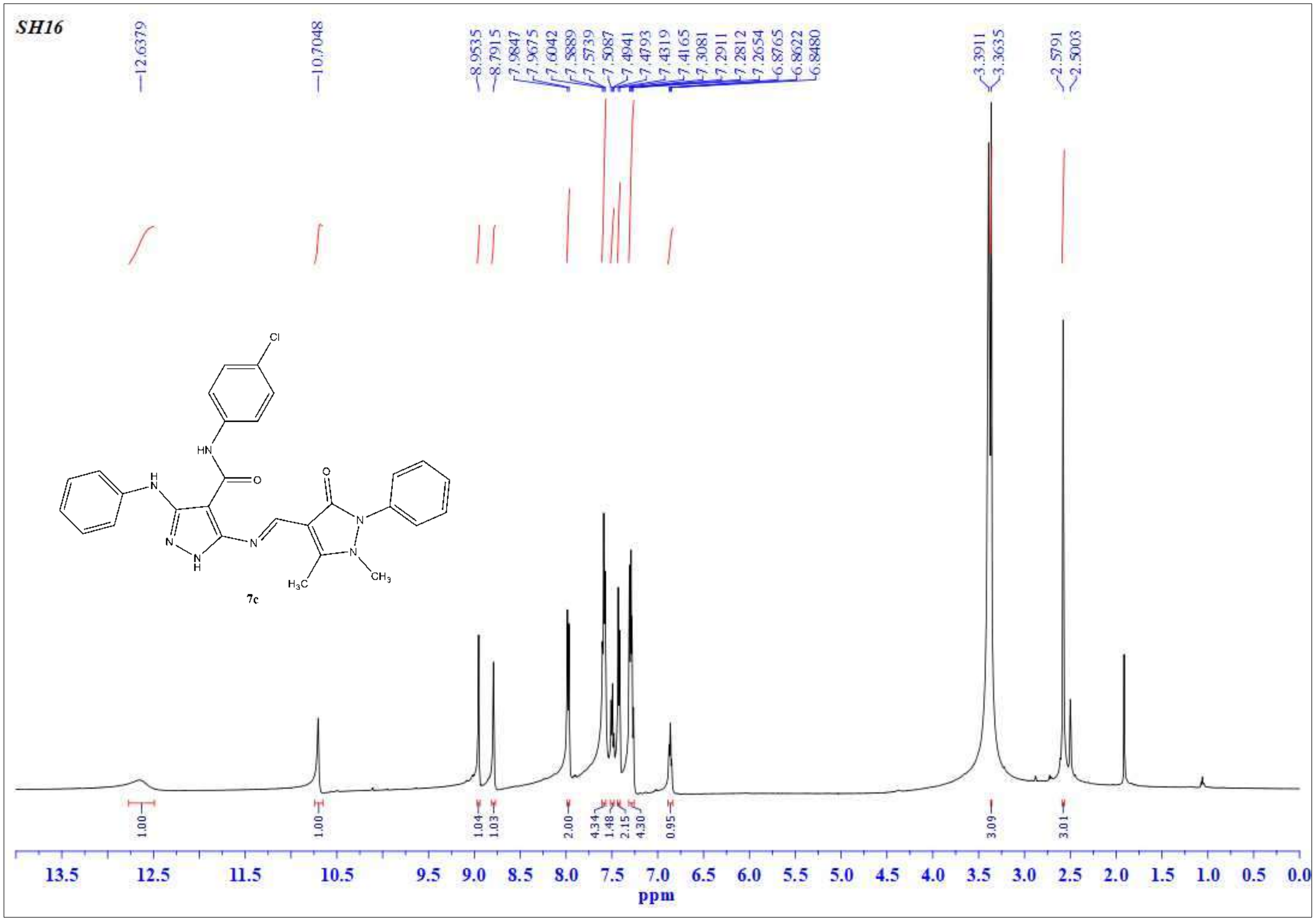

The ^{1}H NMR spectrum of Schiff base **7c**

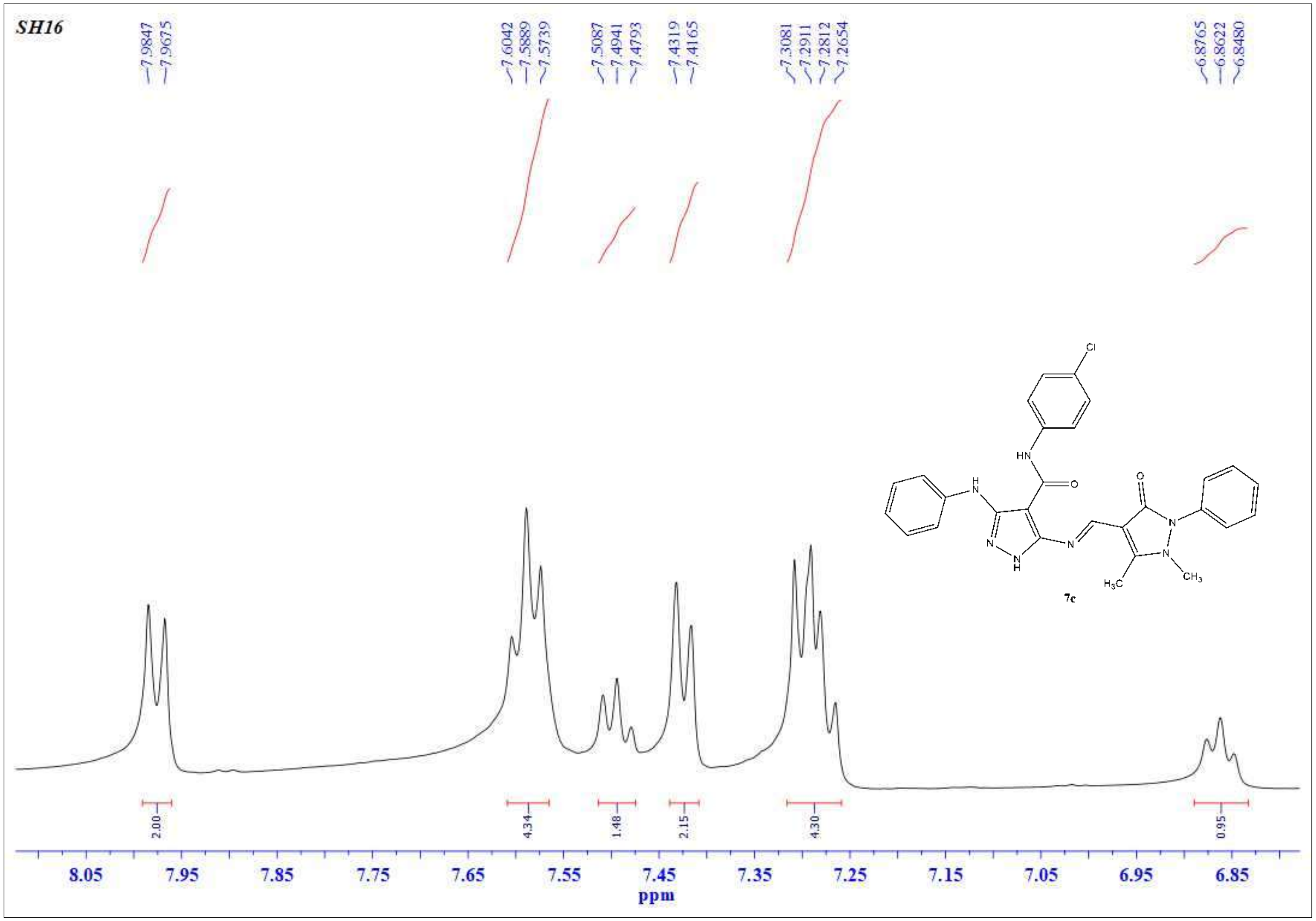

The ^{1}H NMR aromatic region spectrum of Schiff base **7c**

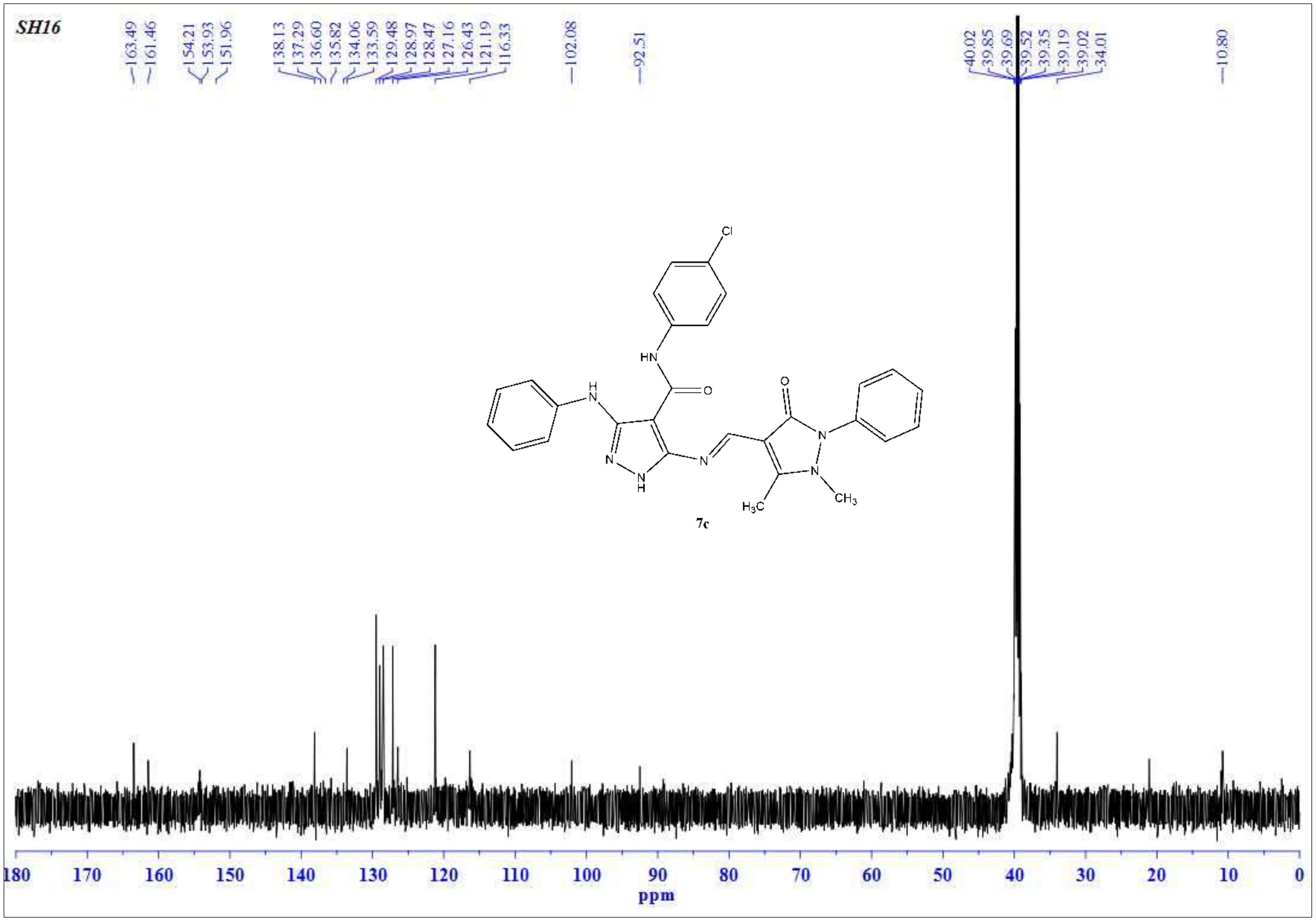

The ^{13}C NMR spectrum of Schiff base **7c**

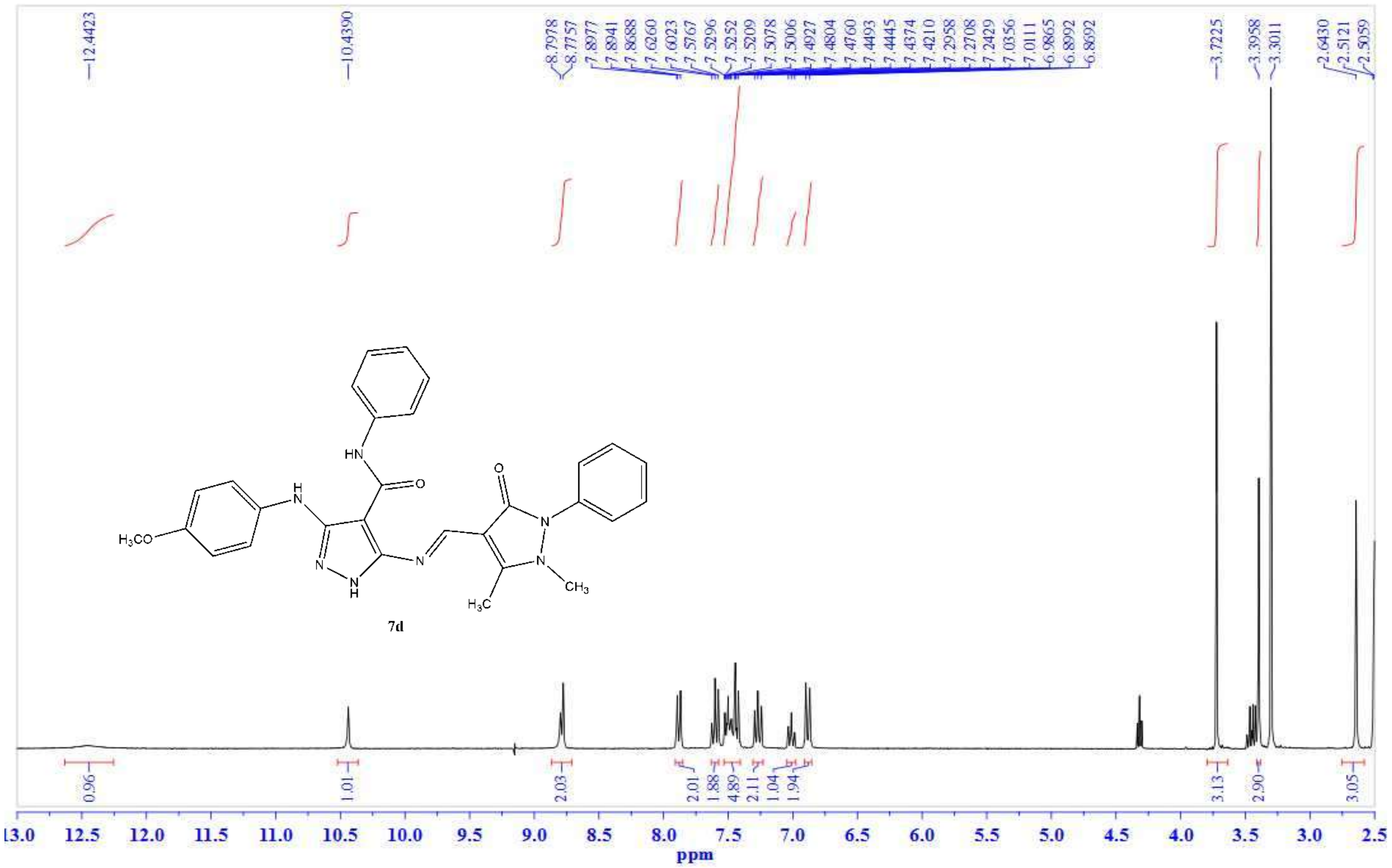

The ^{1}H NMR spectrum of Schiff base **7d**

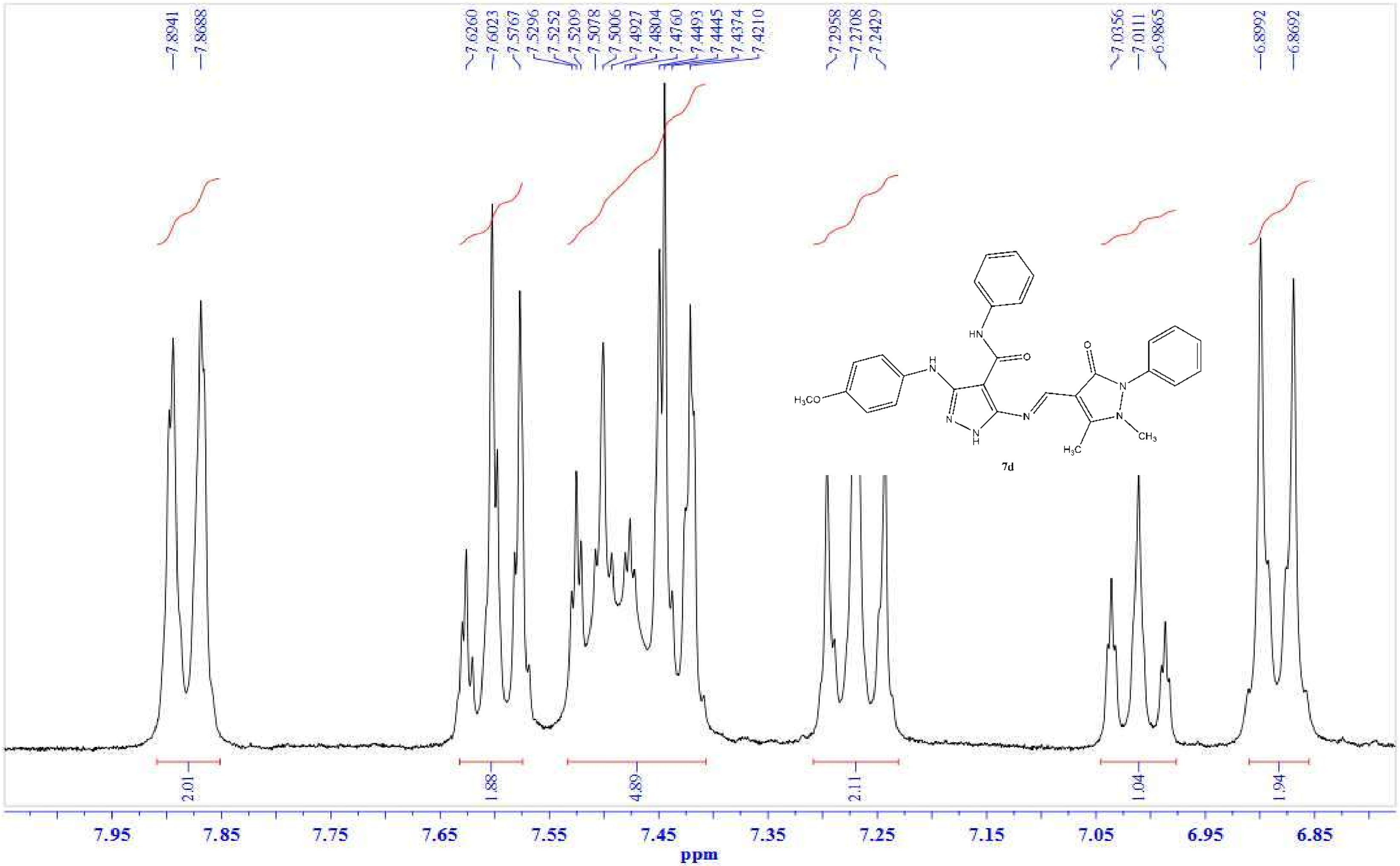

The ^{1}H NMR aromatic region spectrum of Schiff base **7d**

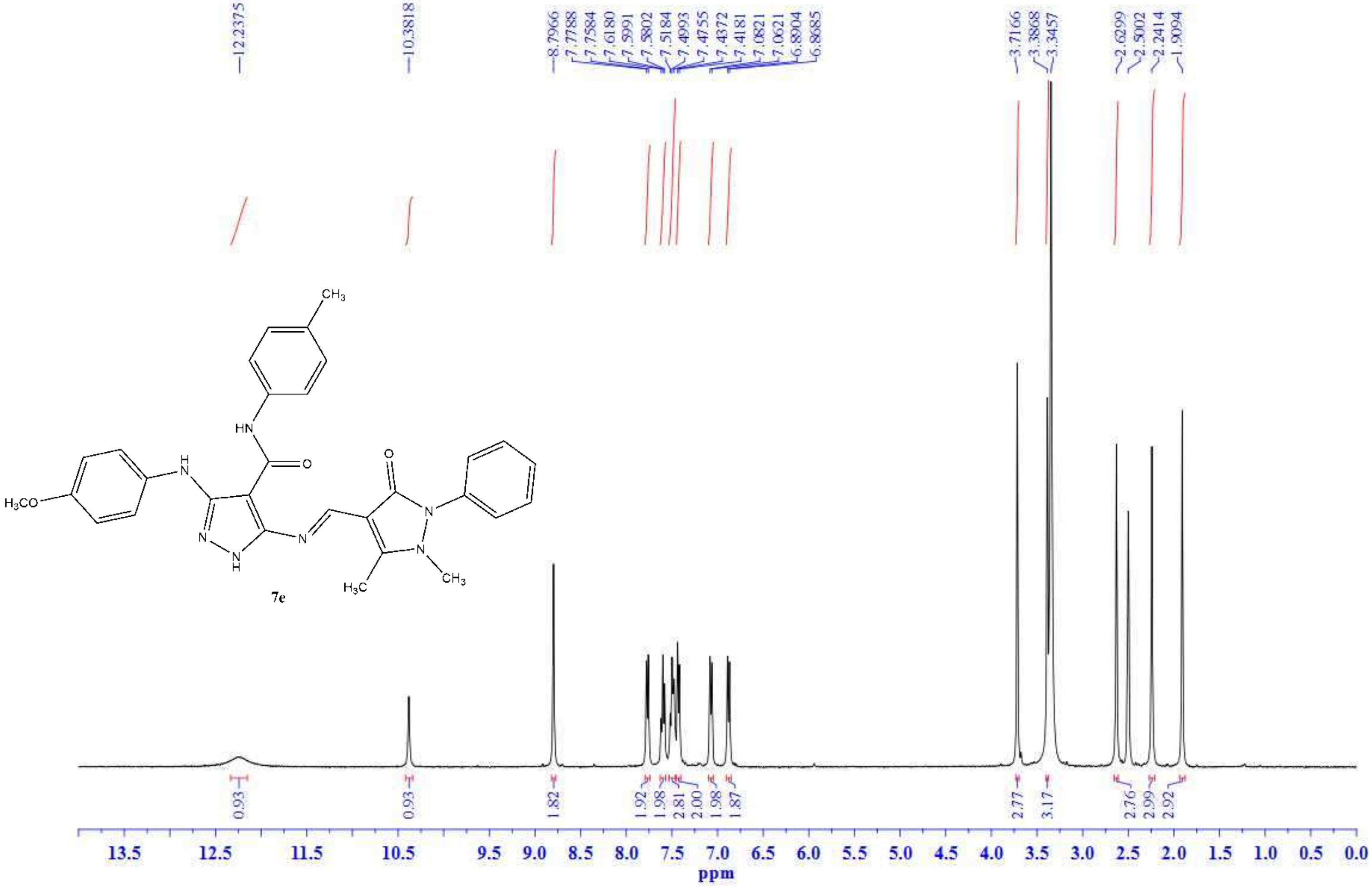

The ^{1}H NMR spectrum of Schiff base **7e**

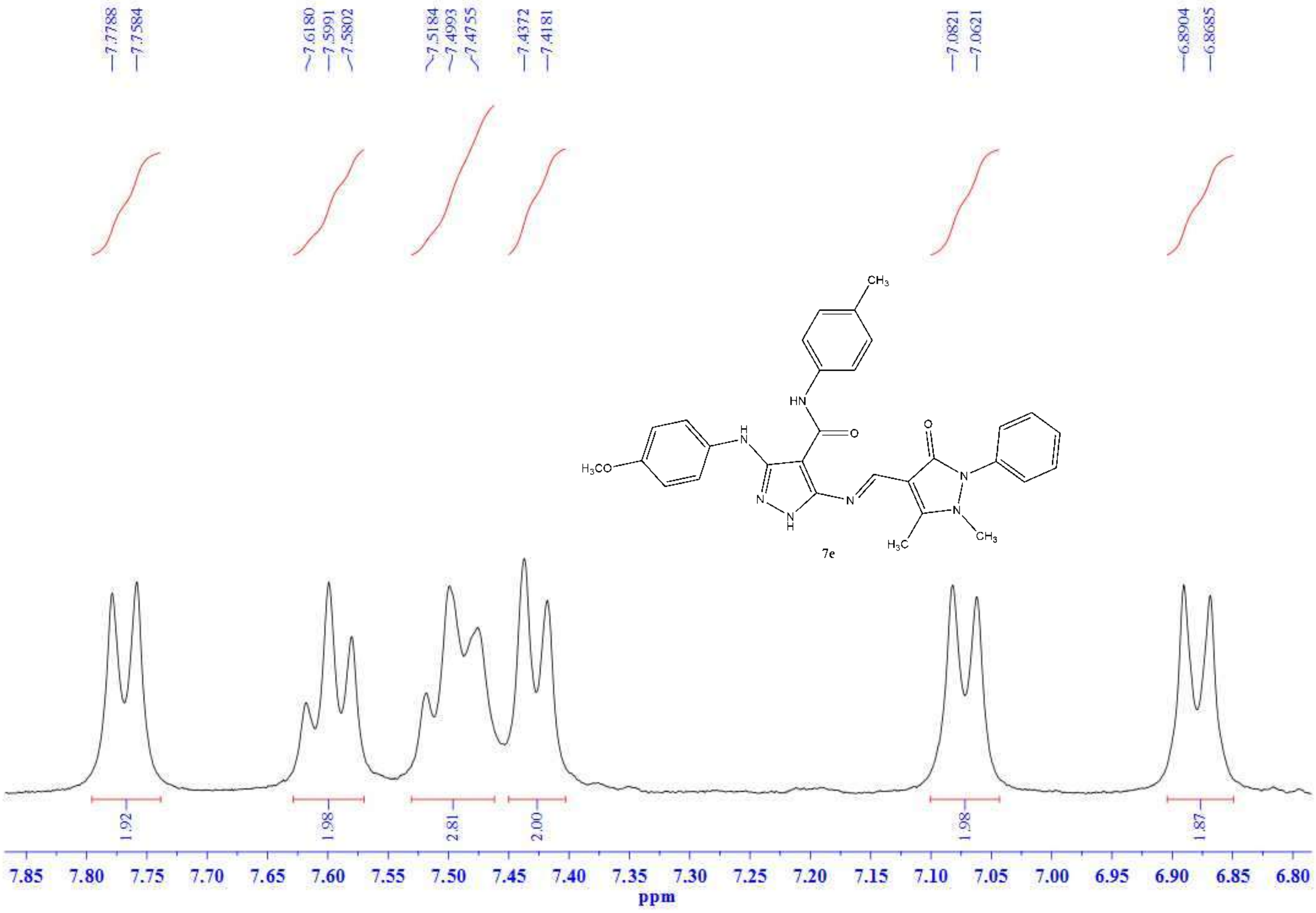

The ^{1}H NMR aromatic region spectrum of Schiff base **7e**

4.2. *In vitro* biological activities

1. Antioxidant activity

The total antioxidant capacity (TAC) was determined by analyzing the green phosphate/Mo^{5+} complex at a wavelength (λ) of 695 nm, following the procedure described by **Prieto *et al.*** Samples (at each concentration) were mixed with a reagent solution containing 0.3 N sulfuric acid, 28 mM sodium phosphate, and 4 mM ammonium molybdate. Methanol (80%) was used in place of the sample for the blank. The tubes were sealed and incubated in a boiling water bath for 90 minutes. After cooling to room temperature, the absorbance was measured at 695 nm against the blank. Ascorbic acid was used at the same concentrations as a standard. The antioxidant capacity was expressed as mg gallic acid equivalent per gram weight.

The iron reducing power was determined as μg/mL using the method proposed by **Oyaizu (1986)**. In brief, 1ml of the tested sample (at each concentration) was combined with 1mL of 200mM sodium phosphate buffer (pH 6.6) and 1mL of 1% potassium ferricyanide. The mixture was then incubated at 50°C for 20 minutes, followed by the addition of 1mL of trichloroacetic acid (10%). After centrifugation at 2000rpm for 10 minutes, the upper layer solution (2.5 mL) was mixed with 2.5 mL of double deionized water and 1mL of fresh ferric chloride (0.1%). The absorbance was measured at 700nm against a blank prepared without the sample. Ascorbic acid was used at the same concentrations as a standard. A high absorbance at 700nm indicates a higher reducing power in the reaction mixture.

2. Scavenging activity

2.1. DPPH radical-scavenging activity

The 1,1-Diphenyl-2-picryl-hydrazyl (DPPH) radical scavenging activities were evaluated using the method described by **Rahman *et al.*** An antioxidant substance capable of donating a hydrogen atom to a solution containing DPPH- can reduce the stable free radical, causing the solution to change color from violet to pale yellow. The remaining DPPH- radical was quantified by measuring the intensity of a light-purple colored DPPH methanol solution in the visible range at 518 nm using a spectroscopic method. Two milliliters of a DPPH solution (100 μM) in ethanol were mixed with 2 mL of the sample (at each concentration). The reaction mixture for each concentration was thoroughly vortexed and then incubated in the dark at room temperature for 30 minutes. The absorbance was then measured spectrophotometrically at 518 nm against a blank (ethanol). For the control, 2 mL of ethanol was added instead of the sample and run simultaneously with the test. Ascorbic acid was used at the same concentrations as a positive control. Percent inhibition of the DPPH free radical was calculated. The median inhibitory concentration (IC_{50}) for each tested compound was calculated using a series of concentrations (0, 0.75, 1.56, 3.125, 6.25, 12.5, 25, 50, and 100 μg/mL).

2.2. ABTS radical scavenging assay

The procedure for the 2,2'-azinobis-(3-ethylbenzothiazoline-6-sulfonic acid) (ABTS) assay followed the method suggested by **Arnao *et al.*** Stock solutions included ABTS solution (7 mM) and potassium persulfate solution (2.4 mM). The working solution was prepared by mixing the two stock solutions in equal quantities and allowing them to react at room temperature in a dark place for 14 hours. The solution was then diluted by mixing 1 mL of ABTS solution with 60 mL of methanol to obtain an absorbance of 0.706 ± 0.01 units at 734 nm using a spectrophotometer. Fresh ABTS solution was prepared for each assay. The tested samples (at each concentration) were allowed to react with 1 mL of the ABTS solution, and the absorbance was taken at 734 nm after 7 minutes using a spectrophotometer. The ABTS scavenging capacities of the samples were compared with that of ascorbic acid (at the same concentrations).

3. Anti-diabetic activity

This assay involved calculating the inhibition percentage (%) of α-amylase enzyme using method based on the technique demonstrated by **Wickramaratne *et al.*** with Acarbose as the standard drug. During the assay, 0.5 ml of each sample (at each concentration) was combined with 0.5 ml of α-amylase solution (0.5 mg/ml) and buffer (Na_2HPO_4/NaH_2PO_4 (0.02 M), NaCl (0.006 M) at pH 6.9). The mixture was then left at room temperature for 10 minutes before adding 200 μL of starch solution (1% in water (w/v) buffer (Na_2HPO_4/NaH_2PO_4 (0.02 M), NaCl (0.006 M) at pH 6.9)). The reaction was stopped by adding 200 μL of DNSA (coloring) reagent (12 g of sodium potassium tartrate tetrahydrate in 8.0 mL of 2 M NaOH and 20 mL of 96 mM of DNSA solution). The test tubes were then placed in a boiling water bath (100 °C) for 10 minutes and the mixture was cooled to room temperature and diluted with 5 mL of distilled water. The absorbance was measured at 540 nm using a UV-Visible spectrophotometer.

The inhibition percentage (%) of the α-glucosidase enzyme was determined using the method proposed by **Pistia-Brueggeman and Hollingsworth** with Acarbose as the standard drug. Five μL of the α-glucosidase solution (10 units mL^{-1}, 0.1moll^{-1} potassium phosphate buffer, pH 6.8) was pre-mixed with 10 μL of each sample (at each concentration). After incubation at 37.5 °C for 20 minutes, 10 μL of p-nitro phenyl glucopyranoside (pNPG, 10 mmoll^{-1}) as a substrate was added to the mixture to start the reaction. The reaction mixture was then incubated at 37.5°C for 30 minutes, followed by the addition of 650 μL of 1 moll^{-1} Na_2CO_3 solution to terminate the reaction. The amount of released product (p-nitro phenol) was measured at 410 nm using a UV spectrometer (UV-2550, Shimadzu, Japan) to estimate the enzymatic activity.

4. Anti-Alzheimer's activity

In this study, we assessed the inhibition percentage of the acetylcholinesterase (AChE) enzyme using Ellman's method with donepezil as the standard drug. For each run, 5 μL of Acetylthiocholine (ATCh) at a concentration of 0.5 mM, 5 μL of 5,5'-dithiobis-2-nitrobenzoic acid (DTNB) at a concentration of 0.03 mM, and 5 μL of each sample (at each concentration) were added to a flat bottom 96-well plate. The mixture was then incubated for 10 minutes at 30 °C. After incubation, 5 μL of AChE at a concentration of 0.3 U/mL was added to start the reaction, and the absorbance was measured at 412nm. A control run was also performed, which included all the components except for the test sample. The median inhibitory concentration (IC_{50}) of each tested sample was calculated by plotting a curve using a series of sample concentrations against the percent of AChE inhibition.

5. Anti-inflammatory activity

This assay involved determining the inhibition percentage (%) of protein denaturation and proteinase inhibition using diclofenac sodium as the standard non-steroidal anti-inflammatory drug, as prepared according to **Meera *et al.*** The percentage of protein denaturation inhibition was measured by mixing 0.5mL of the test control solution, prepared by combining 0.45 mL of bovine serum albumin (BSA) (5% w/v aqueous solution) with 0.05 mL of distilled water. Then, 0.05 mL of each sample (at each concentration) was added to 0.45 mL of distilled water to form the product control (0.5 mL). The pH value in all prepared solutions was adjusted to 6.3 using HCl (1N). All the samples were incubated at 37 ^{o}C for 20 min, and the temperature was then increased to 57 ^{o}C, maintaining the samples at that degree for 3 min. After cooling, 2.5 mL of phosphate buffer was added to the prepared solutions. The absorbance was determined at 416 nm using a UV-Visible spectrophotometer. The percentage of protein denaturation inhibition can be calculated.

The inhibition percentage of proteinase enzyme was assessed by combining 1 mL of each sample (at each concentration) with a reaction mixture containing 0.06 mg trypsin dissolved in 1 mL of 20 mM Tris HCl buffer (pH 7.4). The mixture was then incubated for 5 minutes at 37°C, followed by the addition of 1 mL of casein (0.8% w/v). After an additional 20 minutes of incubation, 2 mL of perchloric acid (70%) was added to stop the reaction. The cloudy suspension was then centrifuged,

and the absorbance of the supernatant was measured at 210 nm against buffer as the blank. The IC_{50} of each tested sample was calculated by plotting a curve using a series of sample concentrations against the percent of proteinase inhibition.

6. Cytotoxic activity

It will be assayed against human lung cancer (A549) and colon (Caco-2) cell line in comparison with normal lung (WI-38) cell line using 3-(4,5-dimethythiazol-2-yl)-2,5-diphenyl tetrazolium bromide (MTT) assay by determining the optical density (OD) at 570 nm according to the method suggested by **Vichai and Kirtikara**. The cells were dispensed in a 96-well sterile microplate (3 × 10^4 cells/ well), followed by their incubation at 37 °C with a series of different concentrations of 10 μL of each compound or doxorubicin (positive control, in DMSO) for 48 h in a serum free medium prior to the MTT assay. Subsequently, the media were carefully removed, and 40 μL of MTT (2.5 mg/mL) was added to each well and then incubated for an additional 4 h. Purple formazan dye crystals were solubilized by the addition of 200 μL of DMSO. The absorbance was measured at 570 nm using a SpectraMax Paradigm Multi-Mode microplate reader. The relative cell viability was expressed as the mean percentage of viable cells relative to the untreated control cells. All experiments were conducted in triplicate. Percent of the cell-growth inhibition (%) and the IC_{50} was calculated using IC_{50} calculation software. The concentrations used for calculating the IC_{50} depend on the type of cell lines. For both A549 and Caco-2 cell lines, the concentration series (0, 6.25, 12.5, 25, 50, and 100 μg/mL) was lower than those used for the WI-38 cell line (0, 31.125, 62.5, 125, 250, and 500 μg/mL).

7. The enzymatic activity

Activities of caspase-3 were measured by enzyme-linked immunosorbent assay (ELISA) using the Invitrogen caspase-3 (Active) (human) ELISA kit (96 tests) from Invitrogen Corporation, following the manufacturer's instructions. Activities of Bcl-2 were measured using the Invitrogen Zymed Bcl-2 ELISA Kit (96 tests) from Invitrogen Corporation, following the manufacturer's instructions.

Statistical analysis

All experiments were conducted in triplicate and repeated in three different days. Data calculated from three replicates and presented as mean ± SE.
